# 74th Congress of the Italian Society of Pediatrics: Rome, Italy. 12-16 June 2018

**DOI:** 10.1186/s13052-018-0581-y

**Published:** 2018-12-20

## A1 Nutritional aspects in Neonatal Intensive Care Unit

### Massimo Agosti, Laura Morlacchi, Francesco Tandoi, Angela Bossi

#### S.C. Neonatologia, Terapia Intensiva Neonatale e Pediatria Verbano, ASST dei Sette Laghi - Polo universitario, Ospedale "F. Del Ponte", Varese, Italy

##### **Correspondence:** Massimo Agosti (massimo.agosti@asst.settelaghi.it)

Neonatologists still face the challenge to provide optimal nutritional care to preterm infants and to limit the postnatal growth failure that preterm infants still experiment [1].

Inadequate parenteral and enteral intakes and the fear of metabolic intolerance lead to a cumulative nutrients’ deficit in early postnatal period [2]. Limiting early malnutrition is of major importance since poor postnatal growth in preterm infants has been associated with impaired neurodevelopmental outcomes [3,4] and altered body composition development [5]. Body composition of preterm infants seems to be characterized by a lack of fat-free mass deposition [6], which, in turns, is determinant for organ growth and development, particularly the brain. Increasing findings actually support an association between postnatal fat-free mass accretion and neurodevelopment [7].

The transition to extrauterine life inevitably contributes to the higher nutritional needs of the preterm infants [5]. It has been demonstrated that the resting energy expenditure of preterm infants increases by 140% in the first six weeks of postnatal age whereas that of term infants increases by 47% [8]. In addition, the major clinical comorbidities that they often experiment (sepsis, neurological impairment, cardiac diseases, surgical complications, administration of medications and the different environmental conditions they are exposed to) necessarily affect infants’ nutritional requirements [9].

The application of standardized nutritional procedures and the attention on individual requirements are of major importance. Several efforts in the last year have been addressed to limit the infants’ postnatal growth restriction: nutritional strategies based on more aggressive parenteral nutrition, adequate weaning from parenteral nutrition and optimization of enteral nutrients administration have been reported to improve growth velocity during hospital stay in neonatal intensive care unit [10,11].

Human milk is the first choice for the nutritional support in preterm infants [12]: its several health benefits on immunological, gastrointestinal and neurodevelopmental functions have been deeply reported. Fortification of human milk is required to meet the high preterm infants’ nutritional requirements [13]. Recent studies suggest that the non-nutritive oral administration of colostrum is safe and it could positively affect development of innate immunity in extremely preterm infants [14].

Oral feeding is the final milestone to achieve for an infant before leaving hospital; as a consequence its implementation is of huge interest. The non-nutritive sucking stimulation, the promotion of human milk use, a cue-based feeding approach and the limitation of the negative experiences the hospitalized infants are expose to, can lead to their earlier achievement of full oral feeding [15].

**References**

1. Cooke RJ. Improving growth in preterm infants during initial hospital stay: principles into practice. Arch Dis Child Fetal Neonatal Ed. 2016;101:F366-70.
2. Brennan AM, Murphy BP, Kiely ME. Optimising preterm nutrition: present and future. Proc Nutr Soc. 2016;75:154-61.
3. Ehrenkranz RA, Dusick AM, Vohr BR, Wright LL, Wrage LA, Poole WK. Growth in the neonatal intensive care unit influences neurodevelopmental and growth outcomes of extremely low birth weight infants. Pediatrics 2006;117:1253e61.
4. Ramel SE, Demerath EW, Gray HL, Younge N, Boys C, Georgieff MK. The relationship of poor linear growth velocity with neonatal illness and two-year neurodevelopment in preterm infants. Neonatology. 2012;102:19-24.
5. Giannì ML, Roggero P, Piemontese P, Orsi A, Amato O, Taroni F, Liotto N, Morlacchi L, Mosca F. Body composition in newborn infants: 5-year experience in an Italian neonatal intensive care unit. Early Hum Dev. 2012;88:S13-7.
6. Johnson MJ, Wootton SA, Leaf AA, Jackson AA. Preterm birth and body composition at term equivalent age: a systematic review and meta-analysis. Pediatrics. 2012;130:e640-9.
7. Ramel SE, Gray HL, Christiansen E, Boys C, Georgieff MK, Demerath EW. Greater Early Gains in Fat-Free Mass, but Not Fat Mass, Are Associated with Improved Neurodevelopment at 1 Year Corrected Age for Prematurity in Very Low Birth Weight Preterm Infants. J Pediatr. 2016;173:108-15.
8. Bauer J, Werner C, Gerss J. Metabolic rate analysis of healthy preterm and full-term infants during the first weeks of life. Am J Clin Nutr. 2009;90:1517-24.
9. Hulzebos CV, Sauer PJ. Energy requirements. Semin Fetal Neonatal Med. 2007;12:2-10.
10. Roggero P, Giannì ML, Orsi A, Amato O, Piemontese P, Liotto N, Morlacchi L, Taroni F, Garavaglia E, Bracco B, Agosti M, Mosca F. Implementation of nutritional strategies decreases postnatal growth restriction in preterm infants. PLoS One. 2012;7:e51166.
11. Loÿs CM , Maucort-Boulch D, Guy B, Putet G, Picaud JC, Haÿs S. Extremely low birthweight infants: how neonatal intensive careunit teams can reduce postnatal malnutrition and prevent growth retardation. Acta Paediatr. 2013;102:242-8.
12. American Academy of Pediatrics Section on Breastfeeding: Breastfeeding and the use of human milk. Pediatrics. 2012;129:827-41.
13. Morlacchi L, Mallardi D, Giannì ML, Roggero P, Amato O, Piemontese P, Consonni D, Mosca F. Is targeted fortification of human breast milk an optimal nutrition strategy for preterm infants? An interventional study. J Transl Med. 2016;14:195.
14. Lee J, Kim HS, Jung YH, Choi KY, Shin SH, Kim EK, Choi JH. Oropharyngeal colostrum administration in extremely premature infants: an RCT. Pediatrics. 2015;135:e357-66.
15. Shaker CS. Cue-based feeding in the NICU: using the infant's communication as a guide. Neonatal Netw. 2013;32:404-8.

## A2 Retrocecal appendicitis

### Ivan P Aloi, Arianna Bertocchini, Alessandro Inserra

#### Department of Surgery, Bambino Gesù Children’s hospital , Rome, 00164, Italy

##### **Correspondence:** Ivan P Aloi (ivanpietro.aloi@opbg.net)

Appendicitis is the most common acute surgical condition of the abdomen. Despite technological advances, the diagnosis of appendicitis is still based primarily on the patient's history and the physical examination. However, some patients may have atypical symptoms and physical findings that may lead to a delay in diagnosis and increased complications. Atypical presentation may be related to the position of the appendix.

The vermiform appendix may occupy several positions in relation to the cecum. The most common positions are descending intraperitoneal (31%-74%) and retrocecal (26%-65%). When the appendix is in the retrocecal position, the signs and symptoms of acute appendicitis may be atypical and mimic pathology in the right flank and hypochondrium, such as acute cholecystitis, diverticulitis, acute gastroenteritis, ureter colic, acute pyelonephritis, and irritable bowel syndrome.

Prompt diagnosis and surgical referral may reduce the risk of perforation and prevent complications. The mortality in non-perforated appendicitis is a rare event, but it may be more significant in very young and elderly patients, in whom diagnosis may be delayed.

Appendectomy may be performed by laparotomy or laparoscopy. Diagnostic laparoscopy may be helpful in equivocal cases or in women of childbearing age, while laparoscopic appendectomy is becoming the preferred approach for any kind of appendicitis.

The laparoscopic intervention has the advantages of decreased postoperative pain, faster recovery, earlier return to normal activity and better cosmetic results. This benefit has been shown through all age groups, but elderly patients in particular experience an advantage with the minimally invasive approach [1].

**References**

1. Wang YC, Yang HR, Chung PK, Jeng LB, Chen RJ. Laparoscopic appendectomy in the elderly. Surg Endosc. 2006;20:887-88.

## A3 Chronic insomnia of childhood: clinical assessment of different phenotypes

### Marco Angriman (marco.angriman@gmail.com)

#### Child Neurology Unit, Hospital of Bolzano, Bolzano, Italy

Bedtime problems and night awakenings are common cause of paediatric consultation and sleep disorders in children can compromise quality of life of both children and families.

It is known that chronic sleep deprivations is associated with poorer developmental outcome, overweight and behavioral disturbances.

Clinicians should incorporate questions about sleep into their routine health assessment, and the assessment of insomnia should follow a medical approach. Primary and secondary contributing factors should be assessed, as well as maladaptive behaviors related to sleep.

In order to identify childhood insomnia clinicians must be willing to observe children and listen to them closely. Symptoms of pediatric insomnia may include: difficulties falling asleep, episodes of waking up in the night with inability to go back to sleep or waking up early in the morning.

Patients may be chronically tired or show academic or behavioral difficulties, with hyperactive components.

A careful examination of sleep/wake schedule, abnormal movements or behavior during sleep, and daytime consequences of sleep disruption or deprivation is mandatory.

Sleeping environment and bedtime routines should be examined to identify behavioral issues related to sleep. Polysomnography is not routinely indicated for children with insomnia, but actigraphy can give an objective estimation of sleep parameters.

All medical treatments for chronic pediatric insomnia are off label, but advances in clinical evidence are emerging; behavioral therapies like cognitive behavioral therapy provide non-pharmacologic alternatives that help rewire disrupted sleep patterns and sleep hygiene should be implemented.

The aim of the present talk is to summarize recent advances in the field of clinical pediatric sleep medicine: a phenotype-based classification of pediatric insomnia, based on both genetic and clinical aspects is proposed; clinical evaluation, short time and chronic sequelae are described; several tools for clinical assessment and treatment options of paediatric insomnia, useful in the daily clinical practice of pediatricians, are presented.

## A4 The future perspectives of allergen immunotherapy in childhood

### Stefania Arasi^1,2^, Giovanni B Pajno^1^

#### ^1^Allergy Unit- Department of Pediatrics, University of Messina, Messina, I-98124, Italy; ^2^SIAF-Schweizerischers Institut für Allergie-und Asthmaforschung, Davos, CH-7270, Switzerland

##### **Correspondence:** Stefania Arasi (stefania.arasi@unime.it)

Allergen immunotherapy (AIT) is the only currently available treatment that targets the etio-pathophysiology and may modulate the natural history of IgE-mediated diseases [1-6]. Childhood represents the best timeframe to influence the immune system and alter the progression of allergic diseases during the early phases of respiratory allergic diseases [3,6]. There is evidence that AIT constitutes a suitable therapeutic option to be considered in patients suffering from allergic rhino-conjunctivitis (AR) due to grass pollen and wishing to take advantage of AIT’s long-term effect (at least one year after cessation of AIT course) on AR and its potential preventive effect on asthma development [1]. AIT might be a strategy to prevent the development of new sensitization(s) [3,6]. Furthermore, a growing body of evidence support the use of oral immunotherapy as a promising treatment option in children with persistent IgE- mediated food allergy [2,5]. The efficacy of AIT is under investigation also in patients with extrinsic atopic dermatitis, currently with controversial results.

Overall, the interest and the attention to AIT treatment are currently fervent and increasing. However, there are still some methodological criticisms and gaps to be filled in the current body of evidence: a) the regimen of administration and the amount of the maintenance dose are both largely variable; b) the protocols of administration are not standardized; c) the description and classification of side effects is variable among studies and needs to be standardized; d) quality of life and evaluation of health economics are overall missing. All these aspects make difficult to compare each study with another. In addition, the content of major allergen(s) remains largely variable among manufacturers and the availability of AIT products differences among countries. The development of integrated care pathways incorporating (educating and training) primary and secondary care, as well as the availability of high quality AIT products and global actions aimed to develop a harmonized international approach to regulate AIT products are awaited in order to implement AIT in clinical practice. Well-designed studies are awaited in the near future in order to overcome the current gaps in the evidence and furtherly promote implementation strategies with the final goal of a “precision medicine/prevention”, tailored on each specific clinical sub-group.

**References**

1. Roberts G, Pfaar O, Akdis CA, Ansotegui IJ, Durham SR, Gerth van Wijk R, Halken S, Larenas-Linnemann D(, Pawankar R, Pitsios C, Sheikh A, Worm M, Arasi S, Calderon MA, Cingi C, Dhami S, Fauquert JL, Hamelmann E, Hellings P, Jacobsen L, Knol EF, Lin SY, Maggina P, Mösges R, Oude Elberink JNG, Pajno GB, Pastorello EA, Penagos M, Rotiroti G, Schmidt-Weber CB, Timmermans F, Tsilochristou O, Varga EM, Wilkinson JN, Williams A, Zhang L, Agache I, Angier E, Fernandez-Rivas M, Jutel M, Lau S, van Ree R, Ryan D, Sturm GJ, Muraro A. EAACI Guidelines on allergen immunotherapy: allergic rhinoconjunctivitis. Allergy. 2018;73:765-798.
2. Pajno GB, Fernandez-Rivas M, Arasi S, Roberts G, Akdis CA, Alvaro-Lozano M, Beyer K, Bindslev-Jensen C, Burks W, Ebisawa M, Eigenmann P, Knol E, Nadeau KC, Poulsen LK, van Ree R, Santos AF, du Toit G, Dhami S, Nurmatov U, Boloh Y, Makela M, O'Mahony L, Papadopoulos N, Sackesen C, Agache I, Angier E, Halken S, Jutel M, Lau S, Pfaar O, Ryan D, Sturm G, Varga EM, van Wijk RG, Sheikh A, Muraro AEAACI Guidelines on allergen immunotherapy: IgE-mediated food allergy. Allergy. 2018;73:799-815.
3. Halken S, Larenas-Linnemann D, Roberts G, Calderón MA, Angier E, Pfaar O, Ryan D, Agache I, Ansotegui IJ, Arasi S, Du Toit G, Fernandez-Rivas M, Geerth van Wijk R, Jutel M, Kleine-Tebbe J, Lau S, Matricardi PM, Pajno GB, Papadopoulos NG, Penagos M, Santos AF, Sturm GJ, Timmermans F, van Ree R, Varga EM, Wahn U, Kristiansen M, Dhami S, Sheikh A, Muraro A.. EAACI guidelines on allergen immunotherapy: prevention of allergy. Pediatr Allergy Immunol. 2017;28:728-745.
4. Dhami S, Nurmatov U, Arasi S, Khan T, Asaria M, Zaman H, Agarwal A, Netuveli G, Roberts G, Pfaar O, Muraro A, Ansotegui IJ, Calderon M, Cingi C, Durham S, van Wijk RG, Halken S, Hamelmann E, Hellings P, Jacobsen L, Knol E, Larenas-Linnemann D, Lin S, Maggina P, Mösges R, Oude Elberink H, Pajno G, Panwankar R, Pastorello E, Penagos M, Pitsios C, Rotiroti G, Timmermans F, Tsilochristou O, Varga EM, Schmidt-Weber C, Wilkinson J, Williams A, Worm M, Zhang L, Sheikh A. Allergen immunotherapy for allergic rhinoconjunctivitis: a systematic review and meta-analysis. Allergy. 2017;72:1597-1631.
5. Nurmatov U, Dhami S, Arasi S, Pajno GB, Fernandez-Rivas M, Muraro A, Roberts G, Akdis C, Alvaro-Lozano M, Beyer K, Bindslev-Jensen C, Burks W, du Toit G, Ebisawa M, Eigenmann P, Knol E, Makela M, Nadeau KC, O'Mahony L, Papadopoulos N, Poulsen LK, Sackesen C, Sampson H, Santos AF, van Ree R, Timmermans F, Sheikh A.Allergen immunotherapy for IgE-mediated food allergy: a systematic review and meta-analysis. Allergy. 2017;72:1133-1147.
6. Kristiansen M, Dhami S, Netuveli G, Halken S, Muraro A, Roberts G, Larenas-Linnemann D, Calderón MA, Penagos M, Du Toit G, Ansotegui IJ, Kleine-Tebbe J, Lau S, Matricardi PM, Pajno G, Papadopoulos NG, Pfaar O, Ryan D, Santos AF, Timmermanns F, Wahn U, Sheikh A.Allergen immunotherapy for the prevention of allergy: A systematic review and metaanalysis. Pediatr Allergy Immunol 2017;28:18-29.

## A5 A platform of new generation

### Sergio Bella, Clarissa Paglia, Fabio Murgia

#### Cystic Fibrosis Unit, Bambino Gesù Children’s Hospital, Rome, Italy

##### **Correspondence:** Sergio Bella (sergio.bella@opbg.net)

**Background**

The improvements in the sanity field have led to an increase of chronic diseases in spite of acute ones. This trend has generated the knowledge of co-morbidity of the primary disease. Furthermore, as known, chronic diseases affect mostly adults, who suffer more than children the hospitalization phase. This is the reason why, recently, the telemonitoring, which is a kind of remote assistance, is really starting to catch on. In a first phase just single devices have been adopted. Nowadays technologies let us to use platforms, based on a distributed system. A distributed system is a model in which components communicate and coordinate their actions by passing messages through bluetooth connection. The components interact with each other in order to achieve a common goal.

**Materials and methods**

The Vivisol telemedicine platform allows the unit to perform both telemonitoring and telediagnostics. The integrated medical devices, which are CE certified, are the following: spirotel spirometer/spirodoc spirometer, nonin 9560 pulse oximeter, HS5 iHealth bluetooth scale, 3MLitman stethoscope, ForaCare thermometer, iHealth weight glucometer, iHealth sphygmomanometer.

Patients will make measurements from the home through devices. All the measurements will be read by the doctor or by a 24/24 operating call center that will perform the triage and then, if needed, doctors will be called

**Results**

It is expected, as already seen in other projects, to obtain important results that can be summarized in the following points: fewer accesses to the hospital, lowering of vital parameters, autonomy of the patient who will learn to manage the symptomatology better.

**Conclusions**

The introduction of a platform, which can be easily assembled and used, guarantees the patient greater security in the management of home-based therapies and an immediate recognition of critical problems, which will allow to have a clinical stability and therefore also a reduction in cost management by the national health system.

## A6 The role of family pediatrician

### Aldo Bellocchi (a.bellocchi@libero.it)

#### ASL Roma 4, Roma, Italy

Obstructive sleep apnoea (OSA) is a common condition of childhood with significant associated morbidity. The comprehensive evaluation of children who present with suggestive symptoms involves the overnight recording and assessment of both sleep and respiration by polysomnography in a sleep laboratory. The common symptom of pediatric OSAHS include snoring, restless sleep, struggling to breathe, abnormal paradoxical chest/abdomen motion, mouth breathing, failure-to-thrive. Obesity and excessive daytime sleepiness are present. The studies demonstrate that pediatric OSAHS are characterized by partial upper airway obstruction, more or less apnea and associated with staged desaturation. Apnea hypopnea index (AHI), lowest oxygen saturation (LSaO2) and desaturation index below 90% (SIT90%) are very important factors to measure about serious degree of pediatric OSAHS. Physical examination, subjective symptoms and clinical history, these three items were used to create a sleep clinical score (SCS). SCS may effectively be used to screen patients as candidates for polysomnography study for suspected OSA syndrome, and to enable those with a mild form of sleep disordered breathing to receive early treatment.

## A7 Health and environment

### Sergio Bernasconi, Silvia Cesari

#### University of Parma, Parma, Italy

##### **Correspondence:** Sergio Bernasconi (Sbernasconi3@gmail.com)

The environment in which we live has been progressively contaminated by numerous man-produced chemical substances from a variety of sources that are responsible for damage to our ecosystem and population health. In particular, numerous epidemiological studies and biological models have suggested the possible interference of these chemical substances on hormonal systems in human beings. Many international organizations that address environmental health issues have evidenced the importance of endocrine disruptors, defining them as “any exogenous substance or material that can alter one or more functions of the endocrine system, subsequently causing adverse effects on the health of a human being or his progeny”. These organizations have stressed the need to strengthen research in this field and the importance of applying, in the absence of definitive data, the principle of precaution particularly in critical biological periods such as the prenatal period and the first years of postnatal life.

Presently, more than 100000 chemical substances exist on the market and at least 1000 of these can act as endocrine disruptors; the most common are identified as pesticides, industrial products (polychlorinated biphenyl, alkylphenols, phthalates) and plant-derived substances such as phytoestrogens.

The negative effects that endocrine disruptors have on health are multiple: from prenatal damage during the development of the central nervous system with consequent cognitive and behavioral impairment, to obesity and type 2 diabetes mellitus. Scientific evidence also indicate endocrine disruptors as substances that can reduce fertility through a reduction in sperm count, cause anomalies in the development of external genitalia in males (cryptorchidism), and alter the morphology of the penis (reduced dimensions or anomalous urethral opening). In females these substances seem to contribute to precocious breast development (thelarche), which has been evidenced in several countries including Italy. It is therefore important for the Pediatrician to transmit concrete information regarding this subject to patients and families in order to apply correct behaviors that may reduce exposure to endocrine disruptors.

## A8 How a vaccine is developed

### Luigi R Biasio (lrbiasio@gmail.com)

#### Contract Lecturer in Vaccinology, Rome, Italy

The development of a new vaccine aims at meeting medical and public health needs, making use of scientific knowledge and technological achievements [1]. Inactivation and attenuation still remain the main processes of vaccine production, but different modern techniques in molecular biology and genetic engineering led to the development and manufacturing of new inactivated antigens and attenuated pathogens [2].

Vaccine manufactured with new technologies are used in the immunization practice for some time. Hepatitis B vaccines produced in yeast by recombinant DNA technology have now been available for over twenty-five years, replacing those based on purification of plasma of infected individuals, and consenting the implementation of large vaccination programs [3]. Recently, other recombinant vaccines have been created (HPV, malaria, Herpes Zoster and others).

Moreover, the sequencing of microbial genomes allowed the identification of new protective factors and the expression of the predicted genes in bacteria: the resulting proteins can be used to immunize against specific diseases (reverse vaccinology) [4]. Also, vectored vaccines were developed utilizing non-pathogenic viruses as carriers that incorporate and express the gene for a pathogen [5] (Dengue, HIV, etc.).

Despite these attainments, production of some vaccines extensively employed in public health programs, such as measles, remains anchored to “old” technologies, and development of vaccines against important still non-preventable pathologies has been unsuccessful so far, although several new projects are ongoing.

The pharmaceutical and clinical development of new vaccines lasts several years or indefinitely [6]. Indeed, their development represents a sort of continuum: following the marketing authorization, effectiveness studies, post-marketing surveillance and phase IV clinical trials may lead to changes in the pharmaceutical formulation and to applications for new indications. In the recent history, different examples show how the scientific and technological progress has allowed researchers and producers not only to develop new vaccines, but also to re-develop those needing improvement, thus increasing the potentialities and benefits of vaccination.

**References**

1. Wechsler J. Modern manufacturing key to more effective vaccines. Pharmaceutical technology 2018; 4:16–18.
2. Plotkin SA. Vaccines: past, present and future. Nat Med. 2005:11; S5–S11.
3. Bonanni P, Santos JI. Vaccine evolution. Chapter 1. In: Garçon N, Stern PL, Cunningham AL, editors. Understanding modern vaccines, perspectives in vaccinology, Volume 1. Amsterdam: Elsevier; 2011. 1–24.
4. Rappuoli R. Reverse vaccinology, a genome-based approach to vaccine development. Vaccine 2001; 19:2688-91.
5. Ura T, Okuda K, Shimada M. Developments in viral vector-based vaccines. Vaccines. 2014;2:624-641.
6. Leroux-Roels G, Bonanni P, Tantawichien T, Zepp F. Vaccine development. Chapter 5. In: Garçon. N, Stern PL, Cunningham AL, editors. Understanding modern vaccines, perspectives in vaccinology, Volume 1. Amsterdam: Elsevier; 2011. 115-150.

## A9 Sepsis in children: the new surviving sepsis campaign guidelines

### Paolo Biban, Marcella Gaffuri, Stefania Spaggiari, Rossella Frassoldati, Silvia Carlassara, Davide Silvagni

#### Department of Neonatal and Paediatric Critical Care, Verona University Hospital, Verona, Italy

##### **Correspondence:** Paolo Biban (paolo.biban@aovr.veneto.it)

Sepsis is associated with substantial morbidity in hospitalized children, remaining one of the leading causes of death, even in advanced countries.[1] Data from 15 Italian Pediatric Intensive Care Units (PICUs) reported mortality as high as 50% in children with septic shock.[2] To improve such dismal outcome, specific protocols for the management of severe sepsis and septic shock have been adopted by several centers worldwide. Most of these protocols are derived from the Surviving Sepsis Campaign Guidelines (SSCG), which were first released in 2008 and regularly updated since then.[3,4] Key elements for appropriate treatment of sepsis are similar both in adults and children, including early recognition and rapid establishment of early goal-directed therapy. A small set of evidence-based practices, such as optimal fluid resuscitation, administration of broad spectrum antibiotics and use of inotropic or vasopressor agents, constitute a “sepsis bundle”, a tool to be timely applied adopting specific algorithms.[5,6] Indeed, implementation of sepsis bundles by quality improvement strategies have been associated with improved outcomes in the emergency department (ED), such as reduced mortality and length of hospital stay.[7,8] However, implementation and maintenance of adherence of SSCG are not easy tasks.

Workman et al. evaluated the association between timely delivery of therapy (three elements of a bundle, to be accomplished within 1 hour) and development of multiple organ failure in 321 children presenting in ED with septic shock.[9] Of note, only 36% of patients received all bundle measures within 1 hour. Interestingly, the majority of remaining patients did so within 3 hours, showing comparable primary and secondary outcomes, raising doubts about the need to keep the “septic shock clock” always so strict.[10]

In a prospective study, Paul et al. investigated adherence to SSCG in their pediatric ED, looking at five time-specific goals, i.e. early recognition, vascular access, fluids, vasopressors, and antibiotic administration. Of note, just a minority of patients were treated according to the SSCG. Yet, after launching vigorous quality improvement initiatives among their ED staff, they achieved an impressively higher and sustained adherence to the SSCG algorithm.[7]

In conclusion, early recognition and rapid protocolized treatment are essential for children with septic shock. A timely application of sepsis bundles may markedly improve performance in the management of these very sick patients, potentially ameliorating their outcome. Further studies are needed to provide stronger evidence about the effectiveness of sepsis bundles, as well as to consistently increase the overall adherence to the new sepsis guidelines.

**References**

1. Hartman ME, Linde-Zwirble WT, Angus DC, Watson RS. Trends in the epidemiology of pediatric severe sepsis. Pediatr Crit Care Med. 2013; 14:686–93.

2. Wolfler A, Silvani P, Musicco M, Antonello M, Salvo I. Incidence of and mortality due to sepsis, severe sepsis and septic shock in Italian Pediatric Intensive Care Units: a prospective national survey. Intensive Care Med. 2008; 34:1690-7.

3. Dellinger RP, Levy MM, Rhodes A, Annane D, Gerlach H, Opal SM, Sevransky JE,Sprung CL, Douglas IS, Jaeschke R, Osborn TM, Nunnally ME, Townsend SR, Reinhart K, Kleinpell RM, Angus DC, Deutschman CS, Machado FR, Rubenfeld GD, Webb S, Beale RJ, Vincent JL, Moreno R; Surviving Sepsis Campaign Guidelines Committee including The Pediatric Subgroup. Surviving Sepsis Campaign: international guidelines for management of severe sepsis and septic shock, 2012. Intensive Care Med. 2013; 39:165-228.

4. Rhodes A, Evans LE, Alhazzani W, Levy MM, Antonelli M, Ferrer R, et al. Surviving sepsis campaign: international guidelines for management of sepsis and septic shock: 2016. Crit Care Med. 2017; 45:486-552.

5. Davis AL, Carcillo JA, Aneja RK, Deymann AJ, Lin JC, Nguyen TC, Okhuysen-Cawley RS, Relvas MS, Rozenfeld RA, Skippen PW, Stojadinovic BJ, Williams EA, Yeh TS, Balamuth F, Brierley J, de Caen AR, Cheifetz IM, Choong K, Conway E Jr, Cornell T, Doctor A, Dugas MA, Feldman JD, Fitzgerald JC, Flori HR, Fortenberry JD, Graciano AL, Greenwald BM, Hall MW, Han YY, Hernan LJ, Irazuzta JE, Iselin E, van der Jagt EW, Jeffries HE, Kache S, Katyal C, Kissoon NT, Kon AA, Kutko MC, MacLaren G, Maul T, Mehta R, Odetola F, Parbuoni K, Paul R, Peters MJ, Ranjit S, Reuter-Rice KE, Schnitzler EJ, Scott HF, Torres A Jr, Weingarten-Abrams J, Weiss SL, Zimmerman JJ, Zuckerberg AL. American college of critical care medicine clinical practice parameters for hemodynamic support of pediatric and neonatal septic shock. Crit Care Med. 2017; 45:1061-1093.

6. Biban P, Gaffuri M, Spaggiari S, Zaglia F, Serra A, Santuz P. Early recognition and management of septic shock in children. Pediatr Rep. 2012;4:e13.

7. Paul R, Melendez E, Stack A, Capraro A, Monuteaux M, Neuman MI. Improving adherence to PALS septic shock guidelines. Pediatrics. 2014;133:e1358–66.

8. Larsen GY, Mecham N, Greenberg R. An emergency department septic shock protocol and care guideline for children initiated at triage. Pediatrics. 2011;127:e1585–92.

9. Workman JK, Ames SG, Reeder RW, Korgenski EK, Masotti SM, Bratton SL, Larsen GY. Treatment of pediatric septic shock with the surviving sepsis campaign guidelines and PICU patient outcomes. Pediatr Crit Care Med. 2016; 17:e451-e458.

10. Biban P. Pediatric septic shock in the emergency department: can we set the alarm clock a little forward? Pediatr Crit Care Med. 2016; 17:1011-1012.

## A10 Respiratory failure in children

### Elisabetta M.C. Bignamini, Elena Nave, Irene Esposito

#### Pneumologia pediatrica – Città della Salute e della Scienza di Torino – Presidio Ospedale Infantile Regina Margherita (OIRM), Torino, 10126, Italia

##### **Correspondence:** Elisabetta M.C. Bignamini (ebignamini@cittadellasalute.to.it)

Respiratory failure is a condition of inadequate gas exchange. There is a wide range of causes, in children, leading to acute or chronic respiratory failure due either to progressive lung disease, or to respiratory pump function deficit that can be grouped into three main categories: a loss of central respiratory drive to breathe; ineffective thoracic musculoskeletal function; disorders of the respiratory tract [1,2].

The latter group includes children with progressive neuromuscular disorders (NMD). These patients undergo major respiratory complications for many reasons including inspiratory muscles weakness and ineffective cough; scoliosis due to decreased muscle support; decreased spontaneous movement, which reduces the normal redistribution of ventilation; high prevalence of gastro-oesophageal reflux and weakness of the facial, oropharyngeal, and laryngeal muscles that can result in a compromised swallow and secretion clearance.

It has been reported the effect of upper respiratory tract infections on reductions in respiratory muscle strength and, consequently, cause symptoms like shortness of breath, reduction in vital capacity, and acute hypercapnia. Low respiratory infections increase respiratory load, increasing the stiffness of the lung (as atelectasis) and increasing respiratory rate. [3,4,5].

All NMD patients must learn appropriate airway clearance techniques and introduce cough-machine. Patients with limited respiratory function must also be trained in Non-Invasive Ventilation (NIV). It is important to intercept, treat and prevent respiratory infections because the rate of them affects the child and family’s quality of life. Discussions about prognosis and how far treatment should go in the event of deterioration should take place in every progressive NMD and patient with progressive chronic respiratory failure, with active involvement of the patient and family [6,7].

Receiving support from a multidisciplinary care medical team can make a difference in the life of the patient with respiratory failure and his/her family. Child with respiratory failure, especially if ventilated, calls into question the very bases of biomedicine, which is often strict, to propose a peer relationship (health care professionals, social workers, patients and families) in which different knowledge intersect essentially to sustain the life, and the quality of life, of the child [8].

In paediatric clinical practice the treatment of respiratory failure raises ethical questions about the utility and limitations of therapeutic intervention, particularly for children dependent on technology; home caregivers’ burden and, because children with respiratory failure often are not or cannot be involved in healthcare, parents’ and guardians’ role in decision making and proxy consent for these children’s healthcare choice [9, 10].

**References**

1. Lamba TS, Sharara RS, Singh AC, Balaan M. Pathophysiology and classification of respiratory failure. Crit Care Nurs Q. 2016; 39:85-93.
2. Eber E, Midulla F. Handbook of Paediatric Respiratory Medicine. European Respiratory Society – ERS Ed. 2013.
3. Khirani S, Colella M, Caldarelli V, Aubertin G, Boulè M, Forin V, Ramirez A, Fauroux B. Longitudinal course of lung function and respiratory muscle strength in spinal muscular atrophy type 2 and 3. Eur J Paediatr Neurol. 2013; 17:552-60.
4. Racca F, Mongini T, Wolfer A, Vianello A, Cutrera R, Del Sorbo L, Capello EC, Gregoretti C, Massa R, De Luca D, Conti G, Tegazzin V, Toscano A, Ranieri VM. Recommendations for anesthesia and perioperative management of patients with neuromuscular disorders. Minerva Anestesiol. 2013; 79:419-33.
5. Panitch HB. Diurnal hypercapnia in patients with neuromuscular disease. Paediatr Respir Rev. 2010; 11:3-8.
6. Bach JR, Gonçalves MR, Hon A, Ishikawa Y, De Vito EL, Prado F, Dominiquez ME. Changing trends in the management of end-stage neuromuscular respiratory muscle failure: recommendations of an international consensus. Am J Phys Med Rehabil. 2013; 92:267-77.
7. McCrea N, O’Donnell R, Brown R. Outpatient respiratory management of the child with severe neurological impairment. Arch Dis Child Educ Pract Ed. 2013; 98:84-91.
8. Mangera Z, Panesar G, Makker H. Practical approach to management of respiratory complications in neurological disorders. Int J Gen Med. 2012; 5:255-63.
9. Carnevale FA, Alexander E, Davis M, Rennick J, Troini R. Daily living with distress and enrichment: the moral experience of families with ventilator-assisted children at home. Pediatrics. 2006; 117:48-60.
10. de Vos MA, Seeber AA, Gevers SKM, Bos AP, Gevers F, Williems DL. Parents who wish no further treatment for their child. JME. 2015; 41:195-200.

## A11 Smarthpone and tablets exposure in early childhood

### Elena Bozzola^1^, Giulia Spina^1^, Margherita Ruggiero^1^, Lugi Memo^2^, Rino Agostiniani^3^, Mauro Bozzola^4^, Giovanni Corsello^5^, Alberto Villani^1^

#### ^1^University Department, Pediatric and Infectious Disease Unit, Children Hospital Bambino Gesù, Rome, Italy; ^2^Pediatric Department, S. Martino Hospital, Belluno, Italy; ^3^Department of Pediatrics, Ospedale del Ceppo, Pistoia, Italy; ^4^Internal Medicine and Therapeutics Department, Pediatrics and Adolescentology Unit, University of Pavia, Fondazione IRCCS San Matteo, Pavia, Italy; ^5^Operative Unit of Pediatrics and Neonatal Intensive Therapy, Mother and Child Department; ^6^University of Palermo, Palermo, Italy

##### **Correspondence:** Elena Bozzola (elena.bozzola@opbg.net)

**Background:** media use among people is increasing year by year thanks to the advancement of technology.

Also very young children spend times using a social media device (MD) and most of them have their own tablet device. Quite all young children live in home with some type of mobile device and in most of cases there is a television in kid’s bedroom. Moreover, nearly half of them watch television or play videogames before bedtime.

Parents give media devices to children in order to entertain them during meals or in public place to avoid noise. Most of adults think that media device may be useful also to learn foreign languages and to improve their knowledge.

Aim of the study is to analyze the evidences of MD (television, web programs, videos and mobile/interactive technologies) in pre-school children.

**Material and Methods: **We analyzed both beneficial and negative effects of MD use on children’s mental and physical health in order to discuss age-appropriate child’s exposure to media.

**Results: **Scientific evidences have shown that MD use may interfere with: learning and development. On one hand, young children need direct interaction with parents for brain’s development [1,2]. On the other hand, young children can learn words through video if specific conditions are fulfilled: educational apps have been demonstrated to promote learning among preschool children [3] well-being. Evidences suggested that there is an association between tablets usage, weight gain and physical discomfort especially involving neck and shoulders [4,5] sleep. The MD use before sleep correlate to sleep quality [6] sight. The MD use in children correlate to sight difficulties, such as acute acquired comitant exotropia [7] listening. A prolonged exposition of eardrums to intense levels may lead to a dangerous sound immersion without a break period for ears.

**Discussion:** Pediatricians have an important role in educating the children exposure to MD. They should dedicate time during pediatrician controls to inform parents about the beneficial and side effects of MD according to children’s age.

**Conclusions: **In according to the American Academy of Pediatrics we suggest that MD exposure in childhood should be modulated on the basis of clinical evidence and on the age of children, in order to avoid side effects [8].

**References**

1. Schmidt M, Pempek T, Kirkorian H, Lund A, Anderson D. The effects of background television on the toy play behavior of very young children. Child Development 2008;79:1137-1151.

2. Barr R. Memory constraints on infant learning from picture books, television on touchscreens. Child Development Perspectives 2013;7:105-110.

3. Chiong C, Shuler C. Learning: is there an app for that? Investigations of young children’s usage of learning with mobile devices and apps. The Joan Ganz Cooney Center at Sesame Workshop 2010.

4. Hinkley T, Verbestel V, Ahrens W, Lissner L, Molnár D, Moreno LA, Pigeot I, Pohlabeln H, Reisch LA, Russo P, Veidebaum T, Tornaritis M, Williams G, De Henauw S, De Bourdeaudhuij I; IDEFICS Consortium. Early childhood electronic media use as a predictor of poorer well-being: a preospective cohort study. JAMA Pediatrics 2014;168:485-492.

5. Chiang HY, Liu CH. Exploration of the associations of touch-screen tablet computer usage and musculoskeletal discomfort. Work 2016;53:917-925.

6. Cain N, Gradisar M. Electronic media use and sleep in school-aged children and adolescents: a review. sleep medicine 2010;11:735-742.

7. Lee HS, Park SW, Heo H. Acute acquired comitant esotropia related to excessive smartphone use. BMC Ophthalmology 2016; 16:37.

8. American Academy of Pediatrics. Children, Adolescents, and the Media. Pediatrics 2013;132:958-961.

## A12 Tall stature

### Mauro Bozzola^1^, Beatriz Corredor^2^, Chiara Gertosio^1^, Mehul Dattani^3^.

#### ^1^Department of Internal Medicine and Therapeutics, Pediatrics and Adolescent Care Unit, University of Pavia, Pavia, Italy; ^2^Department of Endocrinology, Great Ormond Street Hospital for Children, WC1N 1EH, London, UK; ^3^Section of Genetics and Epigenetics in Health and Disease, Genetics and Genomic Medicine Programme, UCL GOS Institute of Child Health, London, UK

##### **Correspondence:** Mauro Bozzola (mauro.bozzola@unipv.it)

Clinicians generally use the term “tall stature” to define a height more than two standard deviations above the mean for age and sex [1]. In most cases, these subjects present with familial tall stature or a constitutional advance of growth which is diagnosed by excluding other conditions associated with overgrowth. Nevertheless, it is critical to identify situations in which tall stature or an accelerated growth rate indicate an underlying disorder. [2] A careful physical evaluation allows the classification of tall patients into two groups: those with a normal appearance and those with an abnormal appearance including disproportion or dymorphism (Table 1).

In the first case, the growth rate has to be evaluated and, if it is normal for age and sex, the subject may be considered as having familial tall stature or constitutional advance of growth or they may be obese, while if the growth rate is increased, pubertal status and thyroid status should be evaluated. Tall subjects having an abnormal appearance can be divided into proportionate and disproportionate syndromic patients. Before initiating further investigations, the clinician needs to perform both careful physical examination and growth evaluation. To exclude pathological conditions, the causes of pathological tall stature need to be considered, although most children are healthy and generally do not require treatment to cease growth progression. In specific cases, a familial tall stature subject can be treated by inducing puberty early with the aim of inducing early complete fusion of the epiphyses and achievement of final height. Referrals to a pediatric endocrinologist for assessment of a child with tall stature are much less frequent than for short stature. This is because tall stature has a wider social acceptance.

**References:**

1. Meazza C, Gertosio C, Giacchero R, Pagani S, Bozzola M. Tall stature: a difficult diagnosis? Ital J Pediatr. 2017 3;43:66.
2. Corredor B, Dattani M, Gertosio C, Bozzola M. Tall stature: a challenge for clinicians. Current Pediatric Reviews 2018 (in press).

**Table 1 (abstract A12).** Tall stature with normal appearance vs tall stature with abnormal appearance and dymorphisms

Tall stature with normal appearance	Tall stature with abnormal appearance and dymorphisms
Normal growth rate:	Proportionate:
Height SDS-MPH SDS>2 SDS	Sotos
*YES:* Simple obesity	Weaver
Aromatase deficiency,	Fragile X
Oestrogen resistance	Smpson Golabi-Behmel
*NO:* Familial tall stature	
Increased growth rate:	Disproportionate:
*with signs of puberty:*	Marfan
Precocious puberty	Homocystinuria
Pseudoprecocious puberty	Klinefelter
Late onset CAH	Beckwith- Wiedeman
*without signs of puberty:*	Triple X
CAG	
GH excess	
Hyperthyroidism	

## A13 Tuberculosis from Koch to the immigrant child: which evolution?

### Danilo Buonsenso^1,2^, Luigi Cataldi^1,2^

#### ^1^Department of Maternal and Child Health, Fondazione Policlinico Universitario Agostino Gemelli, IRCCS, Roma, Italy; ^2^Gruppo di Studio di Storia della Pediatria, Società Italiana di Pediatria, Italy

##### **Correspondence:** Danilo Buonsenso (danilobuonsenso@gmail.com)

Child tuberculosis (TB) is a silent epidemic involving about one million children each year; of those, nearly one in four die. Children with TB rarely die if they receive standard treatment, but 90% of them worldwide are left untreated. This widespread neglect means the loss of a million children every four years, a not acceptable tragedy if we consider that TB is a preventable, treatable and curable disease. Considering that equal access to medical care is a basic human right, the continuing medical neglect of child TB constitutes a real human rights violation.

This neglect can no longer be excused nor accepted not only by political institutions, but also by medical organizations and by every single doctors, particularly pediatricians.

Ending the child TB epidemic requires local interventions, sensitive to social and cultural context, to reach at-risk children using simple tools for active screening and diagnosis. Even in resource-limited areas, projects like DETECT Child TB are demonstrating that medical professionals diagnose and treat TB in children, even at the community level. Screening households where an adult is diagnosed with TB to see if children have been exposed in the home must become the standard implemented everywhere. Where The Union has piloted this approach in Uganda, 72 percent of at-risk children were able to receive preventive TB treatment, up from less than five percent previously.

In the long run, greater investment in research and development needs to deliver better diagnostics, treatments and an effective vaccine that prevents TB.

In this study, we will discuss the historical developments of these tools and how this have to be included in routine practice. As pediatricians, we cannot forget our public role as guarantors of the health and rights of every child.

## A14 Ophthalmologist and pediatrics

### Luca Buzzonetti, Antonino Romanzo

#### Ophthalmology Department, Bambino Gesù Children’s Hospital, Rome, Italy

##### **Correspondence:** Luca Buzzonetti (robuzze@gmail.com)

Critical is the relationship between ophthalmologist and pediatrics in order to prevent ocular disorders affecting visual development and to plan the right timing for children ophthalmological screening. Amblyopia is a disorder characterized by abnormal processing of visual images in the brain during a critical period of vision development, resulting in a functional reduction of visual acuity. It is associated with conditions that interfere with normal binocular vision, such as strabismus (ocular misalignment), anisometropia (a difference in refractive power between the two eyes), bilateral refractive error, and media opacity (such as cataracts) or other blockage of the visual pathway (such as ptosis or eyelid drooping). We present main pre-scholar screening tests and the more frequent emergency diseases that pediatrics could have to face up in their practice.

Red reflex is a test that can detect potentially life-threatening ocular abnormalities and, despite the high number of false positives, the red reflex test has proven to be a useful, easy to perform and low cost test for the early detection of congenital low vision diseases. The base of the red reflex test is that if the ophthalmoscope light directly placed on the optic axis of the dilated pupil, the pupil area would seem in a uniform light orange (close to red) color. The term “red reflex” is the reflection of the fundus color (the color combination of vascular area and choroid pigments), which returns to the front via the transparent eye path (vitreous, lens, aqueous, and the cornea). Any resentment placed within this path in the eye, partially or completely prevents the reflection, and would appear as a black mark or a shadow.

More frequent pediatric ophthalmological emergency conditions and their appropriate management are presented.

Direct evidence on effectiveness of preschool vision screening for improving visual acuity or other clinical outcomes remains limited and does not adequately address whether screening is more effective than no screening.

Early intervention is critical to prevent treatable causes of vision loss in children, then screening for impaired visual acuity in primary care settings could identify children with vision problems at a critical period of visual development and lead to interventions to improve vision, function, and quality of life. Limited are the evidences in relation to the effectiveness of visual screening tests in pediatric patients.

## A15 Role of editorial office

### Carlo Caffarelli, Claudia De Guido, Francesca Falcinella, Marco Pappalardo

#### Clinica Pediatrica, Dipartimento di Medicina e Chirurgia, Azienda Ospedaliero-Universitaria di Parma, Università di Parma, Parma, Italy

##### **Correspondence:** Carlo Caffarelli (carlo.caffarelli@unipr.it)

The editorial process is a complex work that needs content knowledge, continuous updating, time, organization. Many actors including Authors, Publisher, Editors, Reviewers and Technical Staff are involved [1]. Authors should have followed formatting requirements, manuscript structure, literature citation style and original articles should have obtained approval of Ethical Committee when appropriate. Then, the editorial office must check if the Authors have effectively followed the instructions for Authors of the journal and, when it is necessary, have received the permission to publish material, already appeared in other articles from the Publisher. Editor is a pivot figure in the publication and verifies the content and the process of the script itself. If all conditions of writing are satisfied the Editor receives the manuscript. He sends the paper to reviewers, usually two, to check its quality; otherwise he can take the decision of rejecting the article before reviewing when it is out of scope or not scientifically sound. Technical reviewers to realize statistical or bioinformatics analysis, are sometimes required too. After revision the paper may return to the Authors to make corrections, be rejected or be accepted for publication. The final decision is taken by the Editor. Reviewers are only advisors. When the article has been accepted for the publication, the Technical Staff checks vocabulary and graphic design. So, to warrant a correct and comprehensive publication, a close cooperation among Authors, Editor and Reviewers is needed [2].

**References**

1. De Castro P, Napolitani F, Poltronieri E, Rossi AM. Editor scientifici in Italia: problemi di identità, certificazione e ruoli. Recenti Prog Med. 2016; 107: 567-573.
2. International Committee of Medical Journal Editors Recommendations for the conduct, reporting, editing, and publication of scholarly work in medical Journals. [http://www.icmje.org/icmje-recommendations.pdf]. Last access on 2 August 2018.

## A16 New frontiers in microbiological diagnostics

### Carmelina Calitri, on behalf of Italian Society of Pediatric Infectious Diseases

#### Pediatric Department, Ospedale Cardinal Massaia, Asti, 14100, Italy

##### **Correspondence:** Carmelina Calitri (Carmelina_calitri@libero.it)

Invasive infectious diseases represent threatening conditions in pediatric age: prescription of the most targeted therapy is essential for a good prognosis.

Revision of the available diagnostic methods described in English Literature for pathogen identification.

Polymerase chain reaction methods can identify pathogens in approximately 6 hours directly from biological fluids, some kits disclosing the presence of genes related to antibiotic resistance. Identification is not influenced by the antinfective therapies prescribed, however it is limited to the kit pathogen panel and both false positive (e.g. contamination) and false negative results (e.g. poor pathogen load) may be obtained [1,2,3]. Mass spectrometry is performed on positive cultures: pathogen identification may be accelerated of about 48 hours compared to traditional methods. “Protein spectres” are compared to those encoded in the kit library, so that identification is limited to the species here collected. Methods to identify resistance profiles through mass spectrometry are in progress [4]. Traditional methods as standard identification from cultures and antibiotic susceptibility testing are based on EUCAST guidelines [5].

Pathogen isolation and susceptibility testing on positive cultures remain the gold standard for microbiological diagnosis in infectious diseases. However, mass spectrometry and polymerase chain reaction methods could be adjunctive diagnostic tools for an early diagnosis.

**References**

1**.** Lehmann LE, Hunfeld KP, Emrich T, Haberhausen G, Wissing H, Hoeft A, Stüber F. A multiplex real-time PCR assay for rapid detection and differentiation of 25 bacterial and fungal pathogens from whole blood samples. Med Microbiol Immunol. 2008;197:313-24.

2. Lucignano B, Ranno S, Liesenfeld O, Pizzorno B, Putignani L, Bernaschi P, Menichella D. Multiplex PCR allows rapid and accurate diagnosis of bloodstream infections in newborns and children with suspected sepsis. J Clin Microbiol. 2011;49:2252-8.

3. Biedenbach DJ, Moet GJ, Jones RN. Occurrence and antimicrobial resistance pattern comparisons among bloodstream infection isolates from the SENTRY Antimicrobial Surveillance Program (1997-2002). Diagn Microbiol Infect Dis. 2004;50:59-69.

4. Seng P, Drancourt M, Gouriet F, La Scola B, Fournier PE, Rolain JM, Raoult D. Ongoing revolution in bacteriology: routine identification of bacteria by matrixassisted laser desorption ionization time-of-flight mass spectrometry. Clin Infect Dis. 2009;49:543-51.

5. EUCAST guidelines for detection of resistance mechanisms and specific resistances of clinical and/or epidemiological importance. [http://www.eucast.org/resistance_mechanisms/]. Accessed on 12 July 2018.

## A17 The origin of mankind

### Francesco Callea (francesco.callea@opbg.net)

#### Catholic University of Mwanza, Tanzania

Creation and Evolution theories share two major features: uniqueness and variability of human kind. This approach is new in that bypasses the age-old problem about their conflicting relationship.

In the Bible (Genesis 1 and2) there are at least 4 examples showing how Creation and Evolution theories concerning the origin of mankind share the above two properties: Babel’s Tower, the creation of man, the Great Flood and the creation of Eva.

Evolution theories are based upon the results of paleontologic, anthropologic, ethnic, linguistic and genetic studies. Along Adam and Eva’s traces, the molecular clock and genoma studies, the genealogic tree of human kindship has been reconstructed [1] . The comparative studies have confirmed that the first traces of humans have appeared in the Northern Tanzania and that the migration process known as ”out of Africa” has started from that area of the world for further colonization of all lands of the planet.

Skin color is one of the most conspicuous ways in which humans vary and has been widely used to define human races. Recent studies have definitely proven that the variations in skin color are adaptive and related to the regulation of ultraviolet (UV) radiation penetration in the integument and its direct and indirect effect on good health [2].

The earliest members of the mankind lineage probably had a mostly unpigmented or lightly pigmented integument covered by dark black hairs, similar to that of the modern chimpanzee [3]. The evolution of a naked, darkly pigmented skin occurred early in the evolution of the genus Homo. A dark epidermis protected sweat glands from UV-induced injury, thus insuring the integrity of thermoregulation.

Of greater significance in individual reproductive success was that highly melanized skin protected against UV-induced proteolysis of folate, a metabolite that is essential for normal development of the embryonic neural tube and spermatogenesis.

As hominides migrated out of Africa, varying degrees of depigmentation evolved in order to permit UV-induced synthesis of previtamin D3. Generally speaking the impact of UV-eradiation on skin increases by latitude and decreases by altitude. Recent observations explain the apparent exceptions to the general rule, like the Ituin group in Eskimo population. In general, females require a lighter color of the skin to synthesize more amounts of Vitamin 3, necessary during pregnancy and location [3].

Thus skin coloration in humans is adaptive and labile. Skin pigmentation levels have changed more than once in human evolution (similar to the alternation of glaciation and desertification). Because of that, the skin coloration should not be considered of value in determining phylogenetic relationship among modern human groups.

An ongoing research project supported by two Italian ONLUS Associations (“Friends Raising” and “Alpha-1-antiyrypsin”) is aimed to search in the population of Northern Tanzania the pathogenetic mutations that have been detected in Europe, Asia, America, Oceania and to verify their role ibn the development of cryptogenic cirrhosis and HCC [4] that carry a high incidence in that area of the world. The background of the project is based upon two robust points: the “out of Africa” migration process and upon the finding of pathogenetic mutations in black Americans (Afro-Americans) [5] who have moved from the black Africa to America in more recent years for reason other than the original “out of Africa”. The project sounds as Alpha-1-antitrypsin would return back home.

**References**

1. Cann GR et al. Genealogical tree of human population, Nature 1987 . In Cavalli Sforza LL et al. Homo Sapiens. The story of Human Diversitry. 2013.

2. Diamond J. Geography and skin colour. Nature 2005;435:383-384.

3. Jablonski NG, Chaplin G. The evolution of human skin coloration. J Hum Evol. 2000,39:57-106

4. Callea F, Giovannoni I, Francalanci P, Boldrini R, Faa G, Medicina D, Nobili V, Desmet VJ, Ishak K, Seyama K, Bellacchio E.Mineralization of alpha-1-antitrypsin inclusion bodies in Mmalton alpha-1-antitrypsin deficiency. Orphanet J Rare Dis, 2018;13:79.

5. Pierce JA, Eradio B, Dew TA. Antitrypsin phenotypes in St. Louis. JAMA.1975;2238:609-617.

## A18 How to correctly perform un anthropometric evaluation with percentiles curves and Z-scores

### Angelo Campanozzi (angelo.campanozzi@unifg.it)

#### Clinica Pediatrica, Università di Foggia, Foggia, Italy

The purpose of a nutritional assessment in pediatrics is to document a normal growth and to identify children who need further clinical investigations, since a difficult growth can be the first sign of many diseases. Moreover, a poor nutritional status can modify the therapeutic choices, such as the timing of a surgical operation. Clinical evaluation of the nutritional status is an essential part of the pediatric visit, in both inpatients and outpatients. It does not require particular instruments, but only of a regular systematic approach which is the essential prerequisite for a longitudinal analysis. A child is in good health if her/his growth complies with her/his genetic potential in accordance to normality parameters, namely according to percentiles and z-scores for age and sex. Therefore, the assessment of growth in a child is not only essential to document his/her nutritional status, but it is an essential screening tool for any disease occurring with a reduction of growth-rate.

## A19 Urinary incontinence and enuresis

### Maria L Capitanucci (mluisa.capitanucci@opbg.net)

#### Department of Surgery, Unit of Continence Surgery and Neuro-Urology, Children’s Hospital Bambino Gesu’, Rome, Italy

**Background**: Functional urinary incontinence (UI) and enuresis (EN) are the commonest lower urinary tract symptoms (LUTS) in children and can lead to major distress for the affected children and their parents.

**Materials and Methods**: An extensive search was performed on PubMed and MEDLINE for scientific publications on functional UI and EN in children, with particular regard to International Children’s Continence Society (ICCS)[1] and International Consultation on Incontinence (ICI)[2] recommendations.

**Results**: Based on the ICCS document[1], involuntary urine loss is termed UI. Organic UI (due to anatomic malformation or neurologic disease) is continuous; functional UI (due to disorders of bladder-sphincter function) is intermittent. Up to the 5^th^ year of life, functional UI is regarded as physiological. When functional UI occurs during nighttime is named enuresis. Enuresis may be the only one LUTS (monosymptomatic) or may be associated with daytime LUTS (non-monosymptomatic). Diagnostic evaluation for functional UI aims to exclude organic disorder, to categorize the problem as one of the forms of functional UI and to identify comorbidities (constipation, psychiatric/psychologic disorders). Necessary information can be acquired using non-invasive procedures: detailed medical history, bladder and bowel diary, physical examination and urinalysis. Sonography is used to investigate renal abnormalities, bladder, and rectum. Pathological amounts of postvoiding residual urine (PVR) and relevant thickening of the bladder wall are indications of a bladder voiding disorder. A retrovesically extended rectum indicates constipation. International Consultation on Incontinence (ICI)[2] recommends a specialistic assessment for children with functional UI associated with recurrent/febrile urinary tract infections (UTI), dysfunctional voiding and pathologic PVR. In the other cases, the mainstay of the non specialistic management is urotherapy (educational and rehabilitative procedures); however, some patients will need supportive medication in addition.

**Conclusions**: Urinary incontinence in children is a heterogeneous phenomenon. Functional forms are much more common than organic ones. Diagnosis and therapy of functional UI are based on non-invasive diagnostic evaluation. Urotherapy is the most important therapeutic cornerstone.

**References**

1. Austin PF, Bauer SB, Bower W et al. The standardization of terminology of lower urinary tract function in children and adolescents: update report from the Standardization Committee of the International Children’s Continence Society. Neuro Urol Urodyn. 2016; 35: 471-481

2. Nieman R. Diagnosis and Management of urinary incontinence in childhood. In: Abrams P, Cardozo L, Wagg A, Wein A, editors. Incontinence. 6^Th^ edition. Bristol: ICI-ICS. International Continence Society; 2017. p. 959-1092.

## A20 Phytotherapy in pediatrics: safety and efficacy criteria

### Domenico Careddu^1,2^ (drcareddupediatra@gmail.com)

#### ^1^FIMP (Italian Federation of Pediatricians), Cameri (NO), Italy; ^2^SIMN (Italian Society of Natural Medicine), Cameri (NO), Italy

Phytotherapy has ancient origins but despite this, even today, its use for health purposes is widespread in both adults and children. Many food supplements on the market, contain, among their components, plant extracts. This aspect is particulary relevant in Italy, where the use of food supplements containing plant extracts (botanicals) prevails compared to herbal medicines, unlike what happens in other European Countries. However, there is a marked difference, both from a regulatory point of view and sales to the public, between botanicals and herbal drugs. The main differences, however, concern their clinical use: food supplements can in fact maintain, optimize or support a function, but cannot boast a therapeutic action, that is the sole competence of drugs [1]. It should however be pointed out that plant extracts, whether they are drugs or food supplements, have important biological/pharmacological actions, interactions and even possible adverse side effects [2]. In the pediatric environment, there are plants or their extracts, which cannot be used or have age limits, others that require a dose reduction or caution. [3] It is therefore necessary to have a phytotherapy approach based on criteria and scientific evidence (Monographs, RCTs, clinical studies), exactly as for the medicine that uses synthetic drugs, always having in mind the need to operate according to “science and conscience” and the always valide principle “primum non nocere”. To disseminate these concepts, in 2015, the Italian Federation of Pediatricians (FIMP) published the document “Linee guida di fitoterapia” [4]. The purpose of this report is to provide pediatricians with the skills to be able to advise, according to criteria of safety and effectiveness, the use of plant extracts in child care.

**References**

1. Bilia AR. Herbal medicinal products versus botanical-food supplementes in the European market: state of art and perspectives. Nat Prod Commun. 2015;10:125-31.

2. Heirich M, Barnes J, Gibbons S, Williamson EM. Section 4. In Heirich M, Barnes J, Gibbons S, Williamson EM, editors. Fundamentals of pharmacognosy and phytotherapy. Second Edition. London: Churchill Livingstone;2004. 144-173.

3. Monografie ESCOP. Le Basi Scientifiche dei Prodotti Fitoterapici. Piastrino di Citerna (PG): Planta Medica Edizioni;2006.

4. Linee Guida Fitoterapia FIMP. [https://www.fimp.pro/aree-tematiche/cam-complementary-and-alternative-medicine/118-linee-guida-fitoterapia-fimp]. Last access on 1 August 2018.

## A21 Propanolol: 10 years after

### Maya El Hachem, Claudia Carnevale

#### UOC Dermatology, Bambino Gesù Children’s Hospital IRCSS, Rome, Italy

##### **Correspondence:** Maya El Hachem (may.elhachem@opbg.net)

Infantile hemangiomas (IHs) are the most common tumors of infancy with a prevalence of 2.6-4.5%. IHs are benign vascular tumors. They are characterized by a typical clinical history: they manifest within the first weeks of life, followed by a rapid proliferative phase during the first 5-6 month, a stabilization phase at approximately 8-10 months of age and a successive spontaneous involution over the next 6-7 years. Oral treatment is indicated for ulcerated IHs, IHs at risk for life, those with potential functional or relevant aesthetic sequela [1].

During the last 10 years, treatment of IHs significantly changed after the accidental discovery of efficacy of oral propranolol, a beta-blocking drug, already used in cardiology [2].

The European Consensus of 2015 defines propranolol as the first line therapy for IHs, especially during proliferative phase of IHs, between the second and the fifth month of life. Patients may be treated earlier, in case of obstruction or functional damage, or later in case of delayed referral of the patient [3-4].

The drug showed optimal results in terms of efficacy, safety and tolerability, reserving a secondary or obsolete role to steroids, interferon, vincristine and surgery.

The therapeutic dose ranges from 2 mg/kg/day to 3 mg/kg/day. Children affected by IHs usually need treatment until 12 months of life, but, in case of deep, segmental or laryngeal IHs, may be prolonged until 18-24 months.

Clinical studies, scientific literature and clinical experience of the least 10 years demonstrate that oral propranolol is successfully used in the treatment of IHs.

Recent scientific literature proves that the use of propranolol during the first months of life do not compromise children’s neurological and cognitive development [5-6].

**References**

1. Léauté-Labrèze C, Harper JI, Hoeger PH. Infantile haemangioma. Lancet. 2017 Jul 1;390(10089):85-94.

2. Léauté-Labrèze C, Dumas de la Roque E, Hubiche T, Boralevi F, Thambo JB, Taïeb A. Propranolol for severe hemangiomas of infancy. N Engl J Med. 2008;358:2649-51.

3. Hoeger PH, Harper JI, Baselga E, Bonnet D, Boon LM, Ciofi Degli Atti M, El Hachem M, Oranje AP, Rubin AT, Weibel L, Léauté-Labrèze C. Treatment of infantile haemangiomas: recommendations of a European expert group. Eur J Pediatr. 2015;174:855-65.

4. El Hachem M, F. Gesualdo, A. Diociaiuti, I. Berti, N. Vercellino, V. Boccaletti, I. Neri, G. Porcedda, A. Greco, C. Carnevale, T. Oranges, M. Cutrone, P. Dalmonte. Safety and effectiveness of oral propranolol for infantile hemangiomas started before 5 weeks and after 5 months of age: an Italian multicenter experience. Ital J Pediatr. 2017;19;43:40.

5. Moyakine AV, Kerstjens JM, Spillekom-van Koulil S, van der Vleuten CJ. Propranolol treatment for infantile hemangioma (IH) is not associated with the developmental risk or growth impairment at age 4 years. J Am Acad Dermatol. 2016;75:59-63.

6. Gonzalez-Llorente N, Del Olmo-Benito I, Muñoz-Ollero N, Descalzo MA, García-Doval I, Torrelo A. Study of cognitive function in children treated with propranolol for infantile hemangioma. Pediatr Dermatol. 2017; 34:554-558.

## A22 Depression in adolescents: how intercepting and dealing with it

### Serenella Castronuovo^1^, Giampaolo De Luca^2^

#### ^1^Family pediatrician, Gruppo di Studio Nazionale Adolescenza della SIP, Nettuno (RM), 00048, Italy; ^2^Family pediatrician, Gruppo di Studio Nazionale Adolescenza della SIP, Cosenza, 87100, Italy

##### **Correspondence:** Serenella Castronuovo (serenella.castronuovo@gmail.com)

Depression in adolescents is growing [1].

Doctors, particularly paediatricians, should intercept adolescents at risk as early as possible, becouse: social relations are negatively affected [3], scholastic career completion is at risk [3], only 25-50% of adolescents with depression are recognised and treated, risk of suicide. In Italy suicides represent 12% of deaths in 15-29 age range: second cause among males, third among females [2], depression increases risk of suicide by 10-30 times [3]

Clinic case of a 13 year-old adolescent who was inside depression criteria (Table 1) (Table 2) of DSM5 [4].

Main cause: indirect bullying at school (isolation from the group) [5].

What paediatricians should know: puberty and adolescence are characterised by swinging mood, strong emotions that can disguise a depressive state and make identification difficult; equivalent depressive conditions are: tedium, tiredness, abdominal pains, exhibitionism, hypersomnia, eating disorders and especially irascibility; giving up of social, sport and game activities without apparent reasons is symptomatic; careful anamnesis crucial to investigate familiarity; depressed people tend to recover spontaneously; risk becoming chronic and falls back (40% of cases in 2 years and up to 70% in 5 years [6].

What paediatricians should do: catching early signals to identify difficulties of the adolescents; during health state evaluation at a 12-14 year-old, making simple questions to eventually identify inconveniences, e.g.: How are you? Tell me something about what you do every day? Have you got friends? Carefully observe behaviours, just as a listener, avoiding expressing judgements, following the pace of the patient without imposing pressure and finally avoiding devalueing or trivialising the distress, understanding whether there are specific synptoms (depressed mood, melancholy, weeping, irascibility, loosing of interests or anger crisis); looking for alterations in sleepiness (hypersomnia, insomnia), nutrition (lack of appetite or bulimia nervosa), level of energy (fatigue) or will to undertake new activities; noticing whether melancholy is replaced by rage, as often happens, being the only way adolescents can express their distress.

Latest guidelines published by Pediatrics in 2018 [7,8] recommend training of physicians to identifying depressed adolescents, also using screening tests every year starting from 12.

Pediatrician may adopt CDI-2 screening test (Children’s Depression Inventory, second edition Maria Kovacs 2018) self-report version with 28 Items or brief version with only 12 Items (self-report short) to identify as early as possible adolescents at risk.

**References**

1. Mojtabai R, Olfson M, Han B. National trends in the prevalence and treatment of depression in adolescents and young adults. Pediatrics. 2016,138:e20161878.
2. Contrini E, Battisti L, Ferrari L, ZuccalI M, Fateh-Moghadam P. The impact of the social determinants on life styles in the province of Trento, 2008-13. Not Ist Super Sanità. 2014;27:i-iii.
3. Thapar A, Collishaw S, Potter R, Thapar AK. Managing and preventing depression in adolescents. BMJ 2010; 340:c209.
4. American Psychiatric Association. DSM-5 - Diagnostic and Statistical Manual of Mental Disorders – Fifth Edition. American Psychiatric Publishing, Washington DC, 2013.
5. Pedditzi ML, Lucarelli L. Bullismo e rischio depressivo: una indagine esplorativa in un campione di studenti nella prima adolescenza. *Medico e Bambino pagine elettroniche* 2014; 17. [ https://www.medicoebambino.com/?id=RIC1408_10.html]. Accessed on 12 July 2018.
6. Soldateschi M, Masi G. La depressione nel bambino e nell’adolescente. Salute mentale. 2015:3:120.
7. Zuckerbrot RA, Cheung A, Jensen PS, Stein REK, Laraque D. GLAD-PC STEERING GROUP. Guidelines for adolescent depression in primary care (GLAD-PC): Part I. Practice preparation, identification, assessment, and initial management. Pediatrics. 2018; 141:e20174081.
8. Cheung A, Zuckerbrot RA, Jensen PS, Laraque D, Stein REK. GLAD-PC STEERING GROUP. Guidelines for adolescent depression in primary care (GLAD-PC): Part II. Treatment and ongoing management. Pediatrics. 2018; 141:e20174082.

**Table 1 (abstract A22).** DSM-5, Depressive disorders classification

Coding	DSM-5 – DEPRESSIVE DISORDERS CLASSIFICATION
296.99 (F34.8)	Disruptive Mood Dysregulation Disorder
296.20 ÷ 26 (F32.9 ÷ 6)	Major Depressive Disorder (Single Episode)
296.30 ÷ 36 (F33.9 ÷ 3 + 41-42)	Major Depressive Disorder (Recurrent Episode)
300.4 (F34.1)	Persistent Depressive Disorder (Dysthymia)
625.4 (N94.3)	Premenstrual Dysphoric Disorder
*Various*	Substance/Medication-Induced Depressive Disorder
*Various*	Depressive Disorder Due to Another Medical Condition
311 (F32.8)	Other Specified Depressive Disorder
311 (F32.9)	Unspecified Depressive Disorder

**Table 2 (abstract A22).** DSM-5, Depressive disorders common symptoms

DSM-5 – DEPRESSIVE DISORDERS COMMON SYMPTOMS	
Depressed mood most of the day (e.g., feels sad, empty, hopeless) or irritable mood	
Cognitive or somatic modifications	
Diminished ability to think or concentrate, or indecisiveness, nearly every day	

## A23 Chronic medical conditions: the delicate aspect of transition from pediatric to adult health care

### Graziella S. Cefalo, Giovanna Sironi, Chiara Persico, Alessia Di Benedetto, Marina Crosa, Elisabetta Salvatici, Sabrina Paci, Giuseppe Banderali

#### Pediatric Unit, Department of Health Sciences, University of Milan, San Paolo Hospital, Milano 20142, Italy

##### **Correspondence:** Graziella S. Cefalo (graziella.cefalo@unimi.it)

Advancements in medical treatment and technology have increased the life expectancy of children with special health care needs. The shift from infancy to adulthood represents a delicate moment for the child who is growing up, and for his family. The management of the chronically ill child previously handled by the pediatrician, all-round spokesman of the clinical and organizational problems of the child and his family, is passed on to a greater number of specialists who manage the single health-related issues. This frequently translates in the loss of a holistic approach and with the family being left behind in a new and more fragmented system. Therefore, there is a need to identify collaborative process, tools and resources for all stakeholders involved in the transition process.

The principal problematics to take in consideration while transitioning are: the acquisition of self-consciousness regarding their condition, the capacity to manage their chronic condition easily accessing the assistance services needed and the adequate integration to their social context.

Essential to a successful transition is a correct “education” of the patient, to whom all the issues connected to his condition have been explained, making the patient participative of all the duties previously carried out by his caregivers. At the same time, it is necessary to identify adult specialists adequately trained to care for patients with childhood onset conditions.

Seemingly important is for the pediatrician who is in charge of the child with chronic disease to have an identifiable representative in the multidisciplinary team of the referral center for the specific pathology, for an adequate handing over of the patient. Such take on of responsibility of the patient with identification of his issues, his needs and planning of future follow-up should be discussed by the referral team with a coordinated transfer of care and secure attachment to adult service.

Multiple studies have demonstrated that an unsuccessful transition of care translates in higher rates of scarce compliance to therapy and frequent withdrawal from follow-up, increased hospitalizations for acute complications and inadequate prevention, frequent anxiety and stress disorder related to the loss of a contact person and the fear of a possible relapse or worsening of symptoms. All these problematics have negative consequences for quality of life of the patient and its family. Therefore, there is an increased need for planned programs for transition of youth with special health care needs from the pediatric system to adult health services.

## A24 Deciphering short stature in children

### Francesco Chiarelli (chiarelli@unich.it)

#### Department of Pediatrics, University of Chieti, Chieti, Italy

Short stature is one of the most common reasons for consultation for paediatricians and paediatric endocrinologists. The definition of short stature may vary, but it is commonly defined as a height is 2 standard deviations (SD) or more below the mean for children of that sex and chronologic age (and ideally of the same racial-ethnic group). The first thing first that a paediatrician must rule out is whether the short stature is a variant of normal growth or caused by a disease. When diseases like inflammatory bowel diseases, coeliac disease, cystic fibrosis, thyroid or adrenal diseases are excluded, then the most frequent causes of short stature are familial short stature (FSS), idiopathic short stature (ISS) and constitutional delay of growth and puberty (CDGP).

The diagnosis of the causes underlying short stature sometimes is not easy and availability of growth velocity and growth trajectory is very important for a proper evaluation of a child with short stature. Assessing the severity of the short stature is also important to facilitate decisions about intervention, when appropriate.

In recent years research has given a major contribution to facilitate the deciphering of short stature, with particular reference to discovering of new genes regulating the secretion of GH, IGF-1, ALS, etc. and new molecular mechanisms underlying growth and growth defects (ACAN, PAPPA-2, etc.).

When a diagnosis is made, then the decision to treat or not to treat with GH must be taken; children with GH deficiency, Turner syndrome, SGA, may respond very well to treatment and increase their final height significantly. In other children the outcomes are less advantageous.

Therefore, further research is needed to increase our knowledge in this field and, more importantly, to improve the long term prognosis and final height of short children.

## A25 Vaccination of preterm infants

### Gaetano Chirico (gaechirico@alice.it)

#### Neonatology and Neonatal Intensive Care Unit, Children Hospital, ASST Spedali Civili, Brescia, Italy

The incomparable protective importance and highly favorable cost/effective ratio of immunizations is particularly evident in preterm newborns, who, due to complications of prematurity, are more vulnerable to the harmful consequences of infectious diseases preventable by vaccination.

Preterm infants, especially those with birth weight < 1500 g, may present a reduction of immune response as compared to term infants. Nevertheless, several studies, carried out to evaluate response to vaccination, suggested that they showed a satisfactory response to immunization and developed protective serum antibody levels, including the PCV-7, PCV-10, and PCV-13 pneumococcal vaccine, although slightly reduced as compared to term infants [1-7]. In those at higher risk, an additional booster dose may be warranted in order to ensure a similar response to that of term infants, as in the case of hepatitis B vaccine in infants with birth weight <2000 g. The same may apply to combined vaccines, as the hexavalent that should be administered in accordance to the summary of product characteristics [8].

As for rotavirus vaccination in hospitalized infants, some recommendations suggest to wait until discharge [3], while others consider safe its use in the NICU, with appropriate infection control precautions [8-10].

The occurrence of vaccine-attributable adverse events (such as fever, local inflammatory reaction, prolonged crying, and irritability) are not increased in preterm vaccine recipients. However, extremely low birth weight infants, particularly if immunized before hospital discharge, may show episodes of apnea, bradycardia and desaturation, partly associated to an inflammatory response, that resolve spontaneously in most cases. It is therefore prudent to ensure a 48 hours period of observation and monitoring after administration in these infants [11,12].

In conclusion, according to guidelines issued several years ago and recently reaffirmed, preterm infants should be immunized following chronological age, or when stability has been reached, and should receive full vaccine doses [3].

The measles-mumps-rubella vaccination, to be administered usually after 12 months of age, is also highly recommended [13]; the vaccine could be anticipated in the preterm, in relation to the lower risk of interference by maternal antibodies, which are no longer measurable after six months of age [14].

Although there are no particular contraindications to vaccination in preterm infants, other than those considered for term newborns, it is common to observe a delay of the beginning of vaccinations, as confirmed by both Italian [15, 16]and foreign studies.

It is therefore highly needed a public information campaign, to disseminate the word on the incomparable resource available with vaccinations, and to underline the particular usefulness in some categories of high-risk patients, such as those born preterm.

**References**

1. Esposito S, Serra D, Gualtieri L, Cesati L, Principi N. Vaccines and preterm neonates: why, when, and with what. Early Hum Dev. 2009; 85:S43-5.

2. Gagneur A, Pinquier D, Quach C. Immunization of preterm infants. Hum Vaccin Immunother. 2015; 11:2556-63.

3. Kroger AT, Duchin J, Vázquez M. General Best Practice Guidelines for Immunization. Best Practices Guidance of the Advisory Committee on Immunization Practices (ACIP). [www.cdc.gov/vaccines/hcp/acip-recs/general-recs/downloads/general-recs.pdf]. Accessed on 12 July 2018.

4. Duan K, Guo J, Lei P. Safety and immunogenicity of pneumococcal conjugate vaccine in preterm infants: a meta-analysis. Indian J Pediatr. 2017;84:101-110.

5. Kent A, Ladhani SN, Andrews NJ, Scorrer T, Pollard AJ, Clarke P, Hughes SM, Heal C, Menson E, Chang J, Satodia P, Collinson AC, Faust SN, Goldblatt D, Miller E, Heath PT; PUNS Study Group. Schedules for pneumococcal vaccination of preterm infants: An RCT. Pediatrics. 2016;138.

6. Omeñaca F, Merino JM, Tejedor JC, Constantopoulos A, Papaevangelou V, Kafetzis D, Tsirka A, Athanassiadou F, Anagnostakou M, François N, Borys D, Schuerman L. Immunization of preterm infants with 10-valent pneumococcal conjugate vaccine. Pediatrics. 2011;128:e290-8.

7. Martinón-Torres F, Czajka H, Center KJ, Wysocki J, Majda-Stanislawska E, Omeñaca F, Bernaola Iturbe E, Blazquez Gamero D, Concheiro-Guisán A, Gimenez-Sanchez F, Szenborn L, Giardina PC, Patterson S, Gruber WC, Scott DA, Gurtman A. 13-valent pneumococcal conjugate vaccine (PCV13) in preterm versus term infants. Pediatrics. 2015;135:e876-86.

8. Omeñaca F, Vázquez L, Garcia-Corbeira P, Mesaros N, Hanssens L, Dolhain J, Puente Gómez I, Liese J, Knuf M. Immunization of preterm infants with GSK's hexavalent combined diphtheria-tetanus-acellular pertussis-hepatitis B-inactivated poliovirus-Haemophilus influenzae type b conjugate vaccine: A review of safety and immunogenicity. Vaccine. 2018;36:986-996.

9. Hofstetter AM, Lacombe K, Klein EJ, Jones C, Strelitz B, Jacobson E, Ranade D, Ward ML, Mijatovic-Rustempasic S, Evans D, Wikswo M, Bowen MD, Parashar UD, Payne DC, Englund JA. Risk of rotavirus nosocomial spread after inpatient pentavalent rotavirus vaccination. Pediatrics. 2018;141.

10. Esposito S, Pugni L, Mosca F, Principi N. Rotarix® and RotaTeq® administration to preterm infants in the neonatal intensive care unit: Review of available evidence. Vaccine. 2017.

11. Montague EC, Hilinski JA, Williams HO, McCracken CE, Giannopoulos HT, Piazza AJ. Respiratory decompensation and immunization of preterm infants. Pediatrics. 2016;137

12. Ben Jmaa W, Hernández AI, Sutherland MR, Cloutier A, Germain N, Lachance C, Martin B, Lebel MH, Pladys P, Nuyt AM. Cardio-respiratory events and inflammatory response after primary immunization in preterm infants < 32 weeks gestational age: a randomized controlled study. Pediatr Infect Dis J. 2017;36:988-994.

13. Ferreira CSM, Perin MCAA, Moraes-Pinto MI, Simão-Gurge RM, Goulart AL, Weckx LY, Dos Santos AMN. Humoral immune response to measles and varicella vaccination in former very low birth weight preterm infants. Braz J Infect Dis. 2018;22:41-46.

14. Ichikawa T, Tsuji A, Fujino M, Kusano R, Sugiyama R, Oomori S, Mori K, Maeyama K, Nakayama T. Effect of early measles vaccination (AIK-C strain) for preterm infants. Pediatr Int. 2013;55:163-8.

15. Tozzi AE, Piga S, Corchia C, Di Lallo D, Carnielli V, Chiandotto V, Fertz MC, Miniaci S, Rusconi F, Cuttini M. Timeliness of routine immunization in a population-based Italian cohort of very preterm infants: results of the ACTION follow-up project. Vaccine. 2014;32:793-9.

16. Laforgia N, Mauro AD, Bianchi FP, Mauro FD, Zizzi A, Capozza M, Intini S, Gallone MS, Tafuri S. Are pre-terms born timely and right immunized? Results of an Italian cohort study. Hum Vaccin Immunother. 2018; 19:1-5.

## A26 Organizational Models

### Michele Conversano, Carmela Russo, Tatiana Battista, Giovanni Caputi, Francesco Desiante, Augusto Giorgino, Rosita Cipriani

#### Dipartimento di Prevenzione ASL TA, Taranto, Italy

##### **Correspondence:** Michele Conversano (dip.conversano@libero.it)

The effect of media pressure has determined, in recent years, a lower interest of the population to immunization programmes, increasing the risk of serious consequences for health. The current National Vaccination Prevention Plan 2017-2019 develops in continuity to the previous one, sharing the general objective of harmonizing the prevention strategies actually carried out in Italy, in order to guarantee the full benefits of vaccination to the whole population.

A series of strategies can be carried out in order to create organizational models that aim to increase vaccination coverage. Among these, especially with regards to the developmental age and adolescence, there are health education interventions and the administration of vaccinations in alternative settings. The School, for example, represents the ideal setting for the development of such actions [1]. In addition, internal communication among the various figures involved in the vaccination world (Pediatricians and Immunization Services Operators) is the basis of an effective system of cooperation that would lead to an improvement in coverage.

In the Health Local Unit (ASL) of Taranto, the integration of health promotion and vaccination programmes seems to be a sustainable solution: the comparative assessment of anti-HPV coverage strategies, suggests that school vaccination has resulted in significantly better outcomes than outpatient clinic one, for all the groups considered (overall 72.3% vs 55.6%) [2]. Similarly, the organization of joint assessment meetings of vaccination coverage for a single pediatrician, led to an average increase in vaccination coverage of about 10%.

Building a cooperation system is necessary in order to achieve ambitious goals. The institutional reinforcement between public health and the education system, as well as a multidisciplinary collaboration approach in the vaccination field, are two excellent examples of how these strategies are crucial for achieving ideal coverage [3].

**References**

1. Vandelaer J, Olaniran M. Using a school-based approach to deliver immunization – A global update. Vaccine. 2015;33:719-25.
2. Desiante F, Russo C, Giorgino A, Caputi G, Battista T, Cipriani R, Conversano M. Universal proposal strategies of anti-HPV vaccination for adolescents: comparative analysis between school-based and clinic immunization programs. J Prev Med Hyg. 2017;58: E225-E230.
3. Paul P, Fabio A. Literature review of HPV vaccine delivery strategies: considerations for school- and non-school based immunization program. Vaccine 2014; 32: 320-6.

## A27 Child physical abuse and shaken baby syndrome/abusive head trauma: clinical features

### Elena Coppo^1^, Silvia Pieretti^1^, Sara Racalbuto^1^, Federica Giannotta^2^, Antonio F Urbino^3^

#### ^1^Ambulatorio Bambi, Department of Pediatric Emergency, Ospedale Infantile Regina Margherita, Città della salute e della Scienza, Torino, Italy; ^2^NGO Foundation Terre des Hommes, Milan, Italy; ^3^Department of Pediatric Emergency, Ospedale Infantile Regina Margherita, Città della salute e della Scienza, Torino, Italy

##### **Correspondence:** Elena Coppo (ecoppo@cittadellasalute.to.it)

The physical abuse is defined as the intentional use of physical force against a child that results in—or has a high likelihood of resulting in—harm for the child's health, survival, development, or dignity. Much physical violence against children in the home is inflicted with the object of punishing [1].

Depending on the type of force involved, specific injury patterns are produced on the body of the child, the morphology and localization of which are forensically relevant [2].

Among the diverse lesions that it can be found in maltreated children, same are peculiar and may help to differentiate these forms from accidental injuries:

- Localization: The localization of injuries can contribute decisively to differentiation between accidental origin and abuse-related causes (Figure 1a, b).

- Patterned bruising/injuries: Because of their morphology patterned injuries allow for conclusions as to the weapon used. “Tramline” bruises are a form of child abuse injury that is repeatedly observed; these represent a classic example of a patterned injury [3]**.**

- Bite injuries: show a typical pattern since they reproduce the imprint of the teeth/dentition that caused them [4]**.**

- Repeated injuries: The term repeated injuries refers to the coexistence of injuries of different ages [5]**.**

- Thermal heat injuries: differentiated as either scalds or burns. The severity of the resulting thermal lesion is the product of temperature and exposure time [6-7].

Regarding the brain injuries, Shaken baby Syndrome / Abusive Head Trauma is one of the leading causes of death and disability in infant and young children [8]. It is difficult to estimate its incidence because not all the abused children reach the medical system [9]. For these reasons, long since the scientific community is working hard to identify certain data in order to reach accurate diagnosis of AHT.

Several studies have demonstrated programs are effective in reducing the incidence of the syndrome. Starting from a national network of centres of excellence equipped with a protection team at five main teaching third –level hospitals, coordinated by the no profit organization *Terre des Hommes*, the prevention strategy, the target population and the main message were defined, according to the evidence derived from the international literature review combined with the expert in-the-field experiences. A prevention TV video spot and few synthetic parents-friendly information was provided in the first Italian dedicated web-site [www.nonscuoterlo.it]. This campaign represent an important step towards the prevention of SBS/AHT.

**References**

1. Krug EG, Dahlberg LL, Mercy JA, Zwi A, Lozano R, editors. World report on violence and health. Geneva: World Health Organization; 2002.
2. Michael Tsokos Diagnostic criteria for cutaneous injuries in child abuse: classification, findings, and interpretation Forensic Sci Med Pathol. 2015; 11:235–242.
3. Swerdlin A, Berkowitz C, Craft N. Cutaneous signs of child abuse. J Am Acad Dermatol. 2007;57:371–92
4. Vale GL. Dentistry, bite marks and the investigation of crime. J Calif Dent Assoc. 1996;24:29–4.
5. Nuzzolese E, Di Vella G. The development of a colorimetric scale as a visual aid for the bruise age determination of bite marks and blunt trauma. J Forensic Odontostomatol. 2012;30:1–6.
6. Ellis P. Cutaneous findings in children. In: Collins KA, ByardRW, editors. Forensic pathology of infancy and childhood. NewYork: Springer; 2014. p. 243–65.
7. Faller-Marquardt M, Pollak S, Schmidt U. Cigarette burns in forensic medicine. Forensic Sci Int. 2008;176:200–8.
8. Christian CW, Block R. Abusive head trauma in infants and children, Pediatrics 123.5 2009:1409-1411.
9. Sieswerda-Hoogendoorn T, Boos S, Spivack B, Bilo RA, Van Rijn RR. Educational paper: abusive head trauma part I. Clinical aspects. EurJ Pediatr.2012;171:415-23.

**Fig. 1 (abstract A27).**
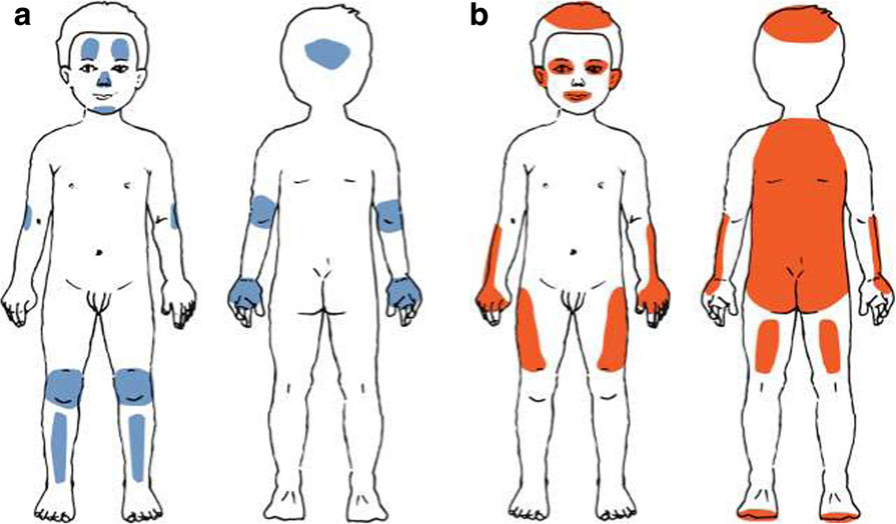
( from Tsokos) Injury localization on a child. a Localizations typical of accidental falling. b Localizations typical of abuse

## A28 Ambulatory blood pressure monitoring in children

### Ciro Corrado (cirocorrado@yahoo.it)

#### Nefrologia Pediatrica ISMEP Palermo, Palermo, Italy

In 2008 were published the first recommendations for the use and interpretation of 24-h ambulatory blood pressure monitoring (ABPM) in children [1]. In the American guideline for screening and Management of high blood pressure, published in 2017, the rule and importance of ABPM in children was emphasized [2]. ABPM is more accurate for diagnosis of Hypertension (HTN) than office–measured blood pressure (BP), is more reproducible than home BP and more predictive of adult BP than office BP. In addition ABPM is superior to office BP to identify patients at the highest risk for target organ damage. ABPM values correlates with left ventricular hypertrophy and carotid intima media thickness, known cardiovascular risk factor. Data provided by ABPM consist of the mean of systolic and diastolic blood pressure values recorded during 24 h and, separately, during daytime and nighttime. The suggested frequency of the measurements is 3–4 per hour during daytime and 2–3 per hour during the night. At least 1 or 2 valid readings should be obtained per hour to consider an ABPM interpretable. According to Guidelines ABPM is useful to confirm HTN in children with office elevate BP values and permit to identify white coat HYT (elevated office BP, normal BP on ABPM) or masked HYT (normal office BP, elevated BP on ABPM). ABPM make it possible to verify the efficacy of therapy, especially when clinic or home BP measurements indicate insufficient BP response. Finally it is useful for the assessment of BP variability, the circadian BP decline from day to night called “dipping” should be > 10% and BP load. BP load excess of 25% is considered abnormal. The American Guidelines recommend routine use of ABPM in children with high-risk conditions: secondary HYT, chronic kidney disease (CKD), diabetes, solid-organ transplant, obesity, obstructive sleep apnea syndrome and in some genetic syndrome (neurofibromatosis, Williams). As we reported in a recent publication [3] however, some critical points concerning its use. In particular, the lack of solid reference values for normal subjects within an age range of 5–16 years and the use of appropriate cuff size and validated monitors. In conclusion in the last years the use of ABPM is increasing. ABPM was recognized useful to identify and classify children with HTN, but we need more normative ABPM data across sex, race and age.

**References**

1. Flynn J, Daniels SR, Hayman LL, Maahs DM, McCrindle BW, Mitsnefes M, Zachariah JP, Urbina EM; American Heart Association Atherosclerosis, Hypertension and Obesity in Youth Committee of the Council on Cardiovascular Disease in the Young. Update: ambulatory blood pressure monitoring in children and adolescents: a scientific statement from the American Heart Association.. Hypertension. 2014; 63:1116-1135.

2. Flynn J, Kaelber DC, Baker-Smith CM, et al; SUBCOMMITTEE ON SCREENING AND MANAGEMENT OF HIGH BLOOD PRESSURE IN CHILDREN. Clinical Practice Guideline for Screening and Management of High Blood Pressure in Children and Adolescents. Pediatrics. 2017; 140: e20171904.

3. Strambi M, Giussani M, Ambruzzi MA, Brambilla P, Corrado C, Giordano U, Maffeis C, Maringhin S, Matteucci MC, Menghetti E, Salice P, Schena F, Strisciuglio P, Valerio G, Viazzi F, Virdis R, Genovesi S. Novelty in hypertension in children and adolescents: focus on hypertension during the first year of life, use and interpretation of ambulatory blood pressure monitoring, role of physical activity in prevention and treatment, simple carbohydrates and uric acid as risk factors. Ital J Pediatr. 2016;42:69.

## A29 Network treatment of childhood obesity: family-school-healthcare systems

### Vita Cupertino^1^, Rita Tanas^2^, Paola Bartoletti^3^, Maria Marsella^4^, Giampaolo De Luca^5^

#### ^1^Community pediatrician, SIP Adolescent Study Group, ASP Cosenza, Italy; ^2^Pediatric Endocrinologist, SIP Adolescent Study Group, Ferrara, Italy; ^3^M.D., Ferrara, Italy; ^4^Pediatric Unit, S. Giuseppe Moscati Hospital, Avellino, Italy; ^5^Family pediatrician, SIP Adolescent Study Group, Cosenza, Italy

##### **Correspondence:** Vita Cupertino (vitacupertino@gmail.com)

Treatment of pediatric/adolescent obesity is of interest for the entire community in terms of multifactoriality and comorbidity. All isolated strategies result less or at all efficacious [1-2].

The child interacts with the environment. Besides family, other microsystems play a role in his development: school, work, church, recreation and sports [3].

Family is the main actor of changes, at least until age 10-12 years, but it is not enough to work only with it during the adolescence. The adolescent should be sustained in his maturation and need of autonomy, ensuring support for motivation, self-esteem and self-efficacy [4].

School (peers, teachers and the entire system) is decisive for relationships, and, therefore, it is a privileged place for implementation of prevention and treatment.

In healthcare a network of services is necessary. Different professionals should be involved with shared training and a *life-course* approach. Professionals should be trained to work on different levels with a shared method and to cooperate in teams. Food and sport lifestyles should be approached with therapeutic education and motivational interviews [2,5].

In the first healthcare level the primary care pediatrician represents the main reference for child/adolescent with obesity and his family. Besides prevention and identification of children who should undergone treatment, his task is to motivate the family towards treatment and support them in time, act as a mediator, participate in decisions when a superior level intervention is required [6].

The second level should receive secondary, severe, already complicated or unresponsive patients [7]. It should be organized by health districts and requires a multidisciplinary team, which includes a pediatrician, a dietitian and a psychologist with documented experience. The role of the team is to define the patient’s clinical picture and activate a personalized multidisciplinary therapeutic intervention.

If severe comorbidities persist, patient should be sent to third level care in specialized centers for the evaluation and treatment of comorbidities on a multidisciplinary basis, including a possible surgical approach [5].

The main obstacles to success of this model, that currently needs to be created or completed in most Italian regions, are the persistence of prejudice on obesity as an exclusive personal responsibility, the failure to recognize obesity as a chronic disease and the lack of coordination among healthcare levels, especially between primary care and multidisciplinary teams. Coordination must be created, maintained and adapted to family’s and patient’s needs. [8]

**References**

1. Tanas R, Lera R, Caggese G. Proposta di modello di cure integrate secondo i canoni dell’ educazione terapeutica per il bambino e l’adolescente con sovrappeso e obesità. Area Pediatrica 2014;15:33-42.
2. Dietz WH, Baur LA, Hall K, Puhl RM, Taveras EM, Uauy R, Kopelman P. Management of obesity: improvement of health-care training and systems for prevention and care. Lancet. 2015 20;385:2521-33.
3. Bronfenbrenner, U., Morris PA. The ecology of human developmental processes. In: Damon, W. Eisenberg, N., editors. The handbook of child psychology. 3. New York: John Wiley & Sons; 1988.p. 993-1027.
4. Boutelle KN, Rhee KE, Liang J, Braden A, Douglas J, Strong D, et al. Effect of attendance of the child on body weight, energy intake, and physical activity in childhood obesity treatment: a randomized clinical trial. JAMA Pediatr. 2017;171:622-8.
5. Consensus SIP – SIEDP su diagnosi, trattamento e prevenzione dell’obesità in età pediatrica 2017. [https://docs.sip.it/Consensus_Obesita_2017.pdf]. Accessed on 12 July 2018.
6. Daniels SR, Hassink SG, Committee in Nutrition. The role of the pediatrician in primary prevention of obesity. Pediatrics. 2015;136:e275-92.
7. Rudolf MC, Krom AJ, Cole TJ. How good are BMI charts for monitoring children's attempts at obesity reduction? Arch Dis Child. 2012;97:418-22.
8. Gruppo di lavoro regionale “Prevenzione dell’obesità”. Contributi n.76/2013: Modello regionale di presa in carico del bambino obeso e sovrappeso. Approvato dalla Giunta della Regione Emilia Romagna il 17 Giugno 2013. Progr.Num. 780/2013. [http://www.saluter.it/documentazione/rapporti/contributi/Contributi_76_2013.pdf/view]. Accessed on 12 July 2018.

## A30 Neurological and behavioral assessment in children with genetic syndrome

### Stefano D’Arrigo, Valeria Tessarollo, Chiara Pantaleoni

#### Developmental Neurology Department, IRCCS Fondazione Istituto Neurologico “C. Besta”, Milan, Italy

##### **Correspondence:** Stefano D’Arrigo (darrigo@istituto-besta.it)

Genetic syndromes are complex conditions characterised by the association of congenital abnormalities with dysmorphisms, and in which staturo-ponderal growth problems, psychomotor delay, intellectual disability (ID) and behavioural disorders are frequent.

Patients presenting these characteristics are often referred to specialist paediatric neurology: clearly, those operating in such settings need to possess specific diagnostic expertise, not only in order to ensure targeted therapeutic and rehabilitative interventions , but also to be able to inform families as to the risk of recurrence. [1]

Diagnosing these syndromes means following a rigorous procedure in which the first step is collection of a detailed history of the family and of the patient himself: pre-natal, neonatal, physiological and pathological. This is followed by clinical investigation which involves primary neurological examination, including exploration of intellectual and behavioral aspects.

Neurologic exam allow to identify neurological signs expression of involvement of central nervous system (pyramidal, extra-pyramidal or cerebellar) or peripheral nervous system. Assessment of cognitive functioning is another important step for identification and characterization of ID that often complicates a genetic syndrome.

ID is a condition characterized by an intellectual functioning significantly below the mean, with concomitant deficiencies or impairments in adaptive functioning developing before 18 years of age. It is encountered in 3% of the population. In early infancy, and the first five years of life in particular, the term developmental delay defines a clinical condition coinciding with a performance at least 2 standard deviations below the mean for chronological age in at least two of the following areas: global and fine motor control, language, cognition, personal/social, and activities of daily living, estimated to affect 5 to 10% of children. [2]

The severity of ID can be classified on the basis of the intelligence quotient (IQ) that emerges on administering standardized tests. Values below 70 (i.e. 2 standard deviations below the mean) identify individuals suffering from ID, which can be distinguished as mild (IQ 50-70); moderate (IQ 30-49); severe (IQ 20-29); or profound (IQ <20). Cases of mild ID form the largest group, accounting for 85% of all cases of ID.

Finally an assessment of behavioral phenotype is necessary as a clinical observation of child’s behavior during natural interaction with parent or examiner, in a comfortable setting, in order to collect information about relation, communication, affective and motor modulation. In some cases it’s possible to use also tests for specific aspects of behavior. [3]

**References**

1. Moeschler JB, Shevell M. Committee on genetics. Comprehensive evaluation of the child with intellectual disability or global developmental delays. Pediatrics. 2014;134:e903-918.
2. Michelson DJ, Shevell MI, Sherr EH, Moeschler JB, Gropman AL, Ashwal S. Evidence report: genetic and metabolic testing on children with global developmental delay: report of the quality standards subcommittee of the American Academy of Neurology and the Practice Committee of the Child Neurology Society. Neurology. 2011; 77: 1629-1635.
3. Srivastava AK, Schwartz CE. Intellectual disability and autism spectrum disorders: causal genes and molecular mechanisms. Neurosci Biobehav Rev. 2014; 46: 161-174.

## A31 Nutritional scores in clinical practice: validity and use

### Enza D’Auria, Alessandra Bosetti^,^ Erica Pendezza, Gian Vincenzo Zuccotti

#### Department of Pediatrics, Ospedale dei Bambini Vittore Buzzi, University of Milan, Milano, Italy

##### **Correspondence:** Enza D’Auria (dauria.e@email.it)

Actually, the double burden of malnutrition is characterized by the coexistence of undernutrition (stunting, wasting, vitamin and mineral deficiency) along with overweight, obesity or diet-related NCDs. To define the nutritional problem of a targeted population, it is necessary to measure its nutritional status**.** Nutritional status assessments enable to determine whether the individual is well nourished or undernourished. Nutritional status assessments of individuals make use of measurable criteria. These criteria reflect physical, physiological and biochemical changes as a result of inadequate food intake (quality and quantity) and diseases.

Nutritional status can be assessed through anthropometric measurements, clinical examination and biochemical testing [1]. Among the evaluation measures, body mass index (BMI) is the most frequently used to characterize the nutritional status of young people and adults. The World Health Organization (WHO) and the International Obesity Task Force (IOTF) developed cut-offs to classify the nutritional status based on BMI, considering age and sex [2, 3].

Different nutrition screening tools are available for use in children of different ages in different care settings [4-7].

The classification of malnutrition risk of the assessed children by the different tools shows a substantial variation in the different tools [8]. Therefore, screening tool selection requires careful consideration of a wide range of issues, such as the purpose of the tool, its reliability and validity and practical issues associated with its implementation.

**References**

1. Puntis JWL. Clinical evaluation and anthropometry. In: Koletzko B, et al, editors. Pediatric Nutrition in Practice. World Rev Nutr Diet. Basel, Karger, 2015; 113, 6–13.

2. De Onis M, Onyango AW, Borghi E, Siyam A, Nishida C, Siekmann J. Development of a WHO growth reference for school-aged children and adolescents. Bull World Health Organ 2007;85:660-7.

3. Cole TJ, Lobstein T. Extended international (IOTF) body mass index cut-offs for thinness, overweight and obesity. Pediatr Obes. 2012;7:284-94.

4. McCarthy HMH, McNulty H, Dixon M, Eaton‐Evans M.J. Screening for nutrition risk in children: the validation of a new tool. J Hu. Nutr Diet. 2008;21, 395–396.

5. Sermet-Gaudelus I, Poisson-Salomon AS, Colomb V, Brusset MC, Mosser F, Berrier F, Ricour C. Simple pediatric nutritional risk score to identify children at risk of malnutrition. Am J Clin. Nutr. 2000;72, 64–70.

6. Hulst JM, Zwart H, Hop WC, Joosten KF. Dutch national survey to test the STRONGkids nutritional risk screening tool in hospitalized children. Clin Nutr. 2010;29, 106–111.

7. Gerasimidis K, Keane O, Macleod I, Flynn DM, Wright CM. A four‐stage evaluation of the Paediatric Yorkhill Malnutrition Score in a tertiary paediatric hospital and a district general hospital. Br J Nutr. 2010;104, 751–756.

8. Chourdakis M, Hecht C, Gerasimidis K, Joosten KF, Karagiozoglou-Lampoudi T, Koetse HA, Ksiazyk J, Lazea C, Shamir R, Szajewska H, Koletzko B, Hulst JM. Malnutrition risk in hospitalized children: use of 3 screening tools in a large European population. AJCN. 2016;103:1301-10.

## A32 Inequalities at birth

### Mario De Curtis^1^, Silvia Simeoni^2^, Luisa Frova^2^

#### ^1^Dipartimento di Pediatria, Università di Roma La Sapienza, Roma, Italy; ^2^Dipartimento per la produzione statistica, ISTAT, Roma, Italy

##### **Correspondence:** Mario De Curtis (mario.decurtis@uniroma1.it)

All the children of the world should be born equal, but this is not the case: even in Italy, there are striking differences at birth.

One of the most accurate indexes to assess demographic wellbeing and quality of life of a population is neonatal mortality (defined as the number of deaths occurring in the first 28 days of life for every 1000 live births) and infant mortality (number of deaths occurring in the first year of life for every 1000 live births). In the last few years, there has been a significant decrease in infant mortality, even surpassing the rates recorded in the most developed Western countries, but neonatal and infant mortality have not decreased homogeneously.

The most recent data from the Italian Statistic Bureau (ISTAT), referring to the 2015 census, show higher neonatal and infant mortality rates in the South compared to North and Central Regions.

Neonatal mortality for every 1000 live births was: in Italy: 2.0; in North West: 1.8; in North Est: 1.5; in Centre: 2.0; in South: 2.3 and in Islands: 2.5.

Infant mortality was in Italy: 2.6; in North West: 2.1; in North Est 2.0; in Centre: 2.2; in South: 3.3 and in Islands: 3.6.

There are several reasons for this disparity: in addition to the well-known differences in social and economic conditions, a decisive role is played by the inadequate organization of perinatal care.

An additional kind of inequality at birth involves babies born to immigrant women. Today in Italy, the foreigners represent 8% of all Italian population. The same recent data from ISTAT referring to the 2015 census show higher neonatal (3.0 vs. 1.8/1000) and infant (4.5 vs 2.6/1000) mortality rates among foreign children living in Italy compared to Italian children (Italian residents). Among babies born to immigrant women, higher infant mortality was seen in children born to women coming from Central Africa (8.6 /1000).

Social, economic, cultural disadvantages, heavier work conditions with scanty social security benefits, inadequate nutrition, poor hygienic living conditions, delayed and inadequate obstetric care of immigrant women during pregnancy, are all causes of the increase in morbidity and mortality risk in the newborn.

There are also marked differences in infant health care among Italian regions, in terms of neonatal expanded screening, palliative care and health care of babies born to immigrants with irregular judicial status.

There is therefore urgent need for a political and social plan focusing on infancy.

## A33 Genetic syndromes in the history of art

### Matteo Della Monica (matteo.dellamonica@meyer.it)

#### UOC Genetica Medica e di Laboratorio, AORN “A.Cardarelli”, Napoli, Italy

The extraordinary discoveries of Genetics in recent decades have brought about radical consequences and changes not only in the medical field, whose scope is still to be fully assessed in their fullness, but also in the social and cultural sphere. The result is the feeling that "genetic syndromes" and rare diseases are in reality only current conditions, of the modern and contemporary world and closely related to our environmental context.

This approach involves the belief that in the past they could not exist or anyway that a person with a genetic condition could not have the possibility of existence, survival or collocation (human / social / economic) in its context.

If all this is true of some peculiar realities (such as Sparta in ancient Greece) or some historical phases in which ideological degenerations have prevailed (such as Nazism in Germany), careful analysis reveals and unveils an unexpected and spectacular world .

In fact, the genetic conditions have always existed and have always accompanied man on his way since the beginning of humanity. The artists over the centuries have simply, rigorously and copiously, documented them. We also discover how different and multifaceted is the motivation of these representations that, depending on the circumstances, could be the simple "photographic" testimony of an objective fact, as a reporter or a reporter does; or it could be determined by the need to explain events on a religious or mythological basis, or to represent the "whimsical" touch of an artist; or the involuntary description of a morphological variant or finally the deliberate and intentional transfiguration of a morphological variant intended to convey a message, to make an allegory or to express an artistic genre.

What is striking is that traces and testimonies of them can be found in all times, in all cultures and in all latitudes.

Unexpectedly, we discover that the feeling that prevails is not of rejection or exclusion but very often of wonder and acceptance.

## A34 How to avoid hospitalization, which drugs, when suggesting hospitalization

### Maurizio Delvecchio (mdelvecchio75@gmail.com)

#### Mother and Child Department, “Madonna delle Grazie” Hospital, Azienda Sanitaria Locale di Matera, Matera, Italy

Diabetic children and adolescents with satisfactory metabolic control have a similar rate of illness or infections as compared to healthy children. There are few reliable data about intercurrent illness in type 1 diabetes, and overall run in adulthood, but nearly all of them are in agreement with this statement. These patients may have altered immune function, increasing susceptibility to and delaying recovery from infections. Some papers describe also impairment in leukocyte function in young patients with poorly controlled diabetes. Patients and families should be provided with clear information on how to manage diabetes during intercurrent illnesses as well as to reach emergency medical personnel, overall diabetes team telephone contacts. Periodical recall of education on the topic should be done. The most dangerous complications to avoid are dehydration, hyperglycaemia, hypoglycaemia, and ketoacidosis. Ongoing monitoring of blood glucose and of urine or blood ketones should be increased. The latter are to be preferred over urine ketones. Insulin should be never suspended also in the case of hypoglycaemia. Vomiting may be not only a sign of intercurrent illness but also of possible ketoacidosis. Fever usually causes hyperglycaemia requiring more insulin dose. In particular, when blood glucose is increased in absence or in presence of only small amount of ketones the total daily dose should be increased by 5–10% as short or subcutaneous rapid-acting insulin. If blood glucose is increased and moderate or large amount of ketones is detected suggesting a increased risk of ketoacidosis, the total daily dose should be increase by 10-20%. The increased need for insulin may persist for some days after recovery likely due to insulin resistance. On the other hand, illness associated with vomiting and diarrhoea may lower blood glucose increasing the risk of hypoglycaemia. Decreased food intake and poorer absorption with overt diarrhoea may contribute to hypoglycaemia. In this case, the insulin dose often needs to be decreased, but should not be lowered to the extent that ketones are produced. A specialist advice should be obtained when the illness is not clear or last-long, or vomiting and weight loss persist, or persistent hypoglycaemia becomes dangerous, or ketonuria or blood ketones are too high, or neurologic status changes. A special attention should be paid to younger children.

## A35 Introduction to health technology assessment

### Pietro Derrico (pietro.derrico@opbg.net)

#### ^1^Bambino Gesù Children's Hospital, IRCCS, Rome, Italy; ^2^Italian Society of Health Technology Assessment, Rome, Italy

Health Technology Assessment (HTA) is the systematic evaluation of properties, effects, and/or impacts of health-care technology. It may address the direct and intended consequences of technologies as well as their indirect and unintended consequences. Its main purpose is to inform technology-related policy-making in health care. HTA is conducted by interdisciplinary groups using explicit analytical frameworks drawing from a variety of methods [1].

The first response to decision-makers’ questions about the uncontrolled diffusion of costly medical equipment was provided by HTA, that began and developed its activities in the early 1970s [2].

Nowadays, the diffusion of HTA knowledge is being a usual part of healthcare services. it is an important tool that can support policy makers and managers working at different levels of health systems (*micro*, *meso* and *macro*) [3]. This process is made possible thanks to the contribution of professionals with different skills. They aim to analyze the main dimensions relative to innovative health technologies (from scientific and industrial research available on the market) that qualify their adoption (cf. effectiveness, safety, costs, ethical and organizational impact) in health facilities, by using the best available evidence [4].

It is necessary to estimate the needs for both health and healthcare assistance, to prioritize, to start evaluation processes and to promote the dissemination and the knowledge transfer.

In this context, the role of the Italian Health Technology Assessment Society (SIHTA), founded in 2007 as a multidisciplinary and multi-professional scientific society, is crucial. Different stakeholders (people and organizations) involved in the evaluation of health technologies (central and regional institutions for the government of health, scientific societies, universities, industries, patients/citizens and their associations), are involved into the SIHTA (www.sihta.it). Its aim is to measure the value of the innovation effects on patients and on health care system. The main communication tools for the dissemination of HTA topic and knowledge, are the Italian annual meeting, health policy forum, training course, etc.

Ensuring the high quality and robustness of evidences and divulged news are considered the principal purpose of the Italian Society.

**References**

1. HTA glossary. International Network of Agencies for Health Technology Assessment and Health Technology Assessment international (http://www.htaglossary.net/) . Accessed on 13 Jul 2018.
2. World health Organization. Health technology assessment of medical devices. WHO Medical device technical series. 2011.[ http://apps.who.int/medicinedocs/documents/s21560en/s21560en.pdf]. Accessed on 13 July 2018.
3. Goodman CS. Technology Assessment: a tool for technology management and improved patients outcomes. Falls Church (US): The Lewin Group;1998.
4. Banta D, Jonsson E. History of HTA: Introduction. Int J Technol Assess Health Care. 2009;25:599-600.

## A36 From PICO to guidelines: the example of Emilia-Romagna region

### Simona Di Mario^1^, Carlo Gagliotti^2^, Maria Luisa Moro^2^

#### ^1^Primary Care Service, Regional Health Authority of Emilia-Romagna, Bologna, Italy; ^2^Regional Health and Social Agency of Emilia-Romagna, Bologna, Italy

##### **Correspondence:** Simona Di Mario (simona.dimario@regione.emilia-romagna.it)

Clinical practice guidelines should be developed following standards of quality [1]. Clear identification of a set of clinical questions is one of the steps: using “population, intervention, control and outcome” (PICO) makes it easier to identify the correct searching strategy and to develop recommendations. Grading the quality of the evidence retrieved and the strength of the recommendations produced, taking in consideration the balance between benefits, harms, value and preference of the patients and feasibility of the proposed intervention, is essential to produce good quality guidelines [2].

Starting from 2007, the Emilia-Romagna region launched the “Progetto Bambini e Antibiotici” (ProBA project ) to improve appropriate antibiotic use in children: ProBA 1 lasted from 2007 to 2013, ProBA 2 from 2013 and is still ongoing.

The project included the development of two regional guidelines for common paediatric infections: acute otitis media and sore throat. Guidelines were firstly published in 2007 and updated thereafter in 2015 [3,4]. Main recommendations for sore throat treatment were: diagnosis of streptococcal pharyngitis using an algorithm based on Mc Isaac score and rapid diagnostic test (RAD) only for score 3 and 4; antibiotic treatment of RAD positive cases or score 5 children using 50 mg. amoxicillin in two daily doses for 6 days [3].

Other activities also included in the ProBA project were: public informative campaign on appropriate use of antibiotics, dissemination of evidence on common paediatric infections, periodical production and publication of regional reports on antibiotic prescription in primary paediatric care and, finally, individual reports of antibiotic prescriptions available for family paediatricians via website and via app for Android. [5]

Temporal trends of two main indicators of appropriate antibiotic prescriptions based on European experience showed a statistically and clinically significant reduction in total antibiotic prescriptions in paediatric population (0-14 years old) and an increase in the ratio of amoxicillin to amoxicillin-clavulanate acid prescriptions (i.e. ratio of first choice to second choice antibiotics for most common paediatric respiratory infections), thus showing an impact of the ProBA project on appropriate antibiotic prescriptions [6,7][Figure 1].

**References**

11. Institute of Medicine (US) Committee on Standards for Developing Trustworthy Clinical Practice Guidelines; Editors: Robin Graham, Michelle Mancher, Dianne Miller Wolman, Sheldon Greenfield, and Earl Steinberg. Clinical practice guidelines we can trust. Washington (DC): National Academies Press (US); 2011.
12. Guyatt GH, Oxman AD, Vist GE, Kunz R, Falck-Ytter Y, Alonso-Coello P, Schünemann HJ; GRADE Working Group. GRADE: an emerging consensus on rating quality of evidence and strength of recommendations. BMJ. 2008; 336: 924-926.
13. Di Mario S, Gagliotti C, Moro ML. Faringotonsillite in età pediatrica. Dossier 253. 2015. [http://assr.regione.emilia-romagna.it/it/servizi/pubblicazioni/dossier/doss253]. Accessed on 12 July 2018.
14. Di Mario S, Gagliotti C, Moro ML. Otite media acuta in età pediatrica. Dossier 254. 2015. [http://assr.regione.emilia-romagna.it/it/servizi/pubblicazioni/dossier/doss254]. Accessed on 12 July 2018.
15. Emilia-Romagna Regional Health Authority. ProBA informative campaign materials. 2017 [http://salute.regione.emilia-romagna.it/campagne/antibiotici.-e-un-peccato-usarli-male-efficaci-se-necessari-dannosi-se-ne-abusi]. Accessed on 12 July 2017.
16. European Commission Prudent use of antimicrobial agents in human medicine: third report on implementation of the Council recommendation. Brussels: Directorate-General for Health and Food Safety; 2016. [https://ec.europa.eu/health/amr/sites/amr/files/amr_projects_3rd-report-councilrecprudent.pdf]. Accessed on 12 July 2018.
17. Di Mario S, Gagliotti C, Buttazzi R, Cisbani L, Di Girolamo C, Brambilla A, Moro ML; regional working group “Progetto ProBA-Progetto Bambini e Antibiotici-2014”. Observational pre-post study showed that a quality improvement project reduced paediatric antibiotic prescribing rates in primary care. Acta Paediatr. 2018 May 3. doi: 10.1111/apa.14381.

**Fig. 1 (abstract A36).**
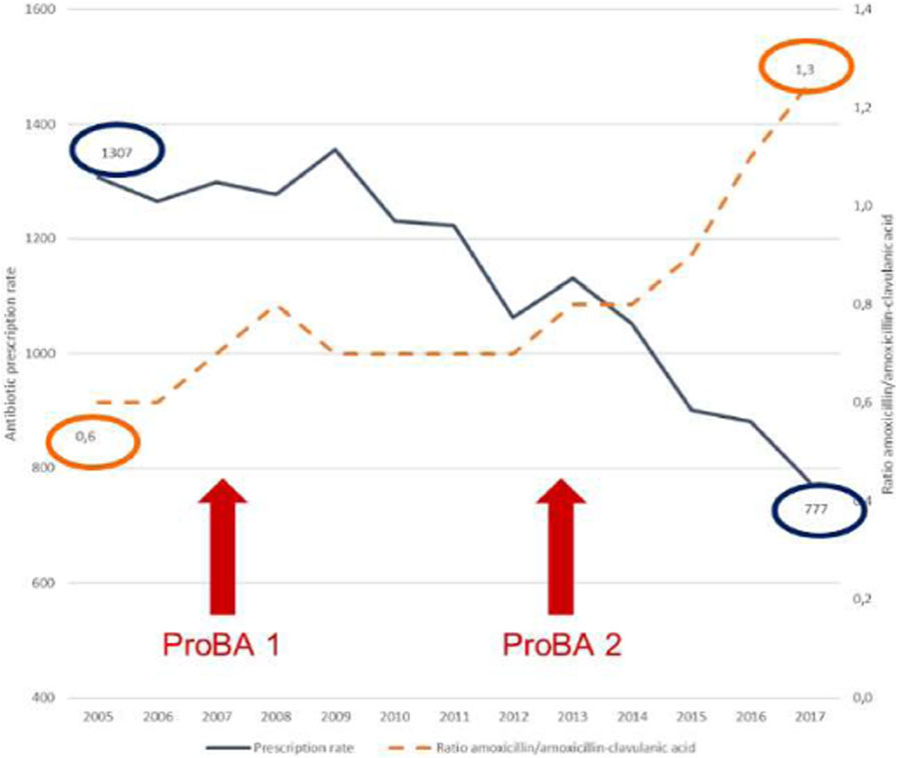
Temporal trend in total antibiotic prescription rate and ratio of amoxicillin to amoxicillin-clavulanic acid in children, 2005-2017

## A37 Elaborating the abuse

### Magda Di Renzo, Elena Vanadia, Federico Bianchi di Castelbianco

#### Istituto di Ortofonologia, Centro di diagnosi e terapia per l’età evolutiva accreditato SSN, Roma, Italy

##### **Correspondence:** Magda Di Renzo (m.direnzo@ortofonologia.it)

Traumatic experiences determine psychic reactions that are almost always proportional to the entity of the event suffered.

What generally occurs, as an immediate response to the trauma, is the division of the affective dimension from the memory of the event. The pain linked to the traumatic experience would, in fact, be intolerable for the individual and therefore the psyche splits itself allowing access to memory only through the sensorial channel in the form of flash-backs that are repeated without allowing a complete memory of the event.

When the traumatic experience, as in the case of abuse, undermines trust in the relationship, it is possible that the defense mechanism of the split is deeper ten preventing any access to memory.

Unlike traumatic events resulting from environmental factors, such as an earthquake, the abuse, as an act of violence perpetrated by a human being, activates more archaic and intense defenses, threatening the child's attachment style and general functioning. In these cases the impact of the traumatic experience can determine important regressions and favor inhibitions of the energy available for growth, triggering behavioral disorders and / or cognitive organization.

The traumatic event of abuse can also reactivate previous experiences of inadequate care, thus determining a more complex psychopathological picture that must be seen as a cumulative effect of previous distortions and current responses. Post-traumatic stress disorder therefore requires special attention in childhood for all the components of development and the environmental dynamics that have accompanied the development of the child, especially if the abuse involves important reference figures within the family unit or school environment.

It often happens that behavioral problems or delays cover unprocessed levels of distress due to the removal of traumas. It is necessary a great competence, as well as a total ability to listen, to frame the origin of the difficulties of the child and go back, sometimes, to traumatic conditions not yet highlighted by the environment.

The elaboration of a trauma such as abuse requires a specific path that allows the reintegration of affective contents to the sensory ones and a reconnection of warm and cold memories to resume the thread of one's autobiographical narrative. Within a psychotherapeutic process, which considers the integration of these aspects central, can also be useful trauma-specific interventions that also use the body dimension for the reconstruction of the story but always within a significant relationship that can give meaning to the events remembered.

## A38 The image of the nursing Madonna

### Raffaele Domenici (r.domenici@usl2.toscana.it)

#### Pediatrics Unit, San Luca Hospital, Department of Mother and Child Health, AV North West Tuscany, Lucca, Italy

In Christian art history, the iconography of Mary is the most widespread from the first centuries after Christ.

Amongst the different types of images, that of Mary breastfeeding or about to breastfeed Jesus stand out for their peculiarity, highlighting both the humanity and divinity of Christ.

In several cases the two characters are represented alone, painting an intimate, tender scene, at the time of breastfeeding. In others, the breastfeeding scene is part of a wider context that includes other characters, as in the works representing the Holy Family, the Flight into Egypt, the Sacred Conversation. In others, the act of breastfeeding elects the Virgin Mary as a mediator between the worshippers and God.

The most ancient known effigy of Mary dates back to the II/III Century and it is in fact a Nursing Madonna located in the Catacomb of Priscilla in Rome.

The official start of this iconography of Mary dates back to the Council of Ephesus (431 AC) which declared the dogma of Mary as the Mother of God and not only of the God-Man Jesus.

In subsequent times this iconography would mostly develop within Byzantine art, in church paintings, where Mary would often be represented frontally, sitting on a throne, with the Baby resting on her knees, and in icons. The latter differentiate in many typologies of images, with increasingly affectionate representation such as that of Mary breastfeeding.

In the early 300, the iconography of the Virgin Mary evolved greatly, especially in Tuscany. The representations, developed at the same time, of pregnancy and breastfeeding convey a desire to humanise the holy sphere with a new conception of Mary, seen as a woman within a family environment.

To the earlier Paleochristian and Byzantine representations, typically solemn and sacral, this period juxtaposes representations of Mary displaying a simple and spontaneous attitude, intimate and very natural in her maternal duties.

The restrictive guidelines of the Council of Trent had great impact on religious representation and on the representation of Nursing Madonna as well. Devotion for the iconography was heartfelt, but it was an expression of the Apocryphal Gospels, in which popular fantasy enhanced many of the narrations of the Canonical Gospels.

In the severe, conservative climate that was established in all religious aspects, including artistic expression, freedom and autonomy were no longer tolerated and the images of the Nursing Madonna were considered inconvenient.

The misunderstanding in understanding the symbolic value of the Nursing Madonna and of her gestures caused the reduction in these representations and in the devotional practices associated to them.

## A39 Neonatal and paediatric arrhythmias: clinical and electrocardiographic aspects

### Fabrizio Drago, Irma Battipaglia

#### Paediatric Cardiology and Cardiac Arrhythmias Unit, Department of Paediatric Cardiology and Cardiac Surgery, Bambino Gesu` Children’s Hospital and Research Institute, Rome, Italy

##### **Correspondence:** Fabrizio Drago (fabrizio.drago@opbg.net)

Progressive changes in heart anatomy and physiology, taking place between birth and adolescence, result in differences between the normal adult pattern and paediatric ECG [1].

Sinus arrhythmia, ectopic atrial rhythm, “wandering pacemaker” and junctional rhythm are examples of normal paediatric rhythm.

In paediatric patients, diagnosis of bradycardia depends on the age [2-3]. In general, it is defined bradycardia, at rest or awake, a heart rate <100 bpm in children up to 3 years old, < 60 bpm in patients 3-9 years old, <50 bpm in patients 9-16 years old and < 40 bpm for patients older than 16 years.

Tachycardia is defined as a sequence of three or more beats at a rate >25% of the sinus rate at the onset of the arrhythmia (usually 120 bpm).

Supraventricular extrabeat: generally idiopathic and clinically silent, it usually disappears during the first year of life; no treatment needed.

Ventricular extrabeat: usually idiopathic, with no symptoms. A structural cardiopathy or more complex arrhythmias must be excluded; in general, they disappear spontaneously.

Paroxystic supraventricular reentry tachycardia (SVT): the most common arrhythmia in children (82%). Often, preschool children can describe palpitations as “precordial pain”. Main reentry tachyarrhythmias are: SVTs due to accessory pathways: atrioventricular re-entry tachycardias (AVRT). A particular type of AVRT is the permanent junctional re-entry tachycardia (PJRT); Re-entry in the AV node: atrioventricular nodal re-entry tachycardia (AVNRT). It is rather rare in paediatric age (13-16% of all SVTs). Nowadays, very safe and effective ablation techniques exist for its treatment (such as cryoablation with 3D mapping system guide) [4-11].

Ventricular tachycardias (VTs): rare in paediatric age (5-10% of all tachyarrhythmias). They can be idiopathic or expression of a structural heart disease, a cardiomyopathy, a myocarditis, cardiac tumours, electrolytes disorders, channelopaties. Secondary forms are more frequent than idiopathic ones.

Atrioventricular block (AVB): first degree AV block is characterized by a prolongation of the AV conduction. Second degree AV block Mobitz 1 consists in a progressive prolongation of the PR interval until a P wave is not followed by a QRS. Second degree AV block Mobitz 2 is an intermittent and sudden block in the AV conduction. In third degree AV block there is a complete interruption of the AV conduction and pacemaker implant can be necessary [12-15]. Third degree AVB can be isolated, usually congenital, or associated to congenital heart disease. In 70-80% of congenital isolated AV block, maternal autoantibodies are present [16-17].

**References**

1. Dickinson DF. The normal ECG in childhood and adolescence. Heart 2005;91:1626–1630.
2. Deal BJ, Wolff GS, Gelband H, editors. Current concepts in diagnosis and management of arrhythmias in infants and children. Armonk, NY: Futura; 1998.
3. Gillette PC, Garson A, editors. Clinical pediatric arrhythmias, 2nd edition. Philadelphia: WB Saunders; 1999.
4. Bauersfeld U, Pfammatter JP, Jaeggi E. Treatment of supraventricular tachycardias in the new millennium-drugs or radiofrequency catheter ablation? Eur J Ped. 2001;160:1-9.
5. Van Hare GF, Javitz H, Carmelli D, Saul JP, Tanel RE, Fischbach PS, et al. Prospective assessment after pediatric cardiac ablation: demographics, medical profiles, and initial outcomes. J Cardiovasc Electrophysiol, 2004; 15:759-770.
6. Drago F, Silvetti MS, Di Pino A, Grutter G, Bevilacqua M, Leibovich S. Exclusion of fluoroscopy during ablation treatment of right accessory pathway in children. J Cardiovasc Electrophysiol. 2002; 13:778-782.
7. Miyazaki A, Blaufox AD, Fairbrother DL, Saul JP. Cryo-ablation for septal tachycardia substrates in pediatric patients: mid-term results. J Am Coll Cardiol. 2005;45:581-8.
8. Drago F, De Santis A, Grutter G, Silvetti MS. Transvenous cryothermal catheter ablation of re-entry circuit located near the atrioventricular junction in pediatric patients: efficacy, safety, and midterm follow-up. J Am Coll Cardiol. 2005;45:1096-103.
9. Drago F, Silvetti MS, De Santis A, Grutter G, Andrew P. Lengthier cryoablation and a bonus cryoapplication is associated with improved efficacy for cryo catheter ablation of supraventricular tachycardias in children. J Interv Card Electrophysiol. 2006;16:191-198.
10. Drago F, Russo MS, Battipaglia I, Grifoni G, Silvetti MS, Remoli R, Pazzano V, Saputo FA, Ciani M. The need for a lengthier cryolesion can predict a worse outcome in 3D cryoablation of AV nodal slow pathway in children. Pacing Clin Electrophysiol. 2016;39:1198-1205.
11. Drago F, Battipaglia I, Russo MS, Remoli R, Pazzano V, Grifoni G, Allegretti G, Silvetti MS. Voltage gradient mapping and electrophysiologically guided cryoablation in children with AVNRT. Europace. 2017;00:1-8.
12. Silvetti MS, Drago F, Grutter G, De Santis A, Di Ciommo V, Ravà L. Twenty years of cardiac pacing in paediatric age: 515 pacemakers and 480 leads implanted in 292 patients. Europace. 2006;8:530-536.
13. Silvetti MS, Drago F, Marcora S, Ravà L. Outcome of single-chamber, ventricular pacemakers with transvenous leads implanted in children. Europace. 2007;9:894-899.
14. Drago F, Silvetti MS, De Santis A, Fazio G, Biancalana G, Grutter G, Rinelli G. Closed loop stimulation improves ejection fraction in pediatric patients with pacemaker and ventricular dysfunction. Pacing Clin Electrophysiol. 2007;30:33-7.
15. Silvetti MS, Di Carlo D, Ammirati A, Placidi S, Di Mambro C, Ravà L, Drago F. Left ventricular pacing in neonates and infants with isolated congenital complete or advanced atrioventricular block: short- and medium-term outcome. Europace. 2015;17:603-10.
16. Michealsson M, Engle M. Congenital complete heart block: an international study of the natural hystory. Cardiovasc Clin. 1972;4:85-101.
17. Jaeggi ET, Hamilton RM, Silverman ED, Zamora SA, Hornberger LK. Outcome of children with fetal, neonatal or childhood diagnosis of isolated congenital atrioventricular block. A single institution experience of 30 years. J Am Coll Cardiol. 2002;39:130-137.

## A40 Variations in the timing of puberty

### Maria F Faienza^1^, Gabriele D’Amato^2^

#### ^1^Department of Biomedical Sciences and Human Oncology, University “A. Moro”, Bari, Italy; ^2^Neonatal Intensive Care Unit, Di Venere Hospital, Bari, Italy.

##### **Correspondence:** Maria F Faienza (mariafelicia.faienza@uniba.it)

Puberty is the results of reactivation of gonadotrophin-releasing hormone (GnRH) secretion. The beginning of puberty involves a shift from a predominantly inhibitory to an excitatory control which results in diurnal activation of pulsatile GnRH release leading to increased luteinizing hormone (LH) pulsatility, the first endocrine manifestation of puberty. The change in pulsatile GnRH release results from activation of excitatory networks operating in the arcuate nucleus of the hypothalamus, with kisspeptin/neurokinin B/dynorphin (KNDy) neurons playing a central role [1]. The timing of puberty varies significantly in the general population and is influenced by both environmental and genetic factors [2,3]. Data from studies within ethnic groups, and between monozygotic compared to dizygotic twins suggest that 50–80% of the variation in pubertal timing is determined by genetic factors [4,5]. There is also strong evidence of environmental effects on the timing of puberty, as well as of secular trends [7,8]. A marked trend towards an earlier age at menarche has been observed over the last 150 years [9]. In many countries the age at menarche has been progressively decreasing at a consistent rate of around 3 years for every hundred years (3.6 months/decade) [9]. European data from the last 50 years have shown variable rates of earlier age at menarche, with both faster and slower rates. This heterogeneity is related to social class, income, urban versus rural background, education and family size [3]. Furthermore, nutritional changes play an important role as reflected by positive correlations between age at puberty onset or age at menarche and body mass index [10]. The effects of endocrine disrupting chemicals (EDC) on pubertal timing have been addressed in a number of studies which indicated that pubertal timing can be affected through exposure during different periods of life. The resulting changes depend on the EDC, the period of exposure and the endpoint chosen for evaluation of puberty [11]. Furthermore, epigenetic mechanisms seem to play a significant role in the neuroendocrine regulation of reproductive axis through a switch from transcriptional inhibition to transcriptional activation [1]. The timing of puberty has been also associated with various adult diseases and linked to the potential development of depression and anxiety, eating disorders, risky sexual activity, and aggressive and antisocial behavior at an early age [12-14]. Family-based and peer-focused preventive interventions may improve parent-child relationships, communication about puberty, and foster healthier peer interactions.

**References**

1. Lomniczi A, Wright H, Ojeda SR. Epigenetic regulation of female puberty. Front Neuroendocrinol. 2015; 36:90-107.

2. Parent AS, Teilmann G, Juul A, Skakkebaek NE, Toppari J, Bourguignon JP. The timing of normal puberty and the age limits of sexual precocity: variations around the world, secular trends, and changes after migration. Endocr Rev. 2003; 24:668-693.

3. Parent AS, Rasier G, Gerard A, Heger S, Roth C, Mastronardi C, et al. Early onset of puberty: tracking genetic and environmental factors. Horm Res. 2005; 64:41-47.

4. Palmert MR, Hirschhorn JN. Genetic approaches to stature, pubertal timing, and other complex traits. Mol Genet Metab. 2003; 80:1–10.

5. Towne B, Czerwinski SA, Demerath EW, Blangero J, Roche AF, Siervogel RM. Heritability of age at menarche in girls from the Fels Longitudinal Study. Am J Phys Anthropol. 2005; 128:210-219.

7. Demerath EW, Towne B, Chumlea WC, Sun SS, Czerwinski SA, Remsberg KE, Siervogel RM. Recent decline in age at menarche: the Fels Longitudinal Study. Am J Hum Biol. 2004; 16:453– 457.

8. Euling SY, Herman-Giddens ME, Lee PA, Selevan SG, Juul A, Sørensen TI, et al. Examination of US puberty- timing data from 1940 to 1994 for secular trends: panel findings. Pediatrics. 2008; 121:S172–S191.

9. Ong KK, Ahmed ML, Dunger DB. Lessons from large population studies on timing and tempo of puberty (secular trends and relation to body size): the European trend. Mol Cell Endocrinol. 2006; 254-255:8-12.

10. Li W, Liu Q, Deng X, Chen Y, Liu S, Story M. Association between obesity and puberty timing: a systematic review and meta-analysis. Int J Environ Res Public Health. 2017;14; E1266.

11. Bourguignon JP, Juul A, Franssen D, Fudvoye J, Pinson A, Parent AS. Contribution of the endocrine perspective in the evaluation of endocrine disrupting chemical effects: the case study of pubertal timing. Horm Res Paediatr. 2016; 86:221-232.

12. Day FR, Elks CE, Murray A, Ong KK, Perry JR. Puberty timing associated with diabetes, cardiovascular disease and also diverse health outcomes in men and women: the UK Biobank study. Sci Rep. 2015; 5:11208.

13. Patton GC, McMorris BJ, Toumbourou JW, Hemphill SA, Donath S, Catalano RF. Puberty and the onset of substance use and abuse. Pediatrics 2004; 14:e300e6.

14. Graber JA, Nichols TR, Brooks-Gunn J. Putting pubertal timing in developmental context: implications for prevention. Dev Psychobiol 2010; 52:254e62.

## A41 The voice of children living in foster care

### Francesca Ianniello, Pietro Ferrara

#### Institute of Pediatrics, Catholic University of Sacred Heart, Rome, Italy

##### **Correspondence:** Francesca Ianniello (francescaianniello@hotmail.com)

**Background**

Children living in foster care belong to a vulnerable child population that is afflicted by a wide range of acute and chronic health conditions requiring multidisciplinary care services. We evaluate the vaccination coverage of children living in a foster care, highlighting the standpoint of social educators working in these settings.

**Material and methods**

Data come from paediatric evaluation of a sample of children in group-homes in Rome, between September 2011 and April 2012 (G1) and these data were compared with a sample of children in group-homes in Rome, from January 2018 (G2).

**Results**

About children in foster care (G1) it was found that 91/112 children (81.2%) had the vaccine coverage for all vaccines that compose the hexavalent (diphtheria, tetanus, pertussis, hepatitis B, poliomyelitis and Haemophilus influenzae type b) vaccine; 88/112 (78.6%) have been vaccinated for measles-mumps-rubella; 10/112 (8.9%) were vaccinated with meningococcal vaccine; 15/112 (13.4%) have been vaccinated with vaccine anti-pneumococcal [1]. In G2 group the preliminary data showed that 20/20 (100%) had the vaccine coverage for all vaccines that compose the hexavalent (diphtheria, tetanus, pertussis, hepatitis B, poliomyelitis and Haemophilus influenzae type b) vaccine; were vaccinated with meningococcal vaccine and with vaccine anti-pneumococcal 20/20 children (100%).

**Discussion and Conclusion**

Children living in residential child care, like foster care, have still serious deficiencies in their overall health and wellbeing, particularly in emotional health and behavior and vaccine coverage. The vaccination status is an important gauge to evaluate the quality of assistance in children in the general population and more than in this vulnerable group of children [2]. The results of this study confirm that immunity surveillance is an important aspect for children in foster care. The full integration of these children in the social and sanitary setting of the welcoming country must became a target for all foster care systems and for all figures who take care of children in group-homes.

The improvement of vaccination coverage in recent years demonstrates the greater attention and sensitivity of the institutions and pediatricians towards these children.

**References**

1. Ferrara P, Fabrizio GC, Romani L, Ianniello F, Valentini P, Alvaro F, Gatto A. Immunization status of children in foster homes: the first Italian data. Minerva Pediatr. 2016;68:36-39.

2. Ferrara P, Romani L, Bottaro G, Ianniello F, Fabrizio GC, Chiaretti A, Alvaro F. The physical and mental health of children in foster care. Iran J Public Health. 2013;42:368-373.

## A42 Farmacoresistent epilepsy (DRE-ILAE)

### Alberto Fois (ftnp@libero.it)

#### Siena, Italy

AKA: Epilepsy not responsive or intractable with antiepileptic drugs (AEDs) or the impossibility to obtain therapeutic results utilizing appropriated trials with two drugs chosen and applied properly.

The percentage of patients with DRE is evaluted to be between 20 and 40 % but is considered 10 % in pediatric patients.

Not to be included in this group are patients with pseudoresistance due to diagnostic ot treatment errors.

The meccanism of DRE can be referred to intrinsic od acquired modifications in the target tissues, to transport defects at the level of hematoencephalic barrier (BBB).

The target of AEDs are the ionic channels, the receptors neurotrasmitters, the carrier proteins and the enzymes involved in farmacometabolism.

The multidrugs carrier meccanism includes some proteins: the P- glycoprotein (Dgp), the resistance to multigrug associated protein (MRP), the breast cancer resistance protein (BCRP).

These proteins are important obstacles for the transit of the drugs to the brain.

A number of hypoteses can be consired to explain the farmacoresistance: loss of efficiency of AEDs, etiology of epilepsy, disease progression or changes in the targets, peculiarities of genetic substrate of the epileptic syndrome.

Genetic of DER: Common genetic variations are SCN1A and SMEI.

Animals models: It is yet non know how and why epilepsy in some patients is intractable while in others with apparent identical seizures, it is possible to obtain a seizure control with the same drugs.

The animal model allow a division between farmaco resistant and farmaco sensibile animals, making possible to imitate mecanism underlying what can be found in human epilepsy.

From this perspective two models look particularly interesting epileptic dogs with spontaneous epilepsy and rats with amigdala epilepsy obtained with kindling.

Some hypotheses can at least in part explain the resistance.

A possibility is the drug transport throug BBB.

Another possibility has something to do with the drug targets because acquired abnormalities in the structure or functions of the tonic channels can produce an insufficient farmacodinamic activity of AEDs in cerebral tissues.

A third hyphotesis concenrs the network system (E.G. An hyppocampal sclerosis can explain a resistance to AED).

A fourth hypothesis regards a genetic variant acting through the proteins with a farmacocinetic of farmacodinamic function on AEDs.

Another possibility is the gravity of the epileptic lesion.

An interaction of different meccanisms acting in the same patient is possible.

Therapy of DRE can utilize differen types of stimulation.

## A43 Insulin-resistance in adolescents: a disease factor

### Adriana Franzese (franzese@unina.it)

#### Università Federico II, Napoli, Italy

Insulin-resistance (IR) is a metabolic and immunologic condition, as well as being the step that comes before a definite state of hyperglycemia. An inflammatory response in immune cells exists in vitro in hyperglycemic milieu, secretion of IL-1b, IL-6, IL-8, MCP-1, and other major cytokines has been demonstrated in both monocytes and macrophages derived from patients with diabetes mellitus 2 and in monocytic cell lines exposed to hyperglycaemia. Furthermore hyperglycaemia induces accumulation of senescent cells that can have a pro-inflammatory phenotype. All this is far to being well definite in a pre-diabetic condition as IR. Anyway, this condition is very common in obese children and is linked with prevention of cardiovascular events and health quality in long term.

I will propone an extended reflection on IR as disease factor, all the more important when referring to the pediatric age.

## A44 Is transition care in congenital heart disease underestimated?

### Maria G Gagliardi, Micol Rebonato

#### DMCCP, Ospedale Bambino Gesu’, Roma, Italy

##### **Correspondence:** Maria G Gagliardi (mgiulia.gagliardi@opbg.net)

Transition in health care for young adults with special health care needs is a dynamic, lifelong process that seeks to meet their individual needs as they move from childhood to adulthood. The goal is to maximize lifelong functioning and potential through the provision of high-quality, developmentally appropriate health care services that continue uninterrupted as the individual moves from adolescence to adulthood. It is patient centered, and its cornerstones are flexibility, responsiveness, continuity, comprehensiveness, and coordination [1].

The key problem in the management of GUCH patients is a lack of understanding the importance of a coordinated transitioning process from pediatric to adult care services. It is acknowledged that cooperation and communication between specialists and settings and a managed transitioning process are paramount.

The outlook for children born with congenital heart disease continues to improve as a result of advances in pediatric cardiac surgery, catheter interventions, medical and perioperative management and imaging techniques. Most patients are now expected to live to adulthood, leading to a significant increase in the population of adults with congenital heart disease. These young people need to be supported as they make the transition into adulthood; they must be encouraged to take responsibility for their own health and to make informed decisions regarding careers and lifestyle [2].

Patients with CHD are at risk of having gaps in congenital heart care that may lead to worse long-term outcomes . Loss to follow up is an important challenge as lapses in adult CHD care may predispose patients to delayed recognition of new cardiac problem. Adolescent survivors of CHD are at risk of substantial cardiac morbidity and mortality in the early to mid-adult years and most require lifelong follow up with a cardiologist with specialized training and expertise.

The benefits of a well‐planned transition include improvements in clinical, educational and social outcomes for young people.

Transition clinics should begin by 12 years of age, with age‐appropriate information, discussion and objectives. The staged approach should allow the patient to feel secure with their new medical team before full transfer to adult services around 16–18 years of age. When successful it should empower them to take control of their own health and provides a platform for optimal disease management throughout their adult life [3].

A poor transition risks alienating young adults from healthcare providers with subsequent poor compliance, loss to follow‐up and substandard medical care.

**References**

1. Hudsmith LE, Thorne SA. Transition of care from pediatric to adult services in cardiology. Arch Dis Child. 2007;92:927–930.

2. Mackie AS. Transition intervention for adolescents with congenital heart disease. JACC 2018;71:16.

3. Gibbs J, Gibbs S, Thorne S, Cumper M, Vernon S, Deanfield J, Tsang V, Haw M, Lake C, Davies R. Adult congenital heart disease: a commissioning guide for services for young people and grown ups with congenital herat disease. London:Department of Health;2006.

## A45 Diagnostic routes in urinary tract malformations in the newborn

### Rossella Galiano^1^, Pasquale Novellino^2^

#### ^1^SSD di Neonatologia POLT ASP, Catanzaro, Italy; ^2^Terapia Intensiva Neonatale, Azienda Ospedaliera Pugliese-Ciaccio, Catanzaro, Italy

##### **Correspondence:** Rossella Galiano (rgaliano@libero.it)

Congenital anomalies of the kidney and urinary tract (CAKUT) are common (about 30% of the malformations)[1]and severe (the main cause of chronic kidney disease (CKD) in the pediatric age) and a predisposing factor for hypertension and cardiovascular disease [2].The acronym CAKUT includes a heterogeneous spectrum of pathologies: from simple, asymptomatic morphological variations, to serious diseases, which put the patient's life at risk. Furthermore, some of these are part of syndromes that require a multidisciplinary approach. Thanks to the availability of prenatal ultrasound (US), it was possible to diagnose early most CAKUT; this fueled the hope that, by diagnosing everything immediately, it would have been possible to perform more timely surgical treatments and more careful follow-ups that would have modified the prognosis of these malformations. Unfortunately, the epidemiological study showed that precocity of diagnosis and therapeutic aggression did not modify the outcome of CAKUT[3], because their natural history varies only slightly by solving the hydraulic problems of obstruction or reflux. For this reason, research has shifted its attention to genetic and molecular origin of CAKUT[4-5]. And because of this, clinicians should plan a diagnostic program commensurate to the expected benefit: minimizing radiobiological risk, patient discomfort and economic burden. In this context, a large space must be reserved for US, which allows for a detailed evaluation of number, shape, position, volume and structure of the kidneys; for many CAKUT, US may be the only necessary for diagnosis and also for follow-up. It is the US responsibility to distinguish the clinical situations of low and medium risk from those in which other diagnostic techniques are indicated. The main limitation of US is that the result is closely related to the skill and experience of the operator, therefore maximum effort should be made to guarantee quality of the performance for all patients, through accreditation systems for sonographers, and to offer to pediatricians, who practice US, adequate training[6].

Other imaging techniques play a much more limited role in diagnosing CAKUT: *voiding cystourethrography* (*VCUG*), is often supplanted by US even in the morphological study of urethra and anatomical details of bladder, its only indication remains the diagnosis of vesicoureteral reflux (VUR). Cystosonography or cystoscintigraphy[7-8] are always valid alternatives to VCUG and respectively they cancel and limit the radiobiological risk.

When US shows a severe dilatation and an obstruction is suspected, the diagnosis of the CAKUT can be completed by sequential renal scintigraphy (with MAG 3 or DTPA) which supplies the functional data of each kidney and, completed with the diuretic test, provides the differential diagnosis between dilatation and obstruction[9].

DMSA scintigraphy will be used if it is necessary to “visualize” and "quantify" the functioning renal parenchyma or to evaluate the function in percentage terms. A role limited to complex and rare situations is reserved for Uro-RM or TC.

**References**

1. Song R, Yosypiv IV. Genetics of congenital anomalies of the kidney and urinary tract. Pediatr Nephrol. 2011;26:353-64.
2. Calderon-Margalit R, Golan E, Twig G, Leiba A, Tzur D, Afek A. History of childhood kidney disease and risk of adult end-stage renal disease. N Engl J Med. 2018;378:428-438.
3. Becherucci F, Roperto RM, Materassi M, Romagnani P. Chronic kidney disease in children. Clin Kidney J. 2016;9:583-91.
4. Sanna-Cherchi S, Ravani P, Corbani V, Parodi S, Haupt R, Piaggio G, Innocenti ML, Somenzi D, Trivelli A, Caridi G, Izzi C, Scolari F, Mattioli G, Allegri L, Ghiggeri GM. Renal outcome in patients with congenital anomalies of the kidney and urinary tract. Kidney Int. 2009;76:528-33.
5. Sanna-Cherchi S, Westland R, Ghiggeri GM, Gharavi AG. Genetic basis of human congenital anomalies of the kidney and urinary tract. J Clin Invest. 2018;128:4-15.
6. Galiano R, Novellino P. Forensic aspects and reporting ultrasound criteria. Ital J Pediatr. 2017;43:A13.
7. Darge K. Voiding urosonography with ultrasound contrast agents for the diagnosis of vesicoureteric reflux in children. I. Procedure Pediatr Radiol. 2008; 38:40-53.
8. Bosio M, Manzoni GA. Detection of posterior urethral valves with voiding cystourethrosonography with echo contrast. J Urol 2002; 168:1711-1715.
9. Nguyen HT, Benson CB, Bromley B, Campbell JB, Chow J, Coleman B, Cooper C, Crino J, Darge K, Herndon CD, Odibo AO, Somers MJ, Stein DR. Multidisciplinary consensus on the classification of prenatal and postnatal urinary tract dilation (UTD classification system). J Pediatr Urol. 2014;10:982-98.

## A46 Biomarkers for infection: a helpful tool for an early identification of serious infections

### Silvia Garazzino (silvia.garazzino@unito.it)

#### Unit of Infectious Diseases, Regina Margherita Children’s Hospital, University of Turin, Turin, Italy

Despite medical progress in diagnostics and therapeutics, sepsis remains a major cause of mortality and hospitalization in the pediatric age, both in developed and developing countries [1,2]. Sepsis may be defined as a systemic inflammatory syndrome determined by an underlying infectious process [3]. Septic shock is the progression toward a state of inadequate delivery of oxygen and metabolic substrate to tissues. Early identification and appropriate treatment of sepsis is crucial for a positive outcome [4]. In such setting, biomarkers whose levels significantly change in response to an infectious insult may serve as an helpful tool for screening and diagnosis of sepsis/severe infections, risk stratification, monitoring of response and rationalizing antibiotic use. A panel at the National Institute of Health defined a biomarker as any “characteristic that is objectively measured and evaluated as an indicator of normal biological processes, pathogenic processes, or pharmacologic responses to a therapeutic intervention” [5]. In clinical practice, the term “biomarker” is generally referred to a test performed on body fluids that easily provides information about the patients on a specific situation or process (such as serious infection or sepsis). Acute phase proteins, such as C-reactive protein, have been largely studied, whereas data on the performance of newer biomarkers of sepsis are relatively scarce in children. C-reactive protein is an easily available low-cost test, but is hampered by a low accuracy for distinguishing infection from inflammation and a long latency after the inflammatory trigger. Procalcitonin peaks earlier and has higher specificity for bacterial infections than C-reactive protein, but has also higher costs and its levels may be affected by renal dysfunction. Interleukin 6, 8 and 18 and CD64 may increase diagnostic accuracy if combined with other biomarkers and may serve as prognostic tools, but studies in the pediatric population are limited. A newer candidate biomarker for infection includes pro-Adrenomedullin, which levels correlate with severity of infection and accurately distinguish infection from inflammation, especially in febrile neutropenic children. Lactate measurement facilitates the diagnosis of septic shock and serves to monitor its progression. Human neutrophil gelatinase is a promising early biomarker of acute kidney injury. Finally, multiple stratification biomarkers are under active investigation [6,7].

**References**

1. Weiss SL, itzgerald JC, Pappachan J, Wheeler D, Jaramillo-Bustamante JC, Salloo A, Singhi SC, Erickson S, Roy JA, Bush JL, Nadkarni VM, Thomas NJ; Sepsis Prevalence, Outcomes, and Therapies (SPROUT) Study Investigators and Pediatric Acute Lung Injury and Sepsis Investigators (PALISI) Network. Global epidemiology of pediatric severe sepsis: the sepsis prevalence, outcomes, and therapies study. Am J Respir Crit Care Med. 2015;191:1147-57.

2. Kissoon N, Carcillo JA, Espinosa V, Argent A, Devictor D, Madden M, Singhi S, van der Voort E, Latour J; Global Sepsis Initiative Vanguard Center Contributors. World Federation of Pediatric Intensive Care and Critical Care Societies: Global Sepsis Initiative.World federation of pediatric intensive care and critical care societies: global sepsis initiative. Pediatr Crit Care Med. 2011;12:494-503.

3. Goldstein B, Giroir B, Randolph A. International pediatric sepsis consensus conference: definitions for sepsis and organ dysfunction. Pediatr Crit Care Med. 2005;6:2–8.

4. Dellinger RP, Levy MM, Rhodes A, Annane D, Gerlach H, Opal SM, et al. Surviving sepsis campaign guidelines committee including the pediatric subgroup. surviving sepsis campaign: international guidelines for management of severe sepsis and septic shock, 2012. Intensive Care Med. 2013;39:165-228.

5. Biomarkers Definitions Working Group. Biomarkers and surrogate endpoints: preferred definitions and conceptual framework. Clin Pharmacol Ther. 2001;69:89–95.

6. Standage SW, Wong HR. Biomarkers for pediatric sepsis and septic shock. Expert Rev Infect Ther. 2011; 9: 71-79.

7. Lanziotti VS, Póvoa P, Soares M, Silva JR, Barbosa AP, Salluh JI. Use of biomarkers in pediatric sepsis: literature review. Rev Bras Ter Intensiva. 2016; 28: 472-482.

## A47 Slow medicine in pediatrics: choosing wisely

### Andrea Gardini (info@slowmedicine.it)

#### Slow Medicine Board, Torino, Italy

Slow Medicine is a transcultural movement inside the larger international "slow " culture, comprehending Slow Food, Slow Cities, also "Slow Music". As well as Slow Food (Good, Clean and Fair Food) our mean key words are "Measured "- doing more does not mean doing better - "Respectful" -people's values expectations desires are different and inviolable, "Equitable "- appropriate and good quality care for all-. Slow Medicine promotes a change of the current paradigm of medicine from mechanical to a complex systemic view.

We think that a quality care needs the integration of new visions, knowledge, behaviour, relation, integrity, and reduce the influence of market forces over health care organisations, clinics and care.

Slow medicine promotes: better lifestyles, better workplaces, better food and water, better relations inside the community, better relations between human communities and environment; better clinical independent patient outcomes oriented research, without conflict of interests; evidence based medicine: the best literature, the best knowledge of patient's values and wishes and the best medical experience; better patient care, less "cure", promote an alliance between citizens and institutions, between patients and doctors, nurses and midwives (not just providers but healers); choosing wisely the right care only if necessary, fair and appropriate; Recognise the Disease Mongering in its features, pushers, corruption, side effects; promote health: a person is not just his/her disease but it's a complex living system with many healthy parts and potentials that can help his/her healing even if he/she is very ill; mutual knowledge between patient and doctor: every healing relationship cannot be good without an initial introduction of doctors/nurses/midwives with their patients : "Hallo, my name is.."; all of this needs a Slow Management, a Slow Organisation.

A basic international activity of Slow Medicine is the alliance with the Choosing Wisely movement.

In 5 years we involved 45 medical and nursing scientific societies, to choose each one 5 possibly inappropriate medical and nursing practices to be discussed with patients before prescribing them. This happens with the participation of the Federations of Medical and Nursing Colleges, Altroconsumo and Mario Negri's Institute "Partecipasalute" project .

The practices have been inserted into the new national guidelines plan of the National Health Institute and in the EBSCO worldwide documentation system.

We also collaborate with some regions, hospitals and health units in the project " Slow Hospitals and health units" to promote our principles and practices at local level.

An illustration of the main inappropriate paediatric practices choosen by the paediatric societies will follow.

## A48 Epidemiology of inflammatory bowel disease

### Silvia Ghione (silvia.ghione@hotmail.it)

#### Department of Pediatrics and Neonatal Care, St Jacopo Pistoia Hospital, Azienda USL Toscana Centro, Pistoia, Italy

Inflammatory Bowel Disease (IBD) included Crohn’s Disease (CD), Ulcerative Colitis (UC) and IBD Unclassified (IBDU). These different diseases can be distinguished on the basis of clinical suspicion sustained by laboratory, radiological, endoscopic and histological findings. Epidemiology is the study of occurrence of illness: it studies patterns, causes and effects of health disease conditions in defined populations and is a cornerstone for public health.

Regarding IBD, starting from the first epidemiological studies in the 1960, many studies with different study designs, different number of patients included and different length of study period have been published. Compared to adult, epidemiologic data on pediatric IBD is less available, but recently important population data has been published on this group of patients. Today it is estimated that IBD afflicts as many as 1.4 million persons in the US and Canada and 2.2 million in Europe, representing a major burden for developed countries. The incidence and prevalence of childhood-onset IBD has risen rapidly over the past two decades. While previously considered rare in children, IBD has emerged as a global disease in developed and developing nations. A clear north-south and west-east gradient exists in incidence of pediatric IBD in northern hemisphere, higher for example in the northern state of the US than southern and in Scotland than the rest of the UK. The increasingly early onset of CD and UC raises important questions regarding the role of early-life factors in the pathogenesis of IBD (mode of delivery, breastfeeding, antibiotics therapies, smoking, etc..). These same factors can explain the observation that children of immigrants assumed the IBD risk of incidence of the hosting Country, indicating that the underlying risk is activated with earlier life exposure to “west environment”.

**References**

1. Ghione S, Sarter H, Fumery M, Armengol-Debeir L, Savoye G, Ley D, Spyckerelle C, Pariente B, Peyrin-Biroulet L, Turck D, Gower-Rousseau C. Dramatic Increase in incidence of ulcerative colitis and Crohn’s disease (1988-2011): a population-based study of French adolescents. Am J Gastroenterol. 2018; 113:265-272.
2. Benchimol EI, Bernstein CN, Bitton A, Carrol MW, Singh H, Otley AR, Vutcovici M, El-Matary W, Nguyen GC, Griffiths AM, Mack DR, Jacobson K, Mojaverian N, Tanyingoh D, Cui Y, Nugent ZJ, Coulombe J, Targownik LE, Jones JL, Leddin D, Murthy SK, Kaplan GG. Trends in epidemiology of pediatric inflamatory bowel disease in canada: distributed network analysis of multiple population-based provincial health administrative databases. A m J Gastroenterol. 2017; 112:1120- 1134.
3. Wilson DC, Russel RK. Overview of paediatric IBD. Seminars in Pediatric Surgery. 2017; 26:344- 348.
4. Benchimol EI, Mack DR, Guttmann A, Nguyen GC, To T, Mojaverian N, Quach P, Manuel DG. Inflammatory bowel disease in immigrants to Canada and their children: a population-based cohort study. Am J Gastroenterol. 2015:110:553- 63.
5. Ruel J, Ruane D, Mehandru S, Gower-Rousseau C, Colombel JF. IBD across the age spectrum: is it the same disease? Nat. Rev. Gastroenterol. Hepatol. 2014:11:88-98.

## A49 Congenital malformations: when do not alert

### Mario Giuffrè, Vincenzo Antona

#### Dipartimento di Scienze per la Promozione della Salute e Materno Infantile, Università degli Studi di Palermo, Palermo, Italy

##### **Correspondence:** Mario Giuffrè (mario.giuffre@unipa.it)

Based on clinical criteria, major malformations are defined as defects causing functional impairment and therefore needing medical or surgical treatment. Defects that do not produce functional impairment and do not require medical intervention are termed minor malformations if their prevalence at birth is less than 4% and phenotypic variants when the birth prevalence is higher. Major and/or minor congenital malformations are frequently associated; apparently isolated defects may be associated with malformations that are not clinically evident at birth [1]. Therefore, most minor malformations may be isolated and should not generate any specific alert for future health issues, others may be part of a more complex phenotype with a more severe prognosis, these must be recognized early and investigated carefully.

The birth of a baby with congenital malformations is the starting point of a clinical process that is aimed at making a precise diagnosis. This leads to appropriate clinical planning and definition of prognosis and counselling of the parents [2]. Different causal pathways may lead to a similar phenotype and the diagnostic process may be long and difficult, requiring follow-up to establish the natural history of the disorder. The diagnostic process includes an accurate history, description of the phenotype, with appropriate imaging and laboratory tests [3]. Improvements in informatics have led to the development of computerized systems to improve diagnostic accuracy. The history should include consideration of the whole family (with the definition of a genealogical tree and the identification of risk factors (consanguinity, multiple abortions, stillbirths, advanced maternal and/or paternal age), the preconceptional period and environmental factors (infections, maternal metabolic diseases, diabetes mellitus, drugs, alcohol) and the pregnancy [4]. Analysis of the phenotype [5] is aimed at the identification and description (with photographic documentation) of structural defects (isolated and multiple, major and minor) and should include a description of clinical associations with genetic syndromes, such as neuropsychomotor retardation, growth restriction [6], disorders of sexual differentiation or pubertal development.

Genetic counselling is defined as the non-directive process of communicating with and giving information to a family (usually the parents) to enable the making of decisions relating to patients with genetic diseases that are considered, responsible and rational. The main factors that determine a need for genetic counselling are the presence of an index case with a congenital malformation or of genetic disease in the family as well as the presence of parental risk factors (consanguinity, advanced maternal age, recurrent abortions, mutation carrier).

**References**

1. La Placa S, Giuffrè M, Gangemi A, Di Noto S, Matina F, Nociforo F, Antona V, Di Pace MR, Piccione M, Corsello G. Esophageal atresia in newborns: a wide spectrum from the isolated forms to a full VACTERL phenotype? Ital J Pediatr. 2013; 39: 45.

2. Schierz IA, Giuffrè M, Piro E, Ortolano R, Siracusa F, Pinello G, La Placa S, Corsello G. Predictive factors of abdominal compartment syndrome in neonatal age. Am J Perinatol. 2014; 31: 49-54.

3. Giuffrè B, Parini R, Rizzuti T, Morandi L, Van Diggelen OP, Bruno C, Giuffrè M. Severe neonatal onset of glycogenosis type IV: clinical and laboratory findings leading to diagnosis in two siblings. J Inherit Metab Dis. 2004; 27: 609-619.

4. Carta M, Maresi E, Giuffrè M, Catalano G, Piro E, Siracusa F, Corsello G. Congenital hepatic mesenchymal hamartoma associated with mesenchymal stem villous hyperplasia of the placenta: Case report. J Pediatr Surg. 2005; 40: E37-E39.

5. Giuffrè M, La Placa S, Carta M, Cataliotti A, Marino M, Piccione M, Pusateri F, Meli F, Corsello G. Hypercalciuria and kidney calcifications in terminal 4q deletion syndrome: further evidence for a putative gene on 4q. Am J Med Genet A. 2004; 126A: 186-190.

6. Puccio G, Giuffré M, Piccione M, Piro E, Rinaudo G, Corsello G. Intrauterine growth restriction and congenital malformations: a retrospective epidemiological study. Ital J Pediatr. 2013; 39: 23.

## A50 HPV vaccine

### Giancarlo Icardi^1,2^, Ilaria Barberis^1^

#### ^1^Department of Health Sciences, University of Genoa, Via Pastore 1, 16132 Genoa, Italy; ^2^Policlinico San Martino – IST, Largo Rosanna Benzi, 10 16132 Genoa, Italy

##### **Correspondence:** Giancarlo Icardi (icardi@unige.it)

Human papillomavirus (HPVs) are the most common sexually transmitted viruses. Most HPV infections are asymptomatic and resolve without complications within 2 years.

Persistent infection with high-risk HPV types is responsible for most cervical and anal cancers, a large portion of cancers of the mouth and pharynx, and penile cancers.

Each year in Europe approximately 487.986 new cases of cancer in females and 422.027 new cases in males are caused by HPV. [1]

The HPV vaccine is most effective if it is given before sexual activity begins. HPV vaccines protect against cervical precancer in adolescent girls, especially for lesions associated with HPV 16/18 and in those who are negative for HPV16/18 DNA at enrolment. [2]

The rationale for routine HPV immunization at 11 through 12 years of age is twofold. First, optimal vaccine efficacy is derived if the vaccine is administered before onset of sexual activity. Second, antibody responses are highest at ages 9 through 15 years. Immunization of males provides direct benefit to males, including prevention of genital warts and anal cancer. In addition, immunization of males is expected to provide indirect benefit for females through herd immunity.

Since 2015 in Italy it is available a new nine-valent vaccine for prevention of HPV types 6, 11, 16, 18, 31, 33, 45, 52, 58, which are responsible for 89% of cases of HPV-related cancers and more than 90% of genital warts and almost all cases of juvenile recurrent respiratory papillomatosis.

The National Vaccination Prevention Plan 2017-2019 recommends the free-of-charge and active immunization against HPV for all males and females 11- through 12-year-old children as part of the adolescent immunization platform. [3]

Practices on vaccine efficacy, safety, and cost-effectiveness as well as programmatic considerations supports this recommendation. [2,4]

The vaccine is offered in 2 doses to healthy 12 years old adolescents. The first dose is given at any time. The second dose is given 6 to 12 months after the first dose.

The vaccine is also given in 3 doses to adolescents > 15 years of age. The first dose is given at any time. The second dose is given 1 to 2 months after the first dose. The third dose is given 6 months after the first dose. [5]

The HPV 9-valent vaccine represents the new standard for the largest and universal prevention of HPV-related pathologies.

**References**

1. Hartwig S, St Guily JL, Dominiak-Felden G, Alemany L, de Sanjosé S. Estimation of the overall burden of cancers, precancerous lesions, and genital warts attributable to 9-valent HPV vaccine types in women and men in Europe. Infect Agent Cancer. 2017:11;12:19.
2. Arbyn M, Xu L, Simoens C, Martin-Hirsch PP. Prophylactic vaccination against human papillomaviruses to prevent cervical cancer and its precursors. Cochrane Database Syst Rev. 2018 May 9;5:CD009069.
3. Ministero della Salute. Piano Nazionale Prevenzione Vaccinale (PNPV) 2017 – 2019. [http://www.salute.gov.it/imgs/C_17_pubblicazioni_2571_allegato.pdf. ]. Accessed on 12 July 2018.
4. Stanley M. HPV vaccination in boys and men. Hum Vaccin Immunother. 2014;10:2109-11.
5. European Medicines Agency. Annexe I. Summary of Product Characteristics. [http://ec.europa.eu/health/documents/community-register/2018/20180219140172/anx_140172_it.pdf]. Accessed on 12 July 2018.

## A51 Hepatitis C Virus infection in children

### Giuseppe Indolfi, Massimo Resti

#### Meyer Children’s University Hospital, Florence, 50139, Italy

##### **Correspondence:** Giuseppe Indolfi (g.indolfi@meyer.it)

Hepatitis C virus (HCV) is a leading cause of chronic viral hepatitis in children. Before 1992, when the universal screening of blood products began, hepatitis C was spread commonly through transfusions and organ transplants [1]. Nowadays, worldwide, children can acquire the infection from mothers with hepatitis C or through the use of blood-contaminated objects (syringes and other equipment). Transmission from mother to child is the major source of acquisition of HCV infection in children [1,2]. The exact timing and the ultimate mechanism of mother-to-child transmission of HCV infection are unknown. Understanding the mechanism of mother-to-child transmission is an unsolved challenge. Following mother-to-child transmission of HCV about 20% of the children present spontaneous clearance of the infection while the remaining develop chronic hepatitis and, if untreated, will become HCV infected adults [3]. Interferon-based therapies, which have a low efficacy and a burdensome safety profile [4], have been substituted since 2014 with the highly effective and safe new combinations of direct-acting antivirals active against HCV. Recently, in July 2017, the fixed-dose combination of sofosbuvir/ledipasvir and the combination of sofosbuvir with ribavirin have been approved by the European Medicines Agency for treatment of children older than 12 years with chronic hepatitis C virus genotype 1, 4, 5 or 6 and 2 or 3 infection, respectively. Preliminary results on the use of direct-acting antivirals in children are available for sofosbuvir/ledipasvir in children aged 6-17, for sofosbuvir and ribavirin for children older than 12 years, for the combination of ombitasvir/paritaprevir/ritonavir with or without dasabuvir, with or without ribavirin for children older than 12 years with hepatitis C virus genotype 1 or 4 infection, and for the combination of sofosbuvir with daclatasvir for children with hepatitis C virus genotype 4 infection [5]. All the results available showed excellent efficacy and optimal safety profiles of the different combinations of direct-acting antivirals. With the approval of the new drugs, indications to treatment of children with chronic hepatitis C virus have changed. Nowadays, direct-acting antiviral therapy is recommended for all the adolescents with chronic hepatitis C virus infection independent of treatment history and disease severity. As soon as direct-acting antivirals will be approved for younger age cohorts, all hepatitis C virus-infected children older than three years will benefit from antiviral therapy [6]. The goal of elimination of HCV infection as a public health threat requires inclusion of all affected populations, including children.

**References**

1. Bortolotti F, Resti M, Marcellini M, Giacchino R, Verucchi G, Nebbia G, Zancan L, Marazzi MG, Barbera C, Maccabruni A, Zuin G, Maggiore G, Balli F, Vajro P, Lepore L, Molesini M, Guido M, Bartolacci S, Noventa F. Hepatitis C virus (HCV) genotypes in 373 Italian children with HCV infection: changing distribution and correlation with clinical features and outcome. Gut. 2005; 54:852-857.
2. Indolfi G, Azzari C, Resti M: Perinatal transmission of hepatitis C virus. J Ped 2013;163:1549-1552.
3. Resti M, Jara P, Hierro L, Azzari C, Giacchino R, Zuin G, Zancan L, Pedditzi S, Bortolotti F. Clinical features and progression of perinatally acquired hepatitis C virus infection. J Med Virol 2003; 70:373-377.
4. Indolfi G, Nebbia G, Cananzi M, Maccabruni A, Zaramella M, D'Antiga L, Grisotto L, Azzari C, Resti M. Kinetic of virologic response to pegylated interferon and ribavirin in children with chronic hepatitis C predicts the effect of treatment. Ped Infect Dis J 2016; 35:1300-1303.
5. Indolfi G, Serranti D, Resti M. Direct-acting antivirals for adolescents with chronic hepatitis C. Lancet Child & Adol Health. 2018; 2:298- 304

6. Indolfi G, Hierro L, Dezsofi A, Jahnel J, Debray D, Hadzic N, Czubkowski P, Gupte G, Mozer-Glassberg Y, van der Woerd W, Smets F, Verkade HJ, Fischler B. Treatment of Chronic Hepatitis C Virus Infection in Children: A Position Paper by the Hepatology Committee of European Society of Paediatric Gastroenterology, Hepatology and Nutrition. J Ped Gastr Nutr 2018;66:505-515.

## A52 Chronic diseases and disability in migrant children: challenge and opportunity in health care

### Simona La Placa (simonalaplaca@gmail.com)

#### Department of Sciences for Health Promotion and Mother and Child Care, University of Palermo, Palermo, Italy

Today more than ever migrations are a challenge to be addressed in terms of reception, but above all an opportunity to be taken in terms of public health. Considering the interaction of health conditions, socio-cultural, and environmental factors, chronic diseases [1] and disability increase the vulnerability that all migrants would experience, especially if they are children.

The National Working Group for Migrant Children of the Italian Society of Pediatrics [GLNBM – SIP, http://www.glnbi.org] has been working for more than 25 years in order to guarantee the right to migrant child’s health and healthcare in their best interest [http://www.gruppocrc.net].

Foreign children (EU and non-EU) have become over time a relevant fraction of the resident population, with incidence of about 10% over the overall number of minors and of 9.2% of the student population [http://www.miur.gov.it]. Among migrant students it has been reported school difficulties due to the late and inadequate learning of Italian language, recent migration and psycho-social-cultural disadvantage (32.9% in foreign students *versus* 10,5% in Italian students). But also, recently, it has been registered an increasing number of migrant students with disabilities (12%). Therefore, adequate education policies are needed, including promotion of early language learning and educational activities, as well as training for teachers in issues related to migration and the presence of cultural mediators in schools.

Nowadays, Italy guarantees free foreign children’s access to pediatric health services as well as their National Healthcare System registration, independently from legal status of their parents. Nevertheless, informal barriers, in turn added to health policies, hinder access: language and intercultural communication problems, lack of social network and of knowledge about the health care system, as well as lack of cultural mediators in most primary paediatric health centers [2].

At least, international adoption presents unique challenges that require support for the adoptive families throughout all the adoption process, because of a growing number of international adoptions involve kids who are older and have special needs, and in order to avoid adoption crises or “failures”.

Greater understanding of the elevated risk posed by migration to disabled migrant children is needed along with strategies to mitigate risks. The particular vulnerability of migrant minors due to social fragility and inadequate reception, impose multidisciplinary and transcultural approach [3], increasingly widespread. Delivering healthcare is a collective activity to increase public awareness and combine efforts of disabled people, their families and communities, as well as relevant governmental agencies and NGOs, educational and social services, within a framework where social inclusion, equal opportunities, and, as a consequence, a good quality of life of disabled people prevails.

**References**

1. Montesi L, Turchese Caletti M, MarchesiniG. Diabetes in migrants and ethnic minorities in a changing World. World J Diabetes 2016;10:7:34-44.

2. Battersby A, Guðmundsdóttir H, Heller Y, Hjern A, Jäger A, Jensdóttir EH, Kadir A, Kovács Z, Kyeremateng R, Martin L, Métraux J-C, Rubio B, Sievers E, Tsitoura S, von Folsach LL. ISSOP position statement on migrant child health. Child Care Health Dev. 2017;1–10.

3. Pulido-Fuentes M, Abad González L, da Silva Vieira Martins M, Flores Martos JA. Health competence from a transcultural perspective. Knowing how to approach transcultural care. Procedia - Social and Behavioral Sciences. 2017; 237:365 – 372.

## A53 Nasal cytology: practical aspects and clinical relevance

### Massimo Landi (landi@alma.it)

#### National Pediatric Healthcare System, Turin, Italy and Unit Research of Pediatric Pulmonology and Allergy, Institute of Biomedicine and Molecular Immunology (IBIM), National Research Council, Palermo, Italy

Nasal cytology is a simple and safe method that allows to evaluate the possible inflammatory state of the nasal mucosa. From a technical point of view it consists of a delicate passage with a tool (nasal scraping) on ​​the mucosa of the inferior turbinate, the deposition of the material on a slide and the subsequent coloring with May Grumwald Giemsa. The preparation will then be read with a 100 X microscope according to a semi-quantitative and descriptive reading (table I). (1)

The reading allows to distinguish the cells of the nasal mucosa and the inflammatory cells (neutrophils, eosinophils and mast cells) as well as the presence of fungi and bacteria.

This diagnostic therefore becomes important as a diagnostic tool in allergic forms with minimal persistent inflammation, typical of mites, (2) or in the differential diagnosis of overlapping rhinitis (allergic and Nares) (3) and in the diagnosis of non-allergic forms such as Nares, Naresma or Narma, (Fig 1,2,3) in which the diagnosis can not be a simple exclusion of allergic sensitization but must be circumstantiated with the presence of eosinophils or mast cells.

Nasal cytology therefore becomes an important tool for precision medicine (4) thus allowing targeted therapy.

**References**

1. Gelardi M, Iannuzzi L, Quaranta N, Landi M, Passalacqua G. Nasal cytology: practical aspects and clinical relevance. Clin Exp Allergy. 2016;46:785-92.
2. Gelardi M, Landi M, Ciprandi G. Nasal cytology: a precision medicine tool in clinical practice. Clin Exp Allergy. 2018;48:96-97.
3. Gelardi M, Luigi Marseglia G, Licari A, Landi M, Dell'Albani I, Incorvaia C, Frati F, Quaranta N. Nasal cytology in children: recent advances. Ital J Pediatr. 2012:38:51.

**Table 1 (abstract A53).**
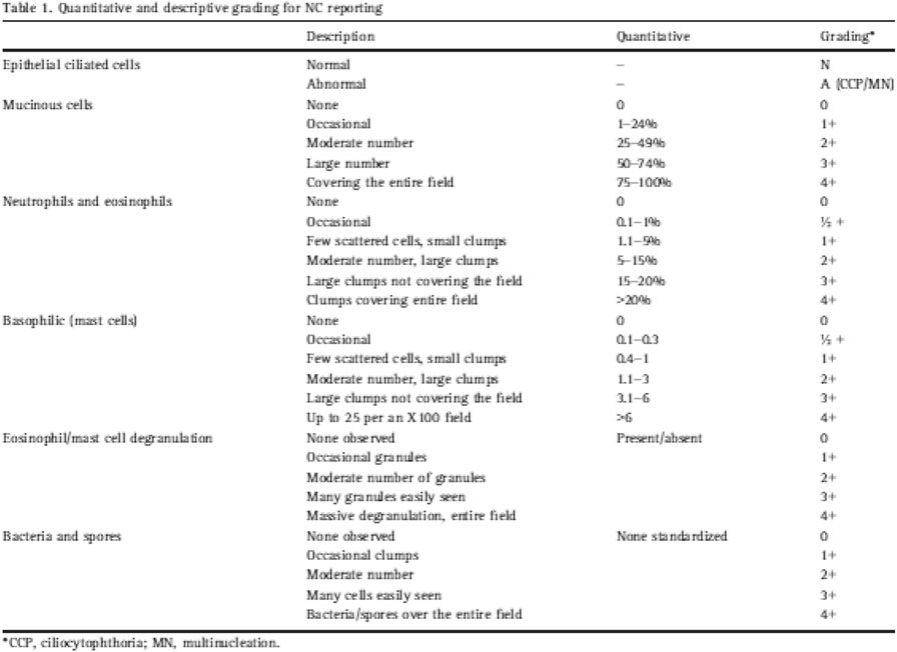
Quantitative and descriptive grading for NC reporting

**Fig. 1 (abstract A53).**
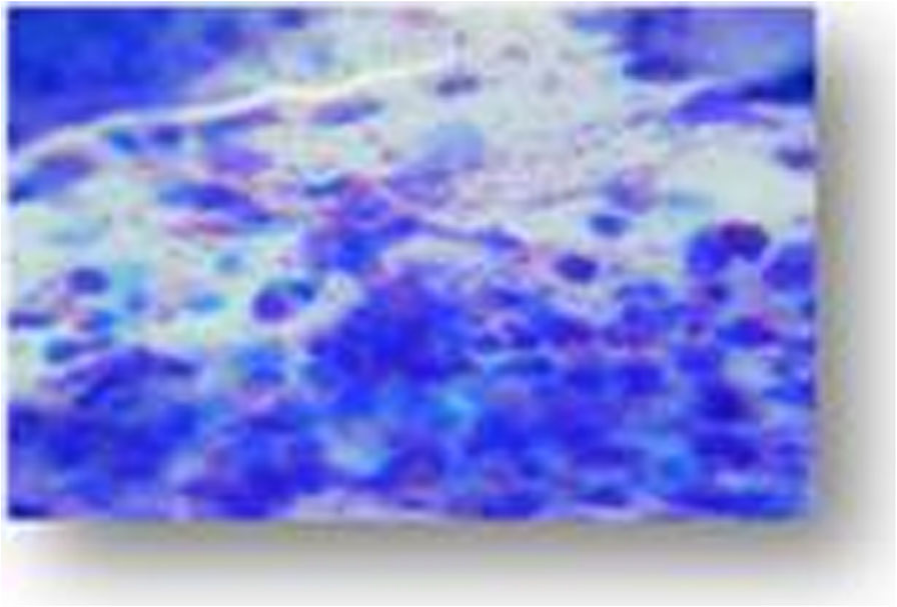
See text description

**Fig. 2 (abstract A53).**
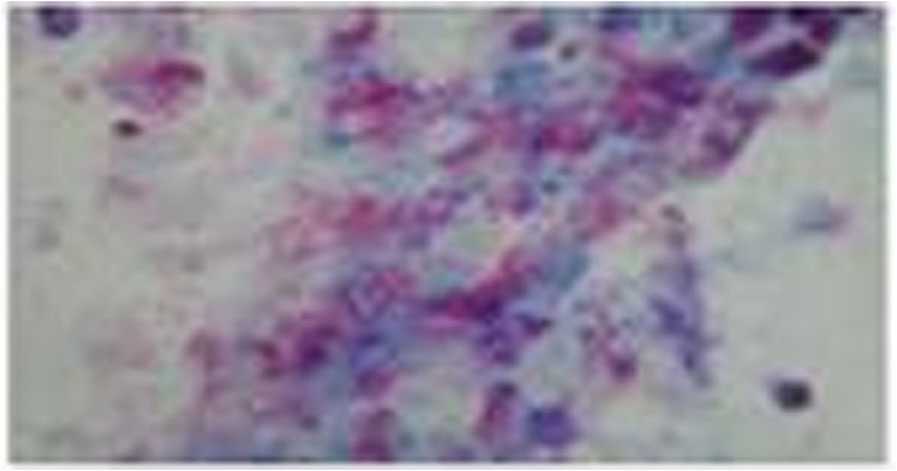
See text description

**Fig. 3 (abstract A53).**
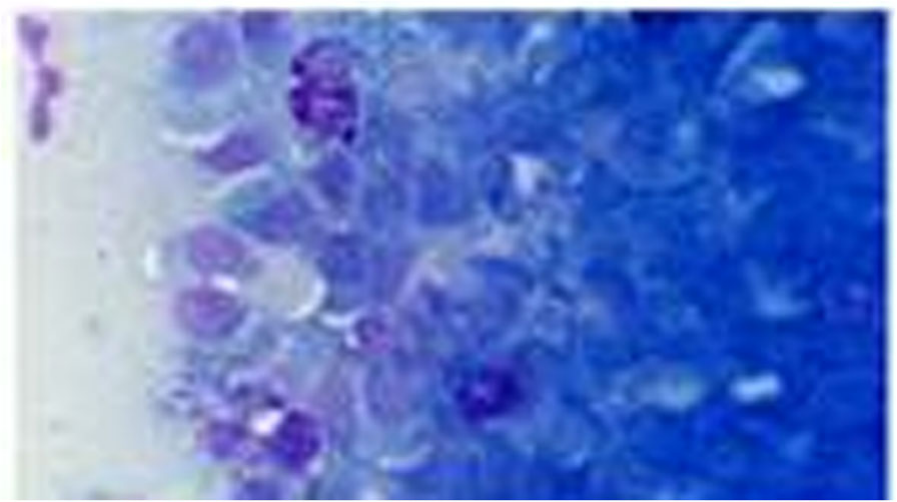
See text description

## A54 Immunogenicity and nutritional value of protein-hydrolysed formulas for cow’s milk allergy

### Amelia Licari, Gian Luigi Marseglia

#### University of Pavia, Fondazione IRCCS Policlinico San Matteo, Pavia, Italy

##### **Correspondence:** Amelia Licari (a.licari@smatteo.pv.it)

Cow’s milk allergy (CMA) is the most common food allergy in childhood, affecting 2–7 % of children [1]. The mainstay of treatment of CMA is strict avoidance of cow’s milk proteins. Breastfeeding, if still possible, is the first choice for CMA patients. In the absence of breast milk, international guidelines recommend the prescription of a substitute formula until at least 2 years of age, in order to fulfill the nutritional requirements of young children [1]. This type of formulas should be adequate in terms of allergic efficacy and nutritional safety. Substitute formulas include extensively hydrolyzed formulas (EHF) based on caseins or whey proteins, amino acid formulas (AAF), and non-cow’s milk-based formulas based on soy and rice proteins [2]. As the allergic condition may expose the infant with CMA to early growth impairments, the macronutrient composition of substitute formulas should reasonably be taken into account. The current literature supports the nutritional adequacy of available cow’s milk substitutes both during the first and second semesters of life, with some differences between products [1]. With particular reference to protein hydrolysates, some studies also demonstrated a hypoallergenic immunomodulating effect, favoring the induction of tolerance towards cow’s milk proteins [3].

Therefore, an ideal substitute formula for CMA should be well tolerated, lead to remission of allergic symptoms, and allow proper and even catch-up growth of the affected children.

**References**

1. Fiocchi A, Brozek J, Schünemann H, et al. World Allergy Organization (WAO) Diagnosis and Rationale for Action against Cow's Milk Allergy (DRACMA) Guidelines. WAO J. 2010;3:57-161.
2. Vandenplas Y. Prevention and management of cow's milk allergy in non-exclusively breastfed infants. Nutrients. 2017;9: E731.
3. Kiewiet MBG, Gros M, van Neerven RJJ, et al. Immunomodulating properties of protein hydrolysates for application in cow's milk allergy. Pediatr Allergy Immunol. 2015;26:206-217.

## A55 Alarm signs in growth curves (height, weight and BMI)

### Sandro Loche, Chiara Guzzetti

#### SSD Endocrinologia Pediatrica e Centro Screening Neonatale, Ospedale Pediatrico Microcitemico “A. Cao”, AO Brotzu, Cagliari, Italy

##### **Correspondence:** Sandro Loche (sandroloche@asl8cagliari.it)

In a growing child a normal growth pattern usually indicates absence of chronic disease. Thus, regular measurement of weight and height is of paramount importance in daily pediatrics care. Height and weight need to be measured at least every year in school-aged healthy children, and more frequently in newborns and toddlers. Appropriate growth charts help the paediatricians in the evaluation of normal or abnormal growth. Therefore, it is essential to know how to interpret the growth curve and recognize when the growth pattern is abnormal. Besides weight and height measurements, other parameters like height velocity, target height, pubertal status, body mass index (BMI), and body proportions need to be considered. The anthropometric measurements should be as accurate as possible, best done with the same instrument at each visit, in order to avoid wrong interpretations of the results. Short stature, under or overweight, and abnormal body proportions are the most common growth abnormalities found in the general pediatric setting.

Short stature is defined as height <2 standard deviations from mean height of healthy children from the same population. Short stature can be associated with growth failure. Deceleration of growth even in a normal statured child is always an alarm signal, and the child needs to be promptly investigated. Short stature can be due to a variety of conditions and in every short child first line blood testing and bone age assessment are warranted. National guidelines indicate the criteria to perform growth hormone secretion studies. Idiopathic short stature (familial) and constitutional delay of growth and puberty are the most common causes of short stature, but in most children pathological causes need to be excluded.

Overweight and obesity are a major health problem also in children, due to the high frequency and the high rate of complications and comorbidities. A child <2 years of age is defined obese if his/her weight for length is ≥97.7^th^ percentile on WHO charts. An older child is defined overweight when BMI is ≥85^th^ percentile, and obese if BMI is ≥95^th^ percentile. Obese children need to be screened for possible comorbidities (dyslipidemia, hypertension, glucose tolerance, non-alcoholic fatty liver disease). Obesity is seldom caused by an endocrine disorder (Cushing disease, hyoithyroidism, hypoparathyroidism, genetic syndromes). Endocrine investigation should be reserved to selected cases. Since weight loss in the obese child is a difficult task, early identification of overweight children is of primary importance to prevent obesity and its complications.

## A56 Complementary medicine in the treatment of pediatric chronic diseases

### Francesco Macrì (profmacri@gmail.com)

#### Department of Pediatrics - “Sapienza” University of Rome, Rome, Italy

Life expectancy is now longer than ever before. In Italy, it is now 80.6 years for men and 84.9 years for women (Istat, 2017). However, while public health policies and interventions have led to the almost total eradication of most acute diseases, chronic diseases such as cancer, cardiovascular diseases and degenerative conditions are on the rise, due in part to factors such as unhealthy lifestyle and unfavourable environmental conditions.

In conventional medicine, chronic diseases are essentially treated by controlling their symptoms, with the aim of ensuring the patient’s quality of life. Under this model, the clinical evolution or resolution of a disease process is considered a solely pharmacological result, not taking into consideration the individual reactivity of patients as happen in the field of complementary and alternative medicine (CAM).

There are now more than a hundred different types of CAM. Of particular note are those defined as Medical Systems because their complexity, such as acupuncture, homeopathy, homotoxicology, anthroposophy and Ayurvedic Medicine. Medical Systems in particular, aim to lead patients towards healing. However, they also have the less ambitious objective of providing a support to reduce the use of conventional medications, given their side effects. In oncology a study published in 2014 revealed that 37.5 % of breast cancer patients had used CAM, especially young patients with a high educational level. Of these, 79.7% had used homeopathy or phytotherapy, 42% physical therapy, and 31.9% diet therapy; 65.8% reported benefiting from their treatment, with an absence of side effects in 74.8% of cases [1].

In paediatric cancer patients, such treatments can lead to an effective improvement in the patient- doctor relationship, as documented in a article investigating 98 paediatric cancer patients treated with anthroposophy [2]. It has also been found that homeopathic remedies can have an apoptotic effect on cancer cells [3].

**References**

1. Saghatchian M, Bihan C, Chenailler C, Mazouni C, Dauchy S, Delaloge S. Exploring frontiers: use of complementary and alternative medicine among patients with early-stage breast cancer. Breast. 2014;23:279-85.
2. Läengler A, Spix C, Edelhäuser F, Martin DD, Kameda G, Kaatsch P, Seifert G. Anthroposophic medicine in paediatric oncology in Germany: results of a population-based retrospective parental survey. Pediatr Blood Cancer. 2010;55:1111-7.
3. Mondal J, Samadder A, Khuda-Bukhsh AR. Psorinum 6 x trigger apoptosis signals in human lung cancer cells. J Integr Med. 2016;14:143-53.

## A57 Respiratory infections: an interaction between the immune system, environment and diet

### Chiara Mameli, Dario Dilillo, Marco Burrone, Arianna Sangiorgio, Gian V Zuccotti

#### Department of Pediatrics, V. Buzzi Children’s Hospital, University of Milan, Milan, Italy

##### **Correspondence:** Chiara Mameli (chiara.mameli@unimi.it)

Respiratory infections (RIs) are the most common diseases among children. They are responsible for significant morbidity in preschool children and impose an enormous burden on both healthcare system and society [1]. Both environmental and host factors are important in determining the individual’s susceptibility to RIs. Some of them are well known, whereas new factors, such as diet and physical activity, have been only recently investigated.

In the first years of life, the recurrence of RIs is common, caused by increased exposure to infectious agents when the immune system is still not completely developed [2]. In fact, both the innate and adaptive arms of the immune system are immature at birth, undergo a quite prolonged postnatal maturation and reach full maturity in late childhood. The postnatal maturation of the immune system is driven by the environmental exposure to pathogens. A delayed maturation of adaptive immune function represents a risk factor for RIs in the first years of life [3].

The influence of physical activity on the immune system and effects of different amounts and intensity of physical activity on respiratory symptoms were firstly studied some years ago. Recently, some authors showed that sedentary lifestyle is associated with a medium risk of upper respiratory infections, whereas a moderate physical activity with a low risk. The physical activity is supposed to increase the production of stress hormones, to reduce excessive local inflammation and to skew the immune response away from a T Helper 1 toward a T Helper 2 phenotype [4].

Finally, evidence has rapidly been accumulating about the immune-modulating abilities of foods, and, recently, the field of nutrition and respiratory disease has continued to expand [5-7]. The immunomodulating effects of micronutrients, vitamins and mineral supplements have been studied in both adults and children with conflicting and inconclusive results. Whether some dietary pattern may reduce or increase the risk of respiratory symptoms and infections in children, it is an interesting field for further research.

**References**

1. Mameli C, Penagini F, Zuccotti GV. Recurrent respiratory infections in children. In: Berhardt Leon V. Advances in Medicine and Biology. Volume 70. New York, Nova science publisher; 2013.187-206.
2. Zuccotti GV, Mameli C. Respiratory infections and immunostimulants: an update. J Pediatr Neonat Individual Med. 2015;4:e040218.
3. Tregoning JS, Schwarze J. Respiratory viral infections in infants: causes, clinical symptoms, virology, and immunology. Clin Microbiol Rev. 2010;23:74-98.
4. Martin SA, Pence BD. Exercise and respiratory tract viral infections. Exerc Sport Sci Rev. 2009;37:157-164.
5. Calder PC. Feeding the immune system. Proc Nutr Soc. 2013;72:299-309.
6. Berthon BS, Wood LG. Nutrition and respiratory health-feature review. Nutrients. 2015;7:1618-1643.
7. Tromp II, Kiefte-de Jong JC, de Vries JH, Jaddoe VW, Raat H, Hofman A, de Jongste JC, Moll HA. Dietary patterns and respiratory symptoms in pre-school children: the Generation R Study. Eur Respir J. 2012;40:681-689.

## A58 Bronchopulmonary dysplasia: from northway to metabolomics

### Danila Manus, Valentina Masile, Maria C Pintus, Angelica Dessì, Vassilios Fanos

#### Neonatal Intensive Care Unit, Neonatal Pathology and Neonatal Section, AOU and University of Cagliari, Cagliari, Italy

##### **Correspondence:** Danila Manus (danila.manus@virgilio.it)

Bronchopulmonary dysplasia (BPD) is a common sequelae of premature birth. The term was coined by Northway et al. in 1967 to indicate a chronic pulmonary condition observed in preterm infants with respiratory distress syndrome treated with high oxygen concentrations and mechanical ventilation [1]. This disease, later named “old BPD”, was histopathologically characterized by intense airway inflammation, heterogeneous lung injury, and parenchymal fibrosis [2].

Since its first description, the definition of BPD has undergone several revisions. Currently, BPD is most often defined using the National Institute of Child Health and Human Development consensus criteria based upon oxygen requirement (≥28 days), gestational age (<32 weeks vs ≥32 weeks) and severity (mild, moderate, severe) depending on oxygen use and/or respiratory support at 36 weeks postmenstrual age or at 56 days of postnatal age [3]. Nevertheless, efforts are being made to refine this definition [4].

Many advances in neonatal-perinatal medicine (prenatal steroids, surfactant replacement, and gentler ventilation strategies), shifted the demographic characteristics of BPD to extremely preterm infants, born with structurally immature lungs and underdeveloped pulmonary vasculature [5]. The early disruption of normal lung development by preterm birth would evolve into aberrations in both alveolarization and pulmonary vasculogenesis. These abnormalities have defined the concept of “new BPD” which is histopathologically characterized by simplification of the parenchyma and dysmorphic pulmonary vasculature [6-9].

Preventive strategies (prevention of infections, optimization of nutrition and ventilation) have been proven to minimize lung injury; nevertheless, to date BPD remains without satisfactory therapies [10].

Currently, research has focused on identification of biomarkers to advance detection, to improve prognostic outcome, and to begin an appropriate treatment that can prevent later complications of BPD. In this regard, the “omics” sciences are promising approaches to identify novel biomarkers. Metabolomics, one of the newer “omics”, has the ability to identify changes in metabolites caused by interaction between specific pathophysiological states, gene expression and environment. The application of clinical metabolomics in BPD has been described in literature and the results may be promising [11-14].

This information, combined with data from genetic and epigenetic studies, will contribute to the development of more effective diagnostic tools, the discovery of molecular pathways associated with the development and progression of the disease and the identification of new therapeutic targets. In conclusion, the goal of biomarkers identification should be to obtain tailored therapies for each individual patient, thereby reducing side effects and improving response to treatment.

**References**

1. Northway W, Rosan R, Porter D. Pulmonary disease following respirator therapy of hyaline-membrane disease. Bronchopulmonary dysplasia. N Engl J Med 1967;276:357-68.

2. Baraldi E. Filippone M. Chronic lung disease after premature birth. N.Engl J Med 2007; 357: 1946-1955.
3. Jobe AH, Bancalari E. Bronchopulmonary dysplasia. Am J Respir Crit Care Med. 2001;163:1723-9.

4. Jobe A.H., Steinhorn R. Can we define bronchopulmonary dysplasia? Jour of Ped. 2017; 188:19-23.
5. Bhandari A, Bhandari V. ”New”bronchopulmonary dysplasia: a clinical review. Clin Pulm Med 2011;18:137–43.
6. Altit G, Dancea A, Renaud C, Perreault T, Lands LC. Sant'Anna G. Pathophysiology, screening and diagnosis of pulmonary hypertension in infants with bronchopulmonary dysplasia - A review of the literature. Paediatric Respiratory Reviews 2017; 23:16-26.

7. Mourani PM, Abman SH. Pulmonary vascular disease in bronchopulmonary dysplasia: pulmonary hypertension and beyond. Curr Opin Pediatr. 2013;25:329-37.
8. Alvira CM. Aberrant pulmonary vascular growth and remodeling in bronchopulmonary. Dysplasia Front Med. 2016; 3:21.
9. Voynow JA. “New” bronchopulmonary dysplasia and chronic lung disease. Paediatric Respiratory Reviews, 2017;24:17-18.
10. Aschner JL, Bancalari EH, Mc Evoy CT. Can we prevent Bronchopulmonary dysplasia. J Pediatr. 2017 Oct;189:26-30.
11. Lal CV, Ambalavanan N. Biomarkers, early diagnosis, and clinical predictors of bronchopulmonary dysplasia. Clinics in Perinatology 2015;42:739-754.
12. Rivera L, Siddaiah R, Oji-Mmuo C, Silveyra GR, Silveyra P. Biomarkers for bronchopulmonary dysplasia in the preterm infant. Frontiers in Pediatrics. 2016;4:33.
13. Fanos V, Pintus MC, Lussu M, Atzori L, Noto A, Stronati M, Guimaraes H, Marcialis MA, Rocha G, Moretti C, Papoff P, Lacerenza S, Puddu S, Giuffrè M, Serraino F, Mussap M, Corsello G. Urinary metabolomics of bronchopulmonary dysplasia (BPD): preliminary data at birth suggest it is a congenital disease. J Matern Fetal Neonatal Med. 2014;27:39-45.
14. Piersigilli F, Bhandari V. Biomarkers in neonatology: the new “omics” of bronchopulmonary dysplasia. J. Matern Fetal Neonatal Med 2016; 29:1758-64.

## A59 Strategies for reducing the use of antibiotics in the NICU and for prevention of infections

### Paolo Manzoni^1,2^, Elena Tavella^1^, Alessandro Messina^1^, Marta Pieretto^1^, Eleonora Tognato^2^, Anna Perona^2^

#### ^1^Neonatology and NICU, AOU Città della Salute e della Sceienza, S.Anna Hospital, Torino, Italy; ^2^Divisione of Pediatrics and Neonatology, Degli Infermi Hospital, Biella, Italy

##### **Correspondence:** Paolo Manzoni (paolomanzoni@hotmail.com)

Neonatal sepsis is associated with a huge burden of morbidity and mortality.

Infections occurring in the neonatal period include bloodstream, urine, cerebrospinal, peritoneal, and lung infections as well as infections starting from burns and wounds, or from any other usually sterile sites.

For many pathogens, peripheral colonization usually precedes systemic infection, however many episodes of sepsis are actually caused by a breakthrough, pathogenic microorganism.

Sepsis in neonates, especially when preterms, are associated with cytokine - and biomediator-induced disorders of respiratory, hemodynamic, and metabolic processes.

Neonates in the neonatal intensive care unit feature many specific risk factors for bacterial and fungal sepsis. Prematurity is the most important risk factor, since the incidence rates of sepsis are linearly associated with decreased gestational age and birth weight. In addition, loss of gut commensals such as Bifidobacteria and Lactobacilli spp, as occurs with prolonged antibiotic treatments, delayed enteral feeding, or nursing in incubators, translates into proliferation of pathogenic microflora and abnormal gut colonization.

Empirical antibiotic treatment is popular in preterm neonates because of severity of sepsis and in lack of early, reliable laboratory sepsis markers.

No conclusive data exist, though, on the appropriate duration of antibiotic courses. These two factors often determine an overuse of antibiotics (either in number of infants exposed, or in duration of exposure in single patients). This overuse is associated with a number of potential adverse outcomes, such as selection of multidrug-resistant organisms, invasive candidiasis, necrotising enterocolitis, increased costs, modification and disruption of the normal development of the gut microbiota potentially impacting on late gut health and immunity.

A wide range of measures can be implemented in order to prevent overuse of antibiotics nonetheless preventing neonatal infections.

Improvement in diagnostic skills is mandatory, and this goal can be pursued by implementing the use of novel diagnostic methodologies like the heart –rate frequency characteristics, and the gradient in central vs. peripheral body temperature.

Withdrawal of unnecessary exposure when empirical use has been performed is equally advisable, meaning that antibiotics may be suspended if the infection is not clinically and microbiologically confirmed after 48-72 hrs. Shorter courses may be also envisaged when infection is confirmed.

A wide range of preventative measures can be implemeneted, since overuse of antibiotics may obviously be limited by preventing infections.

Promotion of breast-feeding and – more generally – nutritional and feeding strategies enhancing the role of human fresh milk are of utmost importance. Hygiene measures are a key step, as the greatest proportion of infections in neonates are acquired nosocomially.

Adoption of a cautious central venous catheter policy, including DVC bundles, is critical to decrease the frequency of catheter-associated infections (CLABSI).

Enhancement of the enteric microbiota composition with the supplementation of probiotics, medical stewardship concerning H2blockers with restriction of their use, all of this translates into a better chance for the neonate host to develop a competent enteric microbiota thus enhancing some defensive mechanisms at gut level.

Use of drugs or bioactive substances in prevention is also important, and its usefulness has been consistently underlined in the last decade. Bovine lactoferrin supplementation can prevent late-onset sepsis by any agent in preterm neonates, whereas fluconazole and (to a lesser extent) nystatin can prevent invasive systemic fungal infections.

Finally, specific measures are warranted to prevent ventilator associated pneumonia (VAP), and among them, early weaning from mechanical ventilation is the most impacting measure.

## A60 The experience of “Associazione Culturale Pediatri” (ACP) pediatric news letter

### Maddalena Marchesi^1^, Simona Di Mario^2^, Luca Ronfani^3^, Laura Reali^4^, Costantino Panza^1^, Federica Zanetto^5^, Roberto Buzzetti^6^

#### ^1^Local Health Authority - IRCSS, Reggio Emilia, Italy; ^2^Primary Care Service, Regional Health Authority of Emilia-Romagna, Bologna, Italy; ^3^Institution for Maternal and Child Health - IRCCS "Burlo Garofolo", Trieste, Italy; ^4^Local Health Authority of Rome, RM/E, Roma, Italy; ^5^Local Health Authority of Milano, Milano, Italy; ^6^Department of Experimental Medicine, "Sapienza" University of Rome, Rome, Italy

##### **Correspondence:** Maddalena Marchesi (madi.marchesi@gmail.com)

The “Associazione Culturale Pediatri” (ACP) pediatric newsletter (NP) is a resource for professional education and development.

The NP is a document that critically evaluates recently published articles in international journals on primary studies, secondary studies, guidelines, with attention to critical appraisal skills and completed by a short commented bibliography on the subject of the study.

The NPs are developed during a pediatric journal club.

Participants meet regularly to review and critique research articles, to improve their understanding of research design, statistics and critical appraisal to determine the validity and applicability of the evidence, which is then used to inform clinical decisions

The number of ACP journal club has grown consistently since its birth in 2004: from 3 to 12, involving 110 pediatricians. Since 2012, the NPs have been revised and published on the Quaderni ACP website and since 2015 they have been a regular section of the 'Electronic Pages of Quaderni ACP' [https://www.acp.it/pagine-elettroniche].

The number of monitored journals has grown over time as well, to cope with advancements in the field and new needs. One hundred and fifteen NPs have been published since 2014. Training courses on Evidence Based Medicine (EBM) are periodically organized to implement participant’s critical appraisal skills.

In a context like the present one, in which often even scientific news is consumed quickly, where the good name of a magazine or the fame of an author don't necessarily guarantee the publication's quality, cultivating together, in groups, with independent training, a reading mode that goes beyond the rapid superficial knowledge, training a thought capable of going deep, centered on the real needs of the patient and attentive to conflicts of interest can represent an important opportunity for professional growth with practical consequences both for those who produce NPs that for those who have the opportunity to read them.

## A61 Cardiovascular risk in children with chronic diseases

### Silvio Maringhini (smaringhini@ismett.edu)

#### Department of Pediatrics, ISMETT, Palermo, 90100, Italy

Cardiovascular diseases (CVD) including coronary heart disease, myocardial infarction, congestive heart failure, stroke, peripheral artery disease represent the main cause of mortality in developed Countries [1]. Several epidemiologic studies have proved that family history of CVD, age, gender, perinatal factors, overweight, tobacco exposure, hypertension, dyslipidemia represent risk factors for development of CVD [2]. Vascular lesions that produce CVD may be detected by laboratory investigations (e.g. hypercholesterolemia, microalbuminuria, etc.) or instrumental investigations (e.g. pulse wave velocity, wall thickness, myocardial ultrasound, etc.). Vascular lesions have also been found in children and have been related to some risk factors [3]. Diabetes mellitus, hypertension, dyslipidemia, chronic renal failure, obesity in adults are clearly associated with CVD [4] and children affected by these disease develop CVD earlier than adults. Furthermore, new chronic diseases in children have been associated with the risk to develop CVD such as : Endocrine diseases (Hyperthyroidism, Cushing disease, Hyperaldosteronism, Hyperpatathyroidism); Cardiovascular diseases (Kavasaky disease, cardiac valvular diseases, large artery malformations); Metabolic diseases ( hyperomocisteinemia, glycogenosis); Neoplastic diseases (acute lymphoblastic leukemia, lymphoma); Autoinflammatory diseases (SLE, rheumatoid arthritis); Organ Transplantations; Migrane; Psycosis [5-11]. Several mechanisms have been implicated in the pathogenesis of the vascular damage in those diseases including genetic background, physical inactivity, wrong diet, chronic inflammation, and drugs. A careful investigation and a prompt individualized intervention is recommended [4].

**References**

1. GBD 2016. Causes of death collaborators. Global, regional, and national age-sex specific mortality for 264 causes of death, 1980–2016: a systematic analysis for the Global Burden of Disease Study 2016. Lancet. 2017;390:1151–210.
2. Sethna CB, Merchant K, Reyes A. Cardiovascular disease risk in children with kidney disease. Semin Nephrol. 2018;38:298-313.
3. Li S, Chen W, Srinivasan SR, Berenson GS. Childhood blood pressure as a predictor of arterial stiffness in young adults: the bogalusa heart study. Hypertension. 2004;43:541-6.
4. Khambhati J, Allard-Ratick M, Dhindsa D, Lee S, Chen J, Sandesara PB, O'Neal W, Quyyumi AA, Wong ND, Blumenthal RS, Sperling LS.. The art of cardiovascular risk assessment. Clin Cardiol. 2018. doi: 10.1002/clc.22998.
5. Sadurska E, Zaucha-Prażmo A, Brodzisz A, Kowalczyk J, Beń-Skowronek I. Premature atherosclerosis after treatment for acute lymphoblastic leukemia in childhood. Ann Agric Environ Med. 2018;25:71-76.
6. Major RW, Cheng MRI, Grant RA, Shantikumar S, Xu G, Oozeerally I, Brunskill NJ, Gray LJ. Cardiovascular disease risk factors in chronic kidney disease: A systematic review and meta-analysis. PLoS One. 2018:13:e0192895.
7. De la Torre Villalobos M, Martin-López LM, Fernández Sanmartín MI, Pujals, Altes E, Gasque Llopis S, Batlle Vila S, Pérez-Solá V, Novo Navarro P, Gómez Simón I, Fresno González C, Camprodon Rosanas E, Bulbena Vilarrasa A. Cardiovascular and metabolic monitoring of children and adolescents on antipsychotic treatment: a cross-sectional descriptive study. Rev Psiquiatr Salud Ment. 2018;11:19-26.
8. Nagy G, Németh N, Buzás EI. Mechanisms of vascular comorbidity in autoimmune diseases. Curr Opin Rheumatol. 2018;30:197-206.
9. Adelborg K, Szépligeti SK, Holland-Bill L, Ehrenstein V, Horváth-Puhó E, Henderson VW, Sørensen HT. Migraine and risk of cardiovascular diseases: Danish population based matched cohort study. BMJ. 2018;360:96.
10. Ubukata M, Hara M, Nishizawa Y, Fujii T, Nitta K, Ohta A. Prevalence and mortality of chronic kidney disease in lymphoma patients: A large retrospective cohort study. Medicine (Baltimore). 2018;97:e9615.
11. Ruscitti P, Margiotta DPE, Macaluso F, Iacono D, D'Onofrio F, Emmi G, Cantatore FP, Triolo G, Afeltra A, Giacomelli R, Valentini G. Subclinical atherosclerosis and history of cardiovascular events in Italian patients with rheumatoid arthritis: Results from a cross-sectional, multicenter GIRRCS (Gruppo Italiano di Ricerca in Reumatologia Clinica e Sperimentale) study.Medicine (Baltimore). 2017;96:e8180.

## A62 Otogenic lateral sinus thrombosis: a case report

### Pasquale Marsella, Sara Giannantonio, Alessandro Scorpecci

#### Audiology and Otosurgery Unit, Surgery Department, Bambino Gesù Pediatric Hospital, Rome, Italy

##### **Correspondence:** Alessandro Scorpecci (alessandro.scorpecci@opbg.net)

**Background**

Otogenic lateral sinus thrombosis (OLST) is a rare but potentially fatal disease affecting the pediatric population and generally occurring as a complication of acute otitis media and mastoiditis [1]. Hypothesized pathophysiological mechanisms include cytokine release by the inflammatory process and activation of the coagulation pathway [2]. In case of high clinical suspicion, diagnosis should be confirmed by a pre- and post-contrast CT scan and, where available, by an urgent venography-MRI [3].

The therapeutic management of OLST remains debated. Several authors [4-9] even question the importance of anticoagulation, expressing concerns about the possible risk for hemorrhage, while considering surgical drainage of the mastoid mandatory.

**Case Report**

Seven year-old girl affected by bilateral coloboma of the iris, congenital horizontal nystagmus and a history of recurrent headache. She came to our institution’s emergency department because of the sudden onset of fever (T > 39o C), associated with nausea and vomiting. Otoscopy revealed bilateral otitis media with effusion, whereas no signs of acute mastoiditis were present. On the following day, she returned to the emergency department because of the onset of frontal headache and dizziness. An urgent CT scan with contrast of the head showed thrombosis of the left sigmoid and transverse sinus, associated with mastoid obliteration by soft tissue density material, with no bone erosion. Lateral sinus thrombosis was confirmed by brain MRI. The patient was immediately started on anticoagulant therapy. A complete thrombophilic screening was undertaken, which resulted in a heterozygous state for the MTHFR C677T mutation. Within 48 hours from diagnosis, the patient received surgical treatment consisting of left ear mastoidectomy and tympanostomy tube placement. After surgery, her symptoms disappeared, however she continued anticoagulation until control imaging 1 month after diagnosis, which showed partial recanalization of her lateral sinus.

**Conclusions**

This case report shows that the pediatrician should suspect otogenic lateral sinus thrombosis even when the patients do not present with a frank acute mastoiditis, but only aspecific otoscopic findings such as otitis media with effusion. When clinical suspicion is raised, patients should be studied right-off with CT scan of MRI including angio-sequences. Once the diagnosis is confirmed, the pediatrician should immediately consult the hematologist to screen for thrombophilia and start anticoagulant therapy, and the otolaryngologist for an indication to surgical treatment.

**Consent to publish:** a written informed consent to the publication of the case has been obtained from the girl’s parents.

**References**

1. Sebire G, Tabarki B, Saunders DE, Leroy I, Liesner R, Saint-Martin C, Husson B, Williams AN, Wade A, Kirkham FJ. Cerebral venous sinus thrombosis in children: risk factors, presentation, diagnosis and outcome. Brain. 2005; 128:477**-**489.

2. Levi M, Keller TT, van Gorp E, ten Cate H. Infection and inflammation and the coagulation system. Cardiovasc Res. 2003; 60:26-39.

3. Chalmers E, Ganesen V, Liesner R, Maroo S, Nokes T, Saunders D, Williams M. British Committee for Standards in Haematology. Guideline on the investigation, management and prevention of venous thrombosis in children. Br J Haematol. 2011; 154:196-207.

4. Ghosh PS, Ghosh D, Goldfarb J, Sabella C. Lateral sinus thrombosis associated with mastoiditis and otitis media in children: a retrospective chart review and review of the literature. J Child Neurol. 2011; 26:1000-1004.

5. Kaplan DM, Kraus M, Puterman M, Niv A, Leiberman A, Fliss DM. Otogenic lateral sinus thrombosis in children. Int J Pediatr Otorhinolaryngol. 1999; 49:177-183.

6. Funamura JL, Nguyen AT, Diaz RC. Otogenic lateral sinus thrombosis: case series and controversies. Int J Pediatr Otorhinolaryngol. 2014; 78:866-870.

7. Seven H, Ozbal AE, Turgut S. Management of otogenic lateral sinus thrombosis. Am J Otolaryngol. 2004; 25:329-333.

8. Sitton MS, Chun R. Pediatric otogenic lateral sinus thrombosis: role of anticoagulation and surgery. Int J Pediatr Otorhinolaryngol. 2012; 76:428-432.

9. Novoa E, Podvinec M, Angst R, Gurtler N. Paediatric otogenic lateral sinus thrombosis: therapeutic management, outcome and thrombophilic evaluation. Int J Pediatr Otorhinolaryngol. 2013; 77:996-1001.

## A63 Flat feet and knock knees: debunk a myth

### Alessandro Martinelli, Renato M Toniolo

#### Department of Traumatology, Bambino Gesù Pediatric Hospital, Rome, 00165, Italy

##### **Correspondence:** Alessandro Martinelli (alessandro.martinelli@opbg.net)

Flat feet and genu valgum are common orthopedic problems in children. The greater majority of cases are within physiologic limits at presentation and resolve normally without intervention. Angular deformities of the lower limbs are one of the biggest parental complaints in children treated in the Pediatric Orthopedic clinic, most of the time associated with deformities of the feet (flat feet). They are usually treated with observation and parental reassurance. However, a number of cases are related to pathologic entities due to both focal and systemic processes.

There is great controversy about the role that flat feet play in health, and disagreement on the indications for treatment. In asymptomatic flexible flat feet, the use of orthotics is generally over-rated and incorrect information is easily found online by parents. This sometimes leads to unnecessary treatment for children and increased social costs.

For both flat feet and valgus knee, obesity/overweight plays a strong role against the physiological evolution of the disturbance. It is mandatory that children maintain an active and healthy lifestyle as an increased BMI affect negatively the normal musculoskeletal development.

Knowledge of the natural history of knee alignment and feet conformation in the pediatric population can be a useful tool for the General Pediatrician in daily practice. It is important to know when a referral to a Pediatric Orthopedic Surgeon is indicated and which treatments may be offered to the patient.

**References**

1. Staheli LT. Evaluation of planovalgus foot deformities with special reference to the natural history. J Am Podiatr Med Assoc. 1987; 77:2–6.
2. Vanderwilde R, Staheli LT, Chew DE, et al. Measurements on radiographs of the foot in normal infants and children. J Bone Joint Surg Am. 1988; 70:407–15.
3. Sullivan JA. Pediatric flatfoot: evaluation and management. J Am Acad Orthop Surg. 199; 7:44 –53.
4. Harris EJ, Vanore JV, Thomas JL, Kravitz SR, Mendelson SA, Mendicino RW, Silvani SH, Gassen SC. Diagnosis and treatment of pediatric flatfoot. J Foot Ankle Surg. 2004; 43:341-73.
5. Dare DM, Dodwell ER. Pediatric flatfoot: cause, epidemiology, assessment, and treatment. Curr Opin Pediatr. 2014;26:93-100
6. Carr JB 2nd, Yang S, Lather LA. Pediatric pes planus: a state-of-the-art review. Pediatrics. 2016;137:e20151230
7. Rome K, Ashford RL, Evans A. Non-surgical interventions for paediatric pes planus. Cochrane Database Syst Rev. 2010. CD006311
8. White GR, Mencio GA. Genu valgum in children: diagnostic and therapeutic alternatives. J Am Acad Orthop Surg. 1995; 275-283.
9. Salenius P, Vankka E: The development of the tibiofemoral angle in children. J Bone Joint Surg Am 1975; 57:259-261.
10. Gettys FK, Jackson JB, Frick SL. Obesity in pediatric orthopaedics. Orthop Clin North Am. 2011 ;42:95-105
11. Bout-Tabaku S, Shults J, Zemel BS, Leonard MB, Berkowitz RI, Stettler N, Burnham JM. Obesity is associated with greater valgus knee alignment in pubertal children, and higher body mass index is associated with greater variability in knee alignment in girls. J Rheumatol. 2015; 42:126-33.
12. Heath CH, Staheli LT. Normal limits of knee angle in white children – genu varum and genu valgum. J Pediatr Orthop 1993; 13:259–262.

## A64 Urinary tract infections

### Laura Massella^1^, Giangiacomo Nicolini^2^

#### ^1^Nephrology Unit, Dpt. of Pediatric Subspecialties, Bambino Gesù Children’s Hospital, Rome, Italy; ^2^Pediatric and Neonatology Unit, San Martino Hospital, Belluno, Italy

##### **Correspondence:** Laura Massella (laura.massella@opbg.net)

Urinary tract infections are common in emergency room cases or primary care. The clinical picture, approach and outcome can vary widely depending on the site (low or high urinary tract), on associated morphological abnormalities of the kidney or of the urinary tract, on whether the patient is a newborn or a child, on whether the patient also suffers from a dysfunctional bladder, and on whether the patient is immunosuppressed or not. As a result, antibiotic treatment or need for prophylaxis depend on all the above-mentioned factors. Different guidelines on urinary tract infections in children have been published (National Institute of Clinical Excellence - NICE, American Academy of Pediatrics and the Italian guidelines on urinary tract infections). A European survey performed in 10 different countries showed that compliance with these guidelines is very limited.

This talk aims to review the literature, discuss the epidemiology of UTIs in children, antibiotic treatment and the role of antibiotic prophylaxis in reducing UTI-related renal scarring, and, finally, to provide some basic advice on evidence-based management of UTIs in children (treatment, prophylaxis, route of administration). Other aspects discussed include the main risk factors for antibiotic resistance in E. Coli (the germ most commonly involved in UTIs in children), and its implications for healthcare costs. In conclusion, even though a gold standard for the treatment of UTIs is not yet available, we now can manage UTIs to the best of our current knowledge.

## A65 Next generation sequencing: application and ethical issues in the newborn

### Marco Todeschini Premuda^1^, Luigi Memo^2^

#### ^1^Women's and Child's Health Department, University of Padua, 35100, Padua, Italy; ^2^Pediatrics Unit, San Martino Hospital, 32100, Belluno, Italy

##### **Correspondence:** Luigi Memo (luigi.memo@ulss.belluno.it)

The number of new genetic disorders with a molecular characterization is significantly growing. The poor specificity of clinical presentation and the difficulty obtaining appropriate biological samples are the peculiar diagnostic challenges of genetic disorders with neonatal onset.

The rapid development of next generation sequencing (NGS) is leading to considerable diagnostic progress. The application of NGS technology could have a key role in complex diagnosis issues since it potentially avoids ineffective treatments, justifies targeted therapy, limits complications with an overall reduction in hospitalisation and costs [1].

However, it raises several ethical issues related to incidental findings frequently associated with pathologies with adult onset or carrier status; identification of genetic variants of uncertain significance; privacy data protection [2,3]. Some countries already have a universal consensus about whether to reveal or not the incidental findings to the family. In Italy the patient or their tutor instead has the utmost discretion in this decision. Both options have limits. A complete and comprehensive informed consent on which genetic variants should be disclosed, could be a solution. In the most restrictive case, only those variants of immediate and crucial implication in therapy and management of the patient and their family would be notified. Therefore, the variants of uncertain significance, the predisposition to adult pathology or the carrier status would not be revealed to the patient or recorded in their medical records. However, the involvement of an independent ethical committee would be recommended in extraordinary cases. Furthermore, privacy and data protection implies an investment in the technologies for the anonymization and encrypting of all data.

The Italian Societies of Neonatology, Pediatrics, Human Genetics and Genetic Pediatric Diseases and Disability have issued an intersociety policy statement to define specific guidelines for the application of NGS technology in neonatal medicine. They suggest its use in critically ill newborns with an undefined clinical presentation compatible with a monogenic or genetically heterogeneous disease [4]. In these cases, an accurate genetic diagnosis is of utmost importance, since it indicates the appropriate surveillance and monitoring program, it predicts long-term complications, it provides the rationale for a targeted therapy and analyses the recurrence risk in prospective future reproduction choices.

The application of NGS technology avoids numerous invasive, non-specific and inconclusive tests and prevents possible complications caused by diagnostic delays. Moreover, the early diagnosis of a disease with a poor prognosis could significantly impact the decision process and management of the patient.

**References**

1. Berg JS, Agrawal PB, Bailey DB, Beggs AH, Brenner SE, Brower AM, Cakici JA, Ceyhan-Birsoy O, Chan K, Chen F, Currier RJ, Dukhovny D, Green RC, Harris-Wai J, Holm IA, Iglesias B, Joseph G, Kingsmore SF, Koenig BA, Kwok PY, Lantos J, Leeder SJ, Lewis MA, McGuire AL, Milko LV, Mooney SD, Parad RB, Pereira S, Petrikin J, Powell BC, Powell CM, Puck JM, Rehm HL, Risch N, Roche M, Shieh JT, Veeraraghavan N, Watson MS, Willig L, Yu TW, Urv T, Wise AL. Newborn sequencing in genomic medicine and public health. Pediatrics. 2017;139. pii: e20162252.
2. Botkin JR, Belmont JW, Berg JS, Berkman BE, Bombard Y, Holm IA, Levy HP, Ormond KE, Saal HM, Spinner NB, Wilfond BS, McInerney JD. Points to consider: ethical, legal, and psychosocial implications of genetic testing in children and adolescents. Am J Hum Genet. 2015;97:6-21
3. Committee on bioethics, Committee on genetics, and, the American College of Medical Genetics and, genomics social, ethical, and legal issues Committee. Ethical and policy issues in genetic testing and screening of children. Pediatrics. 2013,131:620-622.
4. Borghesi A, Mencarelli MA, Memo L, Ferrero GB, Bartuli A, Genuardi M, Stronati M, Villani A, Renieri A, Corsello G; their respective Scientific Societies. Intersociety policy statement on the use of whole exome sequencing in the critically ill newborn infant. Ital J Pediatr. 2017;43:100.

## A66 VRS bronchiolitis in the 2017-2018 season

### Michele Miraglia del Giudice, Maria Cristina Fedele

#### Department of woman, child, general and specialistic surgery, University of Campania “Luigi Vanvitelli”, Napoli, Italy

##### **Correspondence:** Michele Miraglia del Giudice (michele.miraglia@unicampania.it)

Viral bronchiolitis is a common clinical syndrome affecting infants and children under 2 years of age and it’s the main cause of respiratory illness requiring hospitalization at this age. During the last 3 season several Italian pediatric center enrolled infants with bronchiolitis (<12 months) in order to study the clinical trend of patients with bronchiolitis in different epidemic seasons based on the etiology and available therapies.

In Campania children hospitalized for bronchiolitis were collected during 2014-2017. The patients were divided into 2 groups: group A (353 cases admitted in the period 11/2014-03/2015 without using HFNC) and group B (642 cases admitted in the periods 11/2015-03/2016 and 11/2016-03/2017 using high-flow nasal cannula oxygen therapy (HFNC). Group A: 16 of 353 (4.5%) patients were transferred to intensive care, 11 (3.1%) were intubated, 5 (1.4%) died; Group B: 77 of 642 (12%) were treated with HFNC (67.5% RSV positive), 9 (1.4%) were transferred to intensive care, 2 (0.3%) were intubated, and no deaths were recorded. The average duration of the HFNC was 6.9 days (+/- 2.54). (Table 1)

In a Lazio pediatric hospital were enrolled infants admitted for VRS bronchiolitis during the last 3 seasons. In 2015/2016, 152 patients were collected, 137 (90,1%) RSV type A positive: 65 (42,7%) developed acute respiratory distress (ARDS), 65 (42,7%) were treated with oxygen, 6 (9,2%) with HFNC and 3 (4,6%) were transferred into a pediatric intensive unit.

In 2016/2017 season 101 patients were hospitalized for bronchiolitis. The distribution of Respiratory Syncytial virus changed, in fact 37 (36,6%) were positive for RSV type A, 63 for RSV type B (62,3%) and 1 for RSV type A+B (0,9%). Furthermore 52 children (51,4%) developed ARDS, 52 (51,4%) were treated with oxygen, 17 (32,6%) with HFNC and 9 (17,3%) were transferred into a pediatric intensive unit (PICU).

Finally in the last season (2017/2018) 171 children were collected, 99 (57,9%) were positive for RSV type A. The 81,2% of patients developed ARDS (139), 133 (77,7) were treated whit oxygen, 65 (46,7%) with HFNC and 27 (19,4%) transferred in PICU. In prematures (<37 weeks) the VRS type A prevails in all 3 seasons. (Table 2)

In conclusion it would be interesting to evaluate if this trend is common to other Italian regions.

**Table 1 (abstract A66).** Campania Children

	Group A (2014-2015)	Group B (2015_2017)
Hospitalized children	353	642
PICU	16	11
% (p=0,006)	4,6	1,7

**Table 2 (abstract A66).** Unpublished data of Ospedale Bambino Gesù – Roma

	2015/2016	2016/2017	2017/2018
Patients	152	101	171
Virus A	137	37	99
Virus B	14	63	67
Virus A+B	1	1	5
ARDS	65	52	133
Virus A	61	17	80
Virus B	4	35	56
Virus A+B	0	0	3
Oxygen	65	52	133
Virus A	61	17	78
Virus B	4	35	53
Virus A+B	0	0	2
HFNC	6	17	65
Virus A	6	8	45
Virus B	0	9	18
Virus A+B	0	0	2
PICU	3	9	27
Virus A	3	4	20
Virus B	0	5	6
Virus A+B	0	0	1

## A67 Age determination of unaccompanied minors in the transcultural approach

### Maria Chiara Monti (mkmonti@hotmail.com)

#### Association Centro Penc, Ethnopsychology Center, Palermo, Italy

**Background**

The increase in arrivals of young migrants in Europe has led migration authorities to request to health workers assistance in determining age of young people, whenever a significant reason arises to doubt the age declared.

In Italy, with the Zampa law, a holistic procedure is introduced to ascertain the minor age of young people who arrive alone and without documentations.

The presentation aims to address the controversial aspects of the assessment procedure to determine a person’age when it is uncertain; in particular, the part of the psycho-social interview will be addressed as a challenging process and complex task. This is often guided by the assumption that chronological age is a universal, culture-free, phenomenon, while it is not considered that many societies do not give a high importance to age calculation based on years, because they use different systems to define age membership.

**Material and method**

Through some case studies of the Ethnopsychology Center of Palermo, a model of transcultural approach to age assessment will be explained: the clinical interview is undertaken by the transcultural psychologist in the presence of the cultural mediator, and it takes into account the specific origin of person, his ethnic language and geopolitical context.

**Results**

From the experience of the Ethnopsychology Center of Palermo at least three types of situations emerged: the first one concerns young people who are not aware of their date of birth because in their country the most relevant aspect is the ritualistic passage and the age memebership, not the year of birth, and often they come from rural areas, far from big cities, where it is very rare to be registered at birth; the second group concerns young people who hide their age because they are afraid or under blackmail, like the victims of human trafficking, the victims of sexual exploitation and forced marriages; the third group is also relevant: young people who have been exposed to trauma, such as child maltreatment, modern slavery, sexual violence, imprisonment, they often can not tell about themself because unable to remember.

**Conclusions**

The case-by-case evaluation made through the experience of Ethnopsychology Center of Palermo showed us that the psycho-social interview must be conducted by a specialized transcultural team in identifying victims of trafficking, in collecting risk indicators and recognizing the signs of exposure to trauma. This allows the identification of vulnerable young people who require immediate access to assistance, support and protection.

## A68 Young child formulae and plant-based beverages

### Giuseppe Morino, Mirella Nicodemo, Maria Rita Spreghini

#### Paediatric Hospital Bambino Gesù - Rome – Italy

##### **Correspondence:** Giuseppe Morino (morino@opbg.net)

Infants have high nutritional needs for growth and development, with a lot of evidences supporting the importance of early life nutrition on long-term health outcomes: protein intakes in children from 1 to 3 years of age are excessive, while vitamin D, iron, ω-3 PUFA and iodine are below requirements.

Cow’s milk is commonly consumed among infants, contributing the highest percentage of energy intake; it is a rich source of Calcium, protein and fats, but it is low in certain micronutrients important for growth and development, such as Fe, vitamin D (8,9) and ω -3 essential fatty acids; however an excessive consumption of cow’s milk may cause a high protein intake.

The WHO suggests using fortified foods when children are not achieving adequate nutrient intake from their diet [1].

Young child formulae (YCF), term who included Toddler’s milk, growing up milk or formula for young children, are milk-based drinks or plant protein-based formulae intended to partially satisfy the nutritional requirements of young children aged 1 to 3 years, in detail for Fe, vitamin D and ω-3 essential fatty acids. The term “growing up” should not be used because it implies a specific impact on growth [2].

A late study evaluated who micronutrient-fortified YCF preserves iron status and improves vitamin D status in healthy young children [3].

Recently, a systematic review has estimated the role of fortified milk on growth and other biochemical markers. Fortified milk had minimal effects on weight gain compared with control milk. The risk of anaemia was reduced in fortified milk groups compared with control groups. There were no significant effects on height gain, changes in body composition or Hb concentration [4].

In recent years, the intake of plant-based beverages (PBBs) in the first years of life has increased. The main reasons for this change are preference for plant foods, aversion to the use of cow’s milk, and prevention or treatment of cow’s milk allergy, as part of strict vegetarian diets or as a consequence of the advice of *alternative medicine practitioners*. In the first years of life the exclusive consumption of mainly soy, rice, almond or oat PBBs results in nutritional risks [5].

Based on available evidence, YCF can be used as part of a healthy diet for children aged 1 to 3 years.

**References**

1. World Health Organization. Essential Nutrition Actions: Improving Maternal, Newborn, Infant and Young Child Health and Nutrition. Geneva: WHO. 2013.
2. Hojsak I, Bronsky J, Campoy C, Domellöf M, Embleton N, Fidler Mis N, Hulst J, Indrio F, Lapillonne A, Mølgaard C, Vora R, Fewtrell M. Young child formula : A Position Paper by the ESPGHAN Committee on Nutrition. J Pediatr Gastroenterol Nutr. 2018;66:177-185.
3. Akkermans MD, Eussen SR, van der Horst-Graat JM, van Elburg RM, van Goudoever JB, Brus F. A micronutrient-fortified young-child formula improves the iron and vitamin D status of healthy young European children: a randomized, double-blind controlled trial. Am J Clin Nutr. 2017; 105:391-399.
4. Matsuyama M, Harb T, David M, Davies PS, Hill RJ. Effect of fortified milk on growth and nutritional status in young children: a systematic review and meta-analysis. Public Health Nutr. 2017; 20:1214-1225.
5. Vitoria I. The nutritional limitations of plant-based beverages in infancy and childhood. Nutr Hosp. 2017; 34:1205-1214.

## A69 Clinical results after 15 years of home telemonitoring in cystic fibrosis

### Fabrizio Murgia (telemedicina@opbg.net)

#### Department of Specialist Pediatrics- Integrated Home Care for Chronic Diseases-Bambino Gesù Pediatric Hospital – Piazza S. Onofrio, 4-00165 Rome, Italy

The natural history of Cystic Fibrosis (CF) is characterized by recurrent episodes of respiratory infection causing a progressive pulmonary damage, with decay of long-term lung function leading to death. In CF patients, spirometry shows a 2% reduction every year of Forced Expiratory Volume in the first second (FEV1) over time. In case of pulmonary infection, an early antibiotic treatment helps to prevent more serious complications, limiting consequently the long-term pulmonary damage. Since 2001, in CF Centre of the Pediatric Hospital Bambino Gesù in Rome, we used Telemedicine (TM) to facilitate the home follow-up of patients. Fev1 was monitored at home, in the aim to early recognize pulmonary relapses. The project has involved 78 patients affected by CF, followed at our Unit with telehomecare (THC) in addition to the usual therapeutic protocol, for a period of 15 years. The balance of enrolment showed a drop-out of 33%, the main cause was poor adherence (65%). We used various and different equipment in this period, also following the progress of technology in this field. To investigate the possible role of THC in follow-up of CF at home, we monitored the activities of THC treated patients from 2011 to 2014 toward a similar control group. The design of the study was case control - open label trial. THC was applied in addition to the standard therapeutic protocol. The endpoint was the annual mean Fev1 over time. We found that THC patients improved their FEV1 values with a significantly better trend than the one reported in the control group. Later, we extended the observational period, by monitoring the activities of same patients until 2016. Our results showed once more that THC treated patients improved their FEV1 values with a trend significantly better than the one reported by controls. The possible meaning of the results is discussed. We conclude that the application of telemonitoring in CF, while not able to treat the disease radically, could reduce, through a better identification of relapses, the lung damage secondary to respiratory exacerbations, allowing a better respiratory stability and therefore a better quality of life.

## A70 Local vaccine reactions: how to cure them

### Luciana Nicolosi (luciana.nicolosi@opbg.net)

#### Pediatric and Infectious Diseases Unit, Dipartimento Pediatrico Universitario Ospedaliero, Bambino Gesù Children’s Hospital, IRCCS, Rome, Italy

**Background**

Vaccination has strikingly reduced morbidity and mortality due to childhood infectious diseases in developed countries and also protected infants too young to be vaccinated. Nevertheless, parents may be still hesitant to get their children vaccinated due to lack of knowledge of the seriousness of the disease, skepticism about vaccination benefits, and fear of adverse events following immunization. Declining vaccination rates could facilitate the spread of illness and death from vaccine preventable diseases and could increase costs for society, both as direct health care costs and indirectly through lost productivity. However, vaccinations are threatened by their own success: the more the incidence of potentially devastating diseases decreases, thanks to the success of vaccination programs, the more public attention shifts towards real or alleged “side effects” of vaccines.

**Materials and methods**

We evaluated all children who have been vaccinated at the Vaccine Unit of Bambino Gesù Children’s Hospital from 10 September 2014, until 18 February 2016. Out of a total of 1367 enrolled subjects, 76 children (6%) presented with a previous history of one or more VAEs (Vaccine Adverse Events); 31 (41%) of them, described a VAE of suspected allergic origin (i.e., urticaria/angioedema, anaphylaxis). In case of more than one VAEs, these were classified as “main event” and “secondary event”, depending on the severity of reported symptoms. The decision about whether to continue immunisations was made evaluating different factors: absence of specific contraindications, parents’ counseling, adequate hospital setting, choice of an appropriate and individualized schedule.

**Results**

None of the 76 children vaccinated after VAEs presented further side effects.

**Conclusions**

VAEs occur very rarely. A history of VAE does not necessarily represent a contraindication to re-vaccination, and clinicians must be encouraged to take advantage of algorithms for VAEs assessment, to evaluate the risk of recurrence. The real risk of a VAE is mostly associated with serious allergic reactions (IgE‐mediated anaphylaxis) and parents should be aware of this information, so that the widespread fear of VAE recurrence can be limited. Indeed, this type of concern represents one of the main reasons for vaccination hesitancy, which leads to incomplete vaccination schedules.

**Acknowledgements:** I thank all my colleagues and nurses in particular Dr. G. Castelli Gattinara, Dr. E. Bellelli, Dr. D.F. Angelone, Dr. V. Santilli, A. Serpi, L. Varanese, and R. Montanaro

who provided insight and expertise that greatly assisted this research.

## A71 The workflow in a home telemonitoring project: the role of the nurse

### Paglia Clarissa, Murgia Fabrizio, Bella Sergio

#### Cystic Fibrosis Unit, Bambino Gesù Children’s Hospital Rome, Roma, Italy

##### **Correspondence:** Paglia Clarissa (clarissa.paglia@opbg.net)

Cystic Fibrosis (CF) is the most common genetic disease with an ominous outcome that affects the Caucasian population. Classically, the CF is known as a respiratory disease, where it causes repeated infections and progressive bronchiectasis formations until the development of respiratory failure.

Respiratory exacerbations are a critical event for the CF patient, leading to a deterioration in quality of life, increasing lung damage, costs and intensity of care. Continuous monitoring of the patient's clinical situation helps to prevent and / or minimize exacerbations, through a timely recognition of symptoms of exacerbation, with positive consequences for the patient's life expectancy and the rationalization of hospital admissions.

Since 2001, at the Center for Cystic Fibrosis of the Bambino Gesù Children’s Hospital I.R.C.C.S. in Rome, we apply the telemonitoring of respiratory parameters in the follow-up of patients at home. From February 2018 a nurse assists the doctor in the management of the service.

The responsible nurse collects, analyzes, evaluates the transmissions of spirometric data such as FEV1, FVC, PEF, FEF2575, which the patients perform weekly from home and send to a dedicated platform. Together with other parameters (SpO2, nocturnal saturimetry, heart rate, PA, blood glucose and body weight) makes an assessment of the patient's clinical status, which also occurs through the responses of the same to a questionnaire on subjective symptoms (shortness of breath for small 'sputum, increased sputum, tiredness on waking, coughing, frequent awakenings, chest tightness, fatigue, daytime sleepiness).

The interview with the doctor allows the nurse to discuss "the assistance plan", on the basis of which they decide the therapeutic interventions that will be communicated to the patient, or to the parents, through a telephone interview. The virtual contact with the patients is the fulcrum of the intervention of the responsible nurse, who has the possibility to evaluate their care needs, adherence to basic therapy and physiotherapy, to request further data transmissions or the call to the hospital for assessments and treatment under Ambulatory, Day Hospital or Ordinary Hospitalization.

The nurse is also responsible for monitoring the proper functioning of the instruments used by the patients, dealing with the calibration of the spirometer, the updating of the indicators on which the measurements of FEV1 (weight and height) and of the instrument's software are based, interfacing with the company responsible for the assistance of the same, where the patient had problems of a purely technical nature.

## A72 Helmet CPAP and Non-invasive Ventilation in pediatric patients with acute respiratory failure: single hospital experience

### Daniela Perrotta, Matteo Di Nardo, Marco Marano, Francesca Stoppa, Corrado Cecchetti

#### Intensive Care Unit, Emergency Department, Bambino Gesù Pediatric Hospital, Piazza Sant'Onofrio 4, Rome, Italy

##### **Correspondence:** Daniela Perrotta (daniela.perrotta@opbg.net)

**Background**

Acute Respiratory Failure (ARF) and its more severe form, Acute Respiratory Distress Syndrome (ARDS), are life-threatening condition of acute pulmonary inflammation and hypoxia, resulting in respiratory failure.[1] Helmet CPAP and NIV are helpful to reduce the need and complications from intubation and invasive mechanical ventilation, but a very cautious approach is needed requiring expertise, a protect environment, careful patient’s selection and a low threshold for intubation. [2] In addition, NIV has also been recommended in hypoxemic ARF in immune-compromised patients. [3]

**Objective**

Evaluate the safety of early administration, outside the Pediatric Intensive Care Unit, of Helmet CPAP and HFNC in children with hypoxic ARF.

**Subjects and Methods**

22 patients (median age 8,38), with different hemato-oncological diseases from August to December 2017 were enrolled. We evaluated the state of disease, the eventuality of previous HSCT and the surviving of the patients.

**Results**

7 patients were treated at the “Department of Hematology/Oncology and Stem Cell Transplantation” with HFNC, 1 with Helmet CPAP and 4 received both the treatments. For none of the patients was necessary intubation and invasive mechanical ventilation and none presented pneumothorax.

**Conclusions**

ARF is a common cause of PICU admission among pediatric hemato-oncology patients. [4] With our study we found that an early interdisciplinary management by oncologists and intensivists with an advanced respiratory support such HFNC or Helmet CPAP could reduce admissions of these patients in PICU and can be safely performed outside the Pediatric Intensive Care Unit.

**References**

1. Santschi M, Jouvet P, Leclerc F Acute lung injury in children:therapeutic practice and feasibility of international clinical trials.Pediatr Crit Care Med.2010;11:681-689.

2. Kneyber M, de Luca D, Calderini E Recommendations for mechanical ventilation of critically ill children from the Paediatric Mechanical Ventilation Consensus Conference (PEMVECC) Intensive Cre Med 2017; 43:1764-1780.

3. Antonelli M, Conti G, Rocco M, Bufi M, De Blasi RA, Vivino G et al. A comparison of noninvasive positive-pressure ventilation and conventional mechanical ventilation in patients with acute respiratory failure. N Engl J Med 1998; 339: 429–435.

4. Piastra M, Fognani G, Franceschi A. Pediatric intensive care unit admission criteria for haemato-oncological patients: a basis for clinical guidelines implementation. Pediatr Rep. 2011;3:e13.

## A73 Multidisciplinary approach to nasal functions. The dentist

### Antonella Polimeni, Valeria Luzzi

#### Department of Oral and Maxillo-facial Sciences, “Sapienza” University of Rome, Roma, Italy

##### **Correspondence:** Antonella Polimeni (antonella.polimeni@uniroma1.it)

The last half century saw a marked increase in the incidence of respiratory diseases, especially allergic rhinitis among pediatric subjects. This is probably due to multiple factors, among which an increasingly worrying environmental and atmospheric pollution stand out as they favor an early exposure of children to various types of allergens, with the consequent sensitization of their airways.

Allergic rhinitis (AR) is a public health problem that affects adults, adolescents, and children. Epidemiological studies indicated that in developed and industrialized countries the prevalence of AR increased progressively over the last three decades. AR currently affects up to 40% of the world population, with prevalence variations between adults and children and between different countries. It is estimated that in Italy the prevalence of AR in individuals aged 6 to 14 years is 33-35%, with an incidence that in the last five years showed an increase of 5%.

Airway obstruction associated to AR is a risk factor for the development of malocclusion. According to many authors, any obstacle to the normal nasal airflow leads to non-physiological oral breathing, which determines postural changes such as open lips, low or frontal tongue, and lower posterior rotation of the jaw. This involves a modification of the pressure that the soft tissues exert on the teeth and on the facial bones, creating a situation of disequilibrium that negatively affects the normal growth of these structures. Although most authors recognize this correlation, other scholars do not associate the respiratory functions with dentofacial development, arguing that it is not possible to establish a direct relationship between the two factors. They emphasized the importance played by genetics in the manifestation of craniofacial alterations, emphasizing for example how "longface" subjects have a greater tendency to develop oral respiration compared to normal individuals. The therapeutic approach to these malocclusions is multidisciplinary and requires the intervention of the allergist and of the otolaryngologist together with that of the pediatric dentist and of the orthodontist in order to implement combined therapeutic protocols with the aim of restoring the airway patency through orthodontic-orthopedic therapy and myofunctional therapy.

## A74 Obstructive sleep apnea: the role of the orthodontist

### Antonella Polimeni, Valeria Luzzi

#### Department of Oral and Maxillo-facial Sciences, “Sapienza” University of Rome, Roma, Italy

##### **Correspondence:** Antonella Polimeni (antonella.polimeni@uniroma1.it)

Sleep disordered breathing (SDB) in children represents a broad spectrum of respiratory disorders characterized by partial or complete obstruction of the upper airway. Primary snoring (PS) identifies its mildest clinical manifestation. Evolving through progressively more severe forms, it can lead to the complete obstruction of the upper airways with cessation of the airflow, known as Obstructive Sleep Apnea Syndrome (OSAS). Prevalence of OSAS in the pediatric age varies between 1 and 3% and its diagnostic approach is multidisciplinary. Hence the importance of the pediatric dentist and of the orthodontist, professional figures who can detect early signs of the disease and act as a 'sentinel' for immediate referral to the otolaryngology specialist.

Orthodontic treatment aims at reducing the severity of the OSAS through orthopedic expansion of the maxilla and mandibular advancement, with the goal of increasing the airspace and consequently improving the airflow. Rapid palatal expanders are successfully used in OSAS. Mandibular Advancement Devices (MAD), designed to promote anterior sliding of the mandible, also reduce airway collapse.

Therefore, the orthodontist plays a pivotal role in the orthodontic treatment phase as orthopedic expansion of the maxilla and/or mandibular advancement contribute to the reduction of the severity of OSAS in children.

## A75 The child with polyuria and polydipsia: how to make the diagnosis

### Ivana Rabbone (Ivana.rabbone@unito.it)

#### Pediatric Diabetes Centre, Department of Pediatric, Regina Margherita Children Hospital, 10126 Turin, Italy

Polyuria is the passage of excessive quantity of urine and it implies water or solute diuresis of at least 2.5–3 L/day or urine volume of more than 40 mL/kg/day. Polyuria is usually associated with polydipsia. Polydipsia is defined as water intake of more than 100 mL/kg/day (6 L/day). Routinely, we tend to ascribe it to diabetes mellitus (DM), but there are many causes to be considered in the differential diagnosis and a thorough work-up will be needed in most cases.

There are four mechanisms, which can cause polyuria. One or more of these will be operating:

1. Increased intake of fluids as in psychogenic causes, stress and anxiety.

2. Increased glomerular filtration rate as in hyperthyroidism, fever, hypermetabolic states.

3. Increased output of solutes as occurs in DM, hyperthyroidism, hyperparathyroidism, use of diuretics (which present more solute at the distal convoluted tubule)

4. Inability of the kidney to reabsorb water in distal convoluted tubule as in diabetes insipidus , nephrogenic diabtes insipidus, drugs and chronic renal failure.

If the 24-hour urine volume is more than 3 liters, then we need to measure the urine osmolality. If the urine osmolality is more than 300 mOsm/ kg, it indicates solute diuresis and the underlying cause will be either DM or cronic kidney desease. Evaluation must be directed at these conditions.

As concern DM diagnosis, if symptoms are present, urinary ‘dipstick’ testing for glycosuria and ketonuria, or measurement of glucose and ketones using a bedside glucometer, provides a simple and sensitive screening tool. [1] If the blood glucose level is elevated, then prompt referral to a center with experience inmanaging children with diabetes is essential, because ketoacidosis can evolve rapidly.

Scenarios where the diagnosis of diabetes may be unclear include: absence of symptoms, for example, hyperglycemia detected incidentally or in children participatingin screening studies; presence of mild/atypical symptoms of diabetes; hyperglycemia detected under conditions of acute infective, traumatic, circulatory, or other stress, which may be transitory and should not be regarded as diagnostic of diabetes; in addition, the presentation of a familial form of mild diabetes during adolescence should raise the suspicion of monogenic diabetes, which accounts for 1–6% of pediatric diabetes cases [2] .

The differentiation between type 1, type 2, monogenic, and other forms of diabetes has important implications for both therapeutic decisions and educational approaches.

**References**

18. Craig ME, Jefferies C, Dabelea D, Balde N, Seth A, Donaghue KC. Definition, epidemiology, and classification of diabetes in children and adolescents. Pediatr Diabetes. 2014:15:4–17.
19. Delvecchio M, Mozzillo E, Salzano G, Iafusco D, Frontino G, Patera PI et al. Monogenic diabetes accounts for 6.3% of cases referred to 15 italian pediatric diabetes centers during 2007 to 2012. J Clin Endocrinol Metab. 2017;102:1826–1834.

## A76 Rights and duties of the doctor undergoing specialised medical training

### Francesco Rampulla (studipolgiur@unipv.it)

#### Professor of Administrative Law, University of Pavia, Pavia, Italy

The description of the identification of the needs, the organization and the duties of the Medical Specialities, as well as the legal reconstruction of the contract of doctors undergoing specialised training constitutes the first part of this report.

Then, the duties of doctor undergoing specialised training (attending classes with diligence, participating in practical activities prescribed by the related Decree of Ministers), their responsibilities and the tutors’ ones will be described.

The rights of the doctors undergoing specialised training (such as weekly rests and holidays), derived on one hand from constitutional rules, legislative dispositions (such as puerperium and maternity leave, mandatory rests after work shifts) and contractual dispositions (such as periods of suspension of the training and periods of break of 40 days, recoverable), and on the other from the equalisation to doctors already framed into the medical organization (as the right to have appropriate clothes, parking and canteen services) will be analysed.

Finally, the relations with hospitals regarding the duties on insurances, especially in the light of the Act 24/2017, and on the logistics and services, will be highlighted.

The conclusions underline that an update and completion of both the legislation and the standard contract model are desirable.

## A77 Immunodeficiency and autoimmunity: one or two opposing diseases?

### Silvia Ricci^1^, Clementina Canessa^1^, Francesca Lippi^1^, Ilaria Pagnini^2^, Massimo Resti^3^, Chiara Azzari^1^

#### ^1^Paediatric Immunology Department, Meyer Children’s Hospital, Florence, Italy; ^2^Paediatric Rheumatology Department Meyer Children’s Hospital, Florence, Italy; ^3^Paediatric Department, Meyer Children’s Hospital, Florence, Italy

##### **Correspondence:** Silvia Ricci (silvia.ricci@meyer.it)

Immunodeficiency and autoimmunity were considered to be mutually exclusive conditions. When considering patient with Primary ImmunoDeficiency (PID) we know that he is predisposed to recurrent and severe infections (hallmark of immunodeficiency). However, increased understanding of the complex immune regulatory and signaling mechanisms involved in immune system, is revealing the complex relationships between PIDs and autoimmune diseases. Many researchers have studied the pathogenesis of immune dysregulation can lead to autoimmunity/inflammation in the context of PIDs. These mechanisms include impaired production of flogistic citokynes (type I interferon or IL-1), defective negative selection of self-reactive T (APECED), defective editing of the B-cell receptor in the periphery, defective peripheral (self)-antigen–induced cell death (ALPS), gain of function of B- or T-cell activation/effector molecules, defective regulatory T cells (IPEX and IPEX like syndromes), homeostatic expansion of self-reactive T lymphocytes. Recently, Fisher et al. demonstrated that autoimmune and auto-inflammatory diseases are much more frequent by a factor of at least 10 in the cohort of patients with PID than in the general population. The spectrum of autoimmunity in PID is broad, but the increased risk is extremely high (by a factor of 120) for autoimmune cytopenia (anemia and thrombocytopenia), gastrointestinal disease, or arthritis. These data suggest that patients with autoimmune anemia, thrombocytopenia, or both should be screened for PIDs during childhood. Moreover, the presence of autoimmunity or autoinflammation worsens the outcome for patients with PID, complicating the clinician therapeutic strategies. To find a balance among immunosuppressive therapy in patients and their susceptibility to infections is a clinical challenge. The improving knowledge of various molecular and cellular pathologic mechanisms, causing autoimmunity/inflammation in PID patients, is really important in order to obtain personalized and safe therapies that could potentially treat immunodeficiency and autoimmunity at the same time.

**References**

1. Fischer A, Provot J, Jais JP, Alcais A, Mahlaoui N, members of the CEREDIH French PID study group. Autoimmune and inflammatory manifestations occur frequently in patients with primary immunodeficiencies. J Allergy Clin Immunol. 2017;140:1388-1393.
2. Rae W, Ward D, Mattocks CJ, Gao Y, Pengelly RJ, Patel SV, Ennis S, Faust SN, Williams AP. Autoimmunity/inflammation in a monogenic primary immunodeficiency cohort. Clin Transl Immunology. 2017;6:155.
3. Schmidt RE, B Grimbacher B, Witte T. Autoimmunity and primary immunodeficiency: two sides of the same coin? Nat Rev Rheumatol. 2017;14:7-18.
4. Walter JE, Farmer JR, Foldvari Z, Torgerson TR, Cooper MA. Mechanism-based strategies for the management of autoimmunity and immune dysregulation in primary immunodeficiencies. J Allergy Clin Immunol Pract. 2016;4:1089-1100.

## A78 The treatment of childhood cancer: the immunotherapy rises the challenge

### Erica Brivio, Antonella Colombini, Fabiola Dell’Acqua, Carmelo Rizzari

#### Pediatric Hematology Oncology Unit, Department of Pediatrics, University of Milano-Bicocca, ASST Monza, Italy

##### **Correspondence:** Carmelo Rizzari (carmelo.rizzari@gmail.com)

Despite accounting for only 1% of all cancers, childhood cancer is the leading disease-related cause of death in children. Even so, the five-year survival has improved greatly in the last two-three decades, increasing up to 84% for tumours diagnosed in 2005–11. Currently, despite the extensive implementation of clinical trials and multidisciplinary approaches, survival outcomes for childhood malignancies have plateaued and a number of survivors live with long-term disabling sequelae through adulthood. The introduction of novel therapeutic agents, for relapsed patients and even into frontline treatments, could be the breakthrough needed in this landscape. [1]

Novel anticancer agents comprise different drug classes, including immune-checkpoint inhibitors (e.g. anti-PDL1 for solid tumours and lymphomas), monoclonal antibodies (e.g. blinatumomab for acute lymphoblastic leukaemia) or the newest gene-therapy with CAR-T cells. [1-3] These new agents act against the tumour together with the patient’s immune system, keeping or enhancing its innate anti-cancer activity. This emerging approach is part of the precision medicine process; in fact, targeting different pathways specific for each kind of tumour, can help to achieve remission in larger cohorts of patients and, at the same time, can theoretically reduce the burden of toxic effect mediated by chemotherapy against healthy tissues. [4] Results are particularly encouraging in the field of acute lymphoblastic leukemia wherein fast-track approvals have been already granted by competent authorities. Several additional trials are attempting to get similar results in acute myeloid leukemia, in chronic leukemias and solid tumors. As often happens with new therapies also risks and side effects are emerging and could contribute to hamper the potential success of these agents when applied in the clinical practice such as the cytokine release syndrome and B-cell aplasia related to CAR-T cells therapy. Long-term consequences of these iatrogenic syndromes cannot be currently predicted. In addition, most molecular targeted agents provide only partial inhibition of signaling pathways, with incomplete tumour regression. A combination of molecular targeted agents may inhibit alternative pathways, but the extent of signalling plasticity and the Darwinian evolution of tumours could limit the effectiveness of this approach. Another issue is the cost of such precise medicine: new drugs are marketed at ever-increasing price and molecular analysis of tumour samples is expensive and not always efficient. [5]

Paediatric oncology, characterized by rare entities and small number of patients with specific pharmacokinetic and pharmacodynamic characteristics, can take advantage from these approaches only through well-designed collaborative and international programs. [6]

**References**

1. Maude SL, Laetsch TW, Buechner J, Rives S, Boyer M, Bittencourt H, Bader P, Verneris MR, Stefanski HE, Myers GD, Qayed M, De Moerloose B, Hiramatsu H, Schlis K, Davis KL, Martin PL, Nemecek ER, Yanik GA, Peters C, Baruchel A, Boissel N, Mechinaud F, Balduzzi A, Krueger J, June CH, Levine BL, Wood P, Taran T, Leung M, Mueller KT, Zhang Y, Sen K, Lebwohl D, Pulsipher MA, Grupp SA. Tisagenlecleucel in Children and Young Adults with B-Cell Lymphoblastic Leukemia. N Engl J Med. 2018;378:439-48.
2. Von Stackelberg A, Locatelli F, Zugmaier G, Handgretinger R, Trippett TM, Rizzari C, Bader P, O'Brien MM, Brethon B, Bhojwani D, Schlegel PG, Borkhardt A, Rheingold SR, Cooper TM, Zwaan CM, Barnette P, Messina C, Michel G, DuBois SG, Hu K, Zhu M, Whitlock JA, Gore L. Phase I/Phase II Study of Blinatumomab in Pediatric Patients With Relapsed/Refractory Acute Lymphoblastic Leukemia. J Clin Oncol. 2016;34:4381-9.
3. Wagner LM, Adams VR. Targeting the PD-1 pathway in pediatric solid tumors and brain tumors. Onco Targets Ther. 2017;10:2097-106.
4. Mody RJ, Prensner JR, Everett J, Parsons DW, Chinnaiyan AM. Precision medicine in pediatric oncology: Lessons learned and next steps. Pediatr Blood Cancer. 2017;64.
5. Tannock IF, Hickman JA. Limits to Personalized Cancer Medicine. N Engl J Med. 2016;375:1289-94.
6. Moreno L, Pearson ADJ, Paoletti X, Jimenez I, Geoerger B, Kearns PR, Zwaan CM, Doz F, Baruchel A, Vormoor J, Casanova M, Pfister SM, Morland B, Vassal G. Early phase clinical trials of anticancer agents in children and adolescents - an ITCC perspective. Nat Rev Clin Oncol. 2017;14:497-507.

## A79 Dysvitaminosis in pediatric gastrointestinal diseases

### Valeria Dipasquale, Claudio Romano

#### Department of Human Pathology in Adulthood and Childhood "G. Barresi", University of Messina, Messina, 98122, Italy

##### **Correspondence:** Claudio Romano (romanoc@unime.it)

Vitamins are chemically unrelated families of organic compounds that need to be taken through diet because humans cannot synthesize them in adequate quantities. Each vitamin has unique functions in the body, including hormone regulation, cell proliferation, tissue growth and differentiation, and antioxidant effects; vitamins also serve as cofactors for multiple metabolic pathways [1]. Vitamins A, D, E, and K are lipid-soluble, whereas vitamin B complexes and vitamin C are water-soluble. A large amount of literature data supports the association of childhood gastrointestinal diseases and dysvitaminosis. Multiple micronutrient deficiencies have been described as either cause or effect of short bowel syndrome (SBS), which is the predominant underlying cause of intestinal failure (IF) usually after intestinal resection for congenital (intestinal atresia, malrotation with volvulus) or acquired (necrotizing enterocolitis, vascular thrombosis, or trauma) disorders [2-4]. Terminal ileal resection with gastric acid blockade significantly rise the risk of hypovitaminosis B12 in children with SBS. Another condition associated to dysvitaminosis is the transition, in SBS, from parenteral to full enteral nutrition [4,5]. Maternal/neonatal hypovitaminosis D can be associated necrotizing enterocolitis (NEC) risk in premature infants supporting evidence that vitamin D plays a very important role in the intestinal homeostasis [6]. Hypovitaminosis D is highly prevalent (up to 35%) among pediatric patients with inflammatory bowel disease (IBD) [7]. Although low bone mineral density has not been attributed to dietary vitamin D deficiency, but rather to the inflammatory processes, attaining adequate vitamin D status in children with IBD is recommended [7]. Notably, the evidence in adult population suggests that vitamin D deficiency is independently associated with lower health-related quality of life and greater disease activity in patients with Crohn’s disease (CD), but not those with ulcerative colitis (UC) [8]. Children with chronic diarrhea may present with deficiency of selected micronutrients such as vitamin A, and folic acid. Historically, celiac disease has been associated with many micronutrient deficiencies, particularly the fat-soluble vitamins (A, D, E, and K), since in the past celiac disease was usually diagnosed late following a period of prolonged diarrhea. Many studies have demonstrated that fat-soluble vitamin deficiencies are uncommon in modern-era pediatric celiac disease, probably because of earlier diagnosis, the routine use of vitamin supplements or fortification of cereal products with vitamins [9]. For all of the adult patients with a new diagnosis, the expert opinion recommends checking for vitamin D, vitamin A, zinc, copper, folic acid, and ferritin [10]. Routine measuring of fat-soluble vitamins levels may not be necessary in children and is not clearly recommended.

**References**

1. Lauer B, Spector N. Vitamins. Pediatr Rev. 2012;33:339-51.

2. Yang CF, Duro D, Zurakowski D, Lee M, Jaksic T, Duggan C. High prevalence of multiple micronutrient deficiencies in children with intestinal failure: a longitudinal study. J Pediatr. 2011;159:39-44.

3. Ubesie AC, Heubi JE, Kocoshis SA, Henderson CJ, Mezoff AG, Rao MB, Cole CR. Vitamin D deficiency and low bone mineral density in pediatric and young adult intestinal failure. J Pediatr Gastroenterol Nutr. 2013;57:372-6.

4. Ubesie AC, Kocoshis SA, Mezoff AG, Henderson CJ, Helmrath MA, Cole CR. Multiple micronutrient deficiencies among patients with intestinal failure during and after transition to enteral nutrition. J Pediatr. 2013;163:1692-6.

5. Stabler SP. Clinical practice. Vitamin B12 deficiency. N Engl J Med. 2013;368:149-60.

6. Cetinkaya M, Erener-Ercan T, Kalayci-Oral T, Babayiğit A, Cebeci B, Semerci SY, Buyukkale G. Maternal/neonatal vitamin D deficiency: a new risk factor for necrotizing enterocolitis in preterm infants? J Perinatol. 2017;37:673–678.

7. Pappa HM, Gordon cM, Saslowsky TM, Zholudev A, Horr B, Shih M et al. Vitamin D status in children and young adults with inflammatory bowel disease. Pediatrics. 2006;118:1950–61.

8. Ulitsky A, Ananthakrishnan AN, Naik A, Skaros S, Zadvornova Y, Binion DG, Issa M. Vitamin D deficiency in patients with inflammatory bowel disease association with disease activity and quality of life. JPEN J Parenter Enteral Nutr. 2011;35:308–16.

9. Imam MH, Ghazzawi Y, Murray JA, Absah I. Is it necessary to assess for fat-soluble vitamin deficiencies in pediatric patients with newly diagnosed celiac disease? J Pediatr Gastroenterol Nutr. 2014;59:225-8.

10. Rubio-Tapia A, Hill ID, Kelly CP, Calderwood AH, Murray JA. Diagnosis and management of celiac disease. Am J Gastroenterol. 2013; 108:656–676.

## A80 From a Journal Club activity to the “Pediatric Newsletter”: how to search for clinical evidence for practice and how to critically assess it

### Luca Ronfani^1^, Roberto Buzzetti ^2^

#### ^1^Clinical Epidemiology and Public Health Research Unit, Institute for Maternal and Child Health IRCCS Burlo Garofolo, Trieste, 34137, Italy; ^2^Epidemiologist, Bergamo, Italy

##### **Correspondence:** Luca Ronfani (luca.ronfani@burlo.trieste.it)

**Background**

Reading scientific articles is essential for medical update but is also a time-consuming activity since scientific literature is constantly growing. The Pediatric Newsletter was founded in 2004 to ensure constant monitoring of core pediatric literature and sharing, among primary care and hospital pediatricians, of structured syntheses of relevant articles selected based on their transferability in daily clinical practice.

**Material and methods**

a) Searching for evidence: nowadays, several potential sources are available for physicians to carry out exhaustive literature searches (i.e., databases of citations or bibliographic summaries such as MEDLINE, EMBASE, CINAHL, the Cochrane Library, the main guidelines databases, etc). Our reading groups, consisting mainly of family pediatricians, decided to follow a different strategy, reviewing, on a monthly basis, the tables of contents of the main pediatric and general medical journals (amongst these BMJ, the Lancet, the New England Journal of Medicine, JAMA, Pediatrics, Journal of Pediatrics, Archive of Disease in Childhood, JAMA pediatrics, BMC pediatrics) and of the main database of systematic reviews (the Cochrane Library). The potentially relevant articles identified through this screening, are read in full text, evaluated for their methodological quality, and discussed during the monthly meetings of the Journal Clubs, in a continuous training activity.

b) Critically appraising the evidence: for relevant articles, a standard form is produced which includes the following fields: a short structured abstract; a “quick and dirty” literature review, which helps place the new study within the evidence already available; the definition of the possible innovative contribution of the new study; the analysis of the methodological quality of the study (i.e., the internal validity, outcomes considered, etc); the transferability and the possible impact on clinical practice (external validity). A general meeting of all the reading groups is held annually to discuss methodological aspects, emerging problems, future perspectives, possibilities of improvement.

**Results**

In 14 years, 165 pediatricians from several cities in Italy took part in Journal Club activities, reviewing over 400 articles, 140 of which assessed/summarized using the standard structured form. In the last 4 years alone, 110 pediatricians, organized in 12 local reading groups, have presented 115 structured forms.

**Conclusions**

The “Pediatric Newsletter” is a valid tool for professional updating and continuous training, it is sustainable over time and easily exported locally.

## A81 Atypical infantile presentation of hypertrophic cardiomyopathy (HCM) detected incidentally by point of care ultrasound (POCUS) in pediatric practitioner office-based setting

### Adib Salim (adibsalim1@gmail.com)

#### ATS, Bergamo, Italy

Point of care ultrasound (POCUS) modality offers a means of improving access to diagnosis and appropriate early treatment to achieve better quality of life. POCUS is increasingly being performed by pediatric primary care in office-based practice and has applications throughout the spectrum of different pathologies, including cardiac applications (acute illness, pericardiac effusion, ventricular function and post-operative follow-up). The skill is relatively easy acquired. However, the lack of exposure of this focused approach in most pediatric training programs in Italy remains a major obstacle. Observational case of three months old infant presented with unexplained left ventricule (LV) wall thickness detected incidentally by cardiac POCUS, which subsequently revealed to have Pompe disease. POCUS cardiac examination performed by primary care pediatric practitioner in ambulatory-based setting practice. Ventricular dimensions were obtained in the cardiac 4 chamber view (m-mode & 2 D recording). It is important that frontline paediatrician equipped with POCUS consider the diagnosis of Hypertrophic Cardiomyopathy even if mild LV wall thickness was present on initial cardiac sonogram. Hypertrophic cardiomyopathy (HCM) is a genetically heterogeneous disorder with a large number of genes involved in disease causation (200 mutations in 10 genes).

Storage disorders and metabolic defects predominate in childhood HCM, and usually are recessive genetic defects, therefore, it is very important recognize particular echo-features of each HCM phenotype in order to plan the correct treatment and to improve patients’ quality of life and survival. POCUS in HCM setting may be used to enhance patient decision making regarding pursuit of targeted genetic panel testing.

## A82 Carbohydrate and human milk

### Guglielmo Salvatori, Silvia Foligno

#### Department of Medical and Surgical Neonatology, Bambino Gesù Children's Hospital, IRCCS, Piazza Sant’Onofrio 4, 00165 Rome, Italy

##### **Correspondence:** Guglielmo Salvatori (gulgliemo.salvatori@opbg.net)

**Background**

Human milk (HM) contains a great variety of different carbohydrates: monosaccharides (glucose, galactose), polyols (myo-inositol and glycerol), fucose, sorbitol, oligosaccharides and peptide or protein-bound carbohydrates (N-acetylglucosamine)[1, 2].

**Materials and Method**

We performed a review to discover and underline the role of carbohydrates in HM.

**Results**

Lactose, the predominant carbohydrate, is present in the highest concentration in HM compared to any other species. It is in the colostrum in lower concentrations than in mature milk, and reaches its highest concentration in the fourth seventh month of lactation[3]. It’s hydrolyzed by lactase in glucose and galactose. Usually, an infant ingesting 150 ml of milk /Kg/day receives 10 g of lactose/Kg/day, which ensures at least 4 mg/Kg/min of glucose, considered as an optimal rate[2]. One of the mainly roles of lactose is to prevent rickets increasing the absorption of calcium and the galactose is essential to the production of cerebroside, which contributes to the CNS development[3]. Human milk oligosaccharides (HMO) are the third largest component in breast milk. Their concentration is about 10 times greater than in cow milk[2]. There are over 200 different types and each contains between 3 to 22 saccharide units per molecule, in varying different sequences and orientations. The monosaccharides are L-fucose, D-glucose, D-galactose, N-acetylglucosamine and N-acetylneuraminic acid. HMOs act as prebiotics by providing a metabolic substrate for the growth of potentially beneficial bacteria. The structure-dependent effects of HMOs depend on the infant and mother. Two genes are important for the HMO profile that the mother produces, the Secretor and Lewis blood group genes. HMOs have protective effects against infectious agents[4], inhibiting the link with the carbohydrates present on intestinal epithelial cells. HMOs may also have indirect effects on the infant microbiome by modulating epithelial and immune cell responses[3, 5]. HMOs may play a role in the prevention of necrotizing enterocolitis, especially through a HMO, the disialyllacto-Ntetraose (DSLNT) [6]. In addition it has been suggested that the different composition of HMO in mother's milk may influence infant growth and body composition[7]. Finally, fructose is present in HM at very low level, but may be transmitted to the infant, influencing the growth and body composition at six months, but it could also impact the obesity development in later childhood[8].

**Conclusions**

This review highlights the extraordinary qualities of breast milk as it contains elements that contribute to the growth of the child and the prevention of infections and metabolic diseases.

**References:**

1. Jozwik M, Jozwik M, Teng C, Jozwik M, Battaglia FC. Human breast milk sugars and polyols over the first 10 puerperium days. Am J Hum Biol. 2013;25:198-204.
2. Lawrence RA, Lawrence RM. Breastfeeding. A guide for the medical profession*.*8^th^ edition. New York: Elsevier; 2016.
3. Andreas NJ, Kampmann B, Mehring Le-Doare K. Human breast milk: A review on its composition and bioactivity. Early Hum Dev. 2015;91:629-635.
4. Bode L.The functional biology of human milk oligosaccharides. Early Hum Dev. 2015;91**:**619-622.
5. Kulinich A, Liu L. Human milk oligosaccharides: The role in the fine-tuning of innate immune responses. Carbohydr Res. 2016;432:62-70.
6. Lars Bode. Human milk oligosaccharides at the interface of maternal–infant health. Breastfeeding Medicine. 2018;13:S7-S8.
7. Alderete TL, Autran C, Brekke BE, Knight R, Bode L, Goran MI, Fields DA. Associations between human milk oligosaccharides and infant body composition in the first 6 mo of life. Am J Clin Nutr. 2015;102:1381-1388.
8. Goran MI, Martin AA, Alderete TL, Fujiwara H, Fields DA. Fructose in breast milk is positively associated with infant body composition at 6 months of age. Nutrients. 2017;9:146.

## A83 Definition of sugars in the regulations and in their labeling in food

### Marco Silano (marco.silano@iss.it)

#### U.O. Alimentazione, Nutrizione e Salute, Dipartimento di Sicurezza Alimentare, Nutrizione e Sanità Pubblica Veterinaria, Istituto Superiore di Sanità, Roma, Italy

The European Commission White Paper on Nutrition highlighted sugars, along with saturated fat and sodium, as nutritional element of importance to public health. In this context, the Regulation EC 1169/2011, fully applied in the European Union Member States from January 2014, sets that the indication of carbohydrates and sugar content in the nutrition declaration in the labeling of pre-packed food is mandatory. The Annex I of the above-mentioned Regulation states that ‘carbohydrate’ means any carbohydrate which is metabolized by humans and includes polyols; and ‘sugars’ means all monosaccharides and disaccharides present in food, excluded polyols. The amount of these two classes of nutrient has to be indicated in grams. This amount can be indicated also as percentage of guideline daily amount, at producer’s discretion.

The attention of consumers for the effect of dietary sugars on their health has been significantly growing in the last years. Consequently, the producers have been introducing in the market, food products claimed as ‘sugar-free’ and ‘low sugar’. The first claim may only be made where the product contains no more than 0,5 g of sugar per 100 g or 100 ml, the latter only where the product contains no more than 5g of sugar per 100 g for solids or 2,5 g of sugar per 100 ml for liquids, as stated in the Reg. EC 1124/2006. The claim ‘with no added sugar’ and any claim likely to have the same meaning for the consumer, may only be made where the product does not contain any added mono- or disaccharides or any other food used for its sweetening properties. If sugars are naturally present in the food, the following indication should also appear on the label: ‘contains naturally occurring sugars. The list of ingredients has to list any substance or product, including flavourings, food additives and food enzymes, and any constituent of a compound ingredient, used in the manufacture or preparation of a food and still present in the finished product, even if in an altered form. The ingredient list therefore, might help the consumers to identify the source of sugars in a food product.

## A84 Sugar and dental health

### Laura Strohmenger (laura.strohmenger@unimi.it)

#### Department of Biomedical, Surgical and Dental Sciences, Dental Clinic G. Vogel, Milano, Italy

Caries is one of the most widespread chronic diseases in the world and is a multifactorial infectious disease. The World Health Organization (WHO) therefore urges the implementation of national epidemiological studies to monitor the state of oral health in specific population groups divided by age.

The etiological factors that contribute to the development of caries are many. The disease, in fact, develops through a complex interaction over time between acidogenic bacteria and fermentable carbohydrates introduced with the diet and factors related to the host, such as saliva. To these factors are added others such as socio-economic status, the use of remineralizing agents, etc.

The cariogenic bacteria that make up the biofilm need carbohydrates to live and reproduce. The metabolism of these substances, especially simple carbohydrates, produces weak acids that cause the demineralization of hard dental tissues, due to the clinical signs of the disease.

We must commit ourselves to using in our diet sweeteners that have an important cario-preventive activity, even in the long term, first of all xylitol, an activity that is performed through the reduction of the concentration of the Streptococci of the mutans group and a consequent reduction of the levels of lactic acid.

## A85 Treating children obesity as a chronic disease: let us adopt the principles of therapeutic education and motivational interviews

### Rita Tanas^1^, Vita Cupertino^2^, Begoña Gil^3^, Maria Marsella^4^, Giampaolo De Luca^5^

#### ^1^Pediatric Endocrinologist, SIP Adolescent Study Group, Ferrara, Italy; ^2^Community Pediatrician, SIP Adolescent Study Group, ASP Cosenza, Italy; ^3^Plan Integral de Obesidad Infantil de Andalucía, Servicio Andaluz de Salud, Consejería de Salud de la Junta de Andalucía, Sevilla, Spain; ^4^Pediatric Unit, S. Giuseppe Moscati Hospital, Avellino, Italy; ^5^Family Pediatrician, SIP Adolescent Study Group, Cosenza, Italy

##### **Correspondence:** Rita Tanas (tanas.rita@tin.it)

Prevalence of obesity and consequences on physical and psychological health are more and more worrisome as reviews the literature on prevention and treatment are poor and with modest results [1-3].

The scientific community interrogates itself on how to manage the situation. In the past years new approaches have been proposed [4]: teach professionals new strategies to promote changes, work in networks and face the stigma of weight [5].

For the first point we proposed therapeutic education (TE)[6], a treatment method born in France and adopted by the World Health Organization to manage chronic diseases; the USA, Spain and other countries propose the motivational interview (MI), borrowed from addiction care. The two approaches have been included in various guidelines and recommendations [7], but not in daily clinical practice in primary and second and third level healthcare [8].

These methods share several points: a good therapeutic relationship between professionals and patients, peer cooperation among the first, experts of the disease, and the second, experts of their own disease. However, such methods do not reconcile with the persistent managerial attitude of the professional [9].

Despite numerous currents that invite professionals to put the patient and his empowerment/engagement at the center of treatment [10], professionals continue to prefer an increasingly technological medicine. Chronic diseases, like obesity, which involve the whole person and his experiences in both causes and consequences, are the most penalized and, when stigmatized, the worst treated [11].

Without diminishing psychological disciplines, it may be useful to simplify approaches which are respectful of the person: focus during academic studies on principles and simple applicative tools. In few hours it is possible to improve the health professional’s approach to this chronic disease and create a network of competence and knowledge. Parents and children to achieve and sustainably maintain the necessary lifestyle changes could benefit more from the harmony in family, school, friend and professional support [12].

Primary healthcare pediatricians play an important role in early detection of problems and their non-judgmental communication, and, when necessary, in offering information on the natural course of obesity. It is a responsibility to share with all caring figures that rotate around the child and with pediatric specialists. All the figures should work in synergy. No one should ignore the weight problem, nor judge and stigmatize the family [13].

**References**

1. Kobes A, Kretschmer T, Timmerman G, Schreuder P. Interventions aimed at preventing and reducing overweight/obesity among children and adolescents: a meta-synthesis. Obes Rev. 2018. doi: 10.1111/obr.12688
2. Elvsaas IKØ, Giske L, Fure B, Juvet LK. Multicomponent lifestyle interventions for treating overweight and obesity in children and adolescents: a systematic review and meta-analyses. J Obes. 2017;2017:5021902.
3. Peirson L, Fitzpatrick-Lewis D, Morrison K, Ciliska D, Kenny M, Usman Ali M, Raina P. Prevention of overweight and obesity in children and youth: a systematic review and meta-analysis. CMAJ Open. 2015;3:E23-33.
4. Dietz WH, Baur LA, Hall K, Puhl RM, Taveras EM, Uauy R, Kopelman Pet. Management of obesity: improvement of health-care training and systems for prevention and care. Lancet. 2015;385:2521-33.
5. Dietz WH. The need for people-first language in our Obesity journal. Obesity. 2015;23:917.
6. Tanas R, Marcolongo R, Pedretti S, Gilli G. A family-based education program for obesity: a three-year study. BMC Pediatr. 2007;7:33.
7. Barlow SE; Expert Committee. Expert committee recommendations regarding the prevention, assessment, and treatment of child and adolescent overweight and obesity: summary report. Pediatrics. 2007;120:4:164-92.
8. Petrin C, Kahan S, Turner M, Gallagher C, Dietz WH. Current attitudes and practices of obesity counselling by health care providers. Obes Res Clin Pract. 2017;11:352-359.
9. Flodgren G, Gonçalves-Bradley DC, Summerbell CD. Interventions to change the behaviour of health professionals and the organisation of care to promote weight reduction in children and adults with overweight or obesity. Cochrane Database Syst Rev. 2017;11:CD000984.
10. Johnson KE, Mroz TM, Abraham M, Figueroa Gray M, Minniti M, Nickel W, Reid R, Sweeney J, Frosch DL, Ness DL, Hsu C. Promoting patient and family partnerships in ambulatory care improvement: a narrative review and focus group findings. Adv Ther. 2016;33:1417-39.
11. FitzGerald C, Hurst S. Implicit bias in healthcare professionals: a systematic review. BMC Med Ethics. 2017;18:19.
12. Schalkwijk AA, Bot SD, de Vries L, Westerman MJ, Nijpels G, Elders PJ. Perspectives of obese children and their parents on lifestyle behavior change: a qualitative study. Int J Behav Nutr Phys Act. 2015;12:102.
13. Tanas R, Gill BB, Caggese G, Baggiani F, Valerio G, Corsello G. Professional stigma on weight in the pediatric care in Italy and Andalusia: recognize it to successfully treat obesity. J Obes Ther. 20171:1.

## A86 Learning how to communicate the diagnosis of obesity in childhood with the collaborative negotiation interview

### Rita Tanas^1^, Serenella Castronuovo ^2^, Vita Cupertino^3^, Natale Lavia^4^, Maria Marsella^5^, Giampaolo De Luca^6^

#### ^1^ Pediatric Endocrinologist, SIP Adolescent Study Group, Ferrara, Italy; ^2^Family Pediatrician, SIP Adolescent Study Group, Nettuno (RM),Italy; ^3^Community pediatrician, SIP Adolescent Study Group, ASP Cosenza, Italy; ^4^Family Pediatrician, SIP Adolescent Study Group, Cosenza, Italy; ^5^Pediatric Unit, S. Giuseppe Moscati Hospital, Avellino, Italy; ^6^Family Pediatrician, SIP Adolescent Study Group, Cosenza, Italy

##### **Correspondence:** Rita Tanas (tanas.rita@tin.it)

From the 2007 recommendations of the American Academy of Pediatrics the motivational interview (MI) has become an official tool for prevention and treatment of obesity in childhood in many countries, although lacking in strong evidence of efficacy[1,2]. Developed for addictions, the MI is a transversal instrument for treatment of several diseases, especially those in which the active involvement of patients and their families is irreplaceable, as obesity. In the 2007 document, a 15 minute example interview was added to facilitate adaptation to the primary pediatric healthcare clinic for the lifestyle improvement. Later, the professionals evidenced difficulty in adopting it with families with a consequent feeling of inefficacy and failure of both professionals and families [3-4]. Fear of failure pushes professionals to exploit families’ feelings of guilt, stressing the metabolic and cardiovascular risks of obesity. However, learning through negative emotions is not efficacious: everyone learns more with the teacher’s smile and the student’s alliance, than with fear of error [5].

In fact the MI does not easily adapt from treatment of addictions in adults to the pediatrician and the dyad parents-children with obesity. Some experts, like Dyane Tyler, tried to facilitate adaptation associating the principles of MI to those of Brazelton’s collaborative negotiation, otherwise well known to many American pediatricians[6,7]

Her work has suggested the idea of using a simulation to train primary care pediatricians (Table 1-2).

In fact, only these pediatricians can exploit the alliance with parents and children and transform motivation from apparent Precontemplation to Determination, bringing to a project of Action (Table 3) [8].

If these pediatricians, although adequately formed, withdraw from their role, they become an insurmountable barrier to treatment, that no skilled team can overcome.

Reviews of literature are with modest results [9-10]. The scientific community interrogates itself on the correct allocation of resources. It would be useful to invest in adaptation of these approaches to childhood; push primary care pediatricians to treat obesity with more confidence and determination; form them to a MI reduced to its principle: “absolute respect for patient’s freedom of choice”, and to simple tools: open questions, simple and complex reflective listening, which should be respectful and prudent, while continuing to be pediatricians. It is not necessary to become counselors.

All this could open to unimaginable successful scenarios.

**References**

1. Davis MM, Gance-Cleveland B, Hassink S, Johnson R, Paradis G, Resnicow K. Recommendations for prevention of childhood obesity. Pediatrics. 2007;120:S229-53.
2. Miller WR, Rollnick S. Motivational interviewing: Preparing people for change. 2° edition. New York: Guilford Press. 2002
3. Klein JD, Sesselberg TS, Johnson MS, O'Connor KG, Cook S, Coon M, Homer C, Krebs N, Washington R. Adoption of body mass index guidelines for screening and counseling in pediatric practice. Pediatrics. 2010;125:265-72.
4. Petrin C, Kahan S, Turner M, Gallagher C, Dietz WH. Current attitudes and practices of obesity counselling by health care providers. Obes Res Clin Pract. 2017;11:352-359.
5. Ledoux J. The emotional brain (the mysterious underpinnings of emotional life). New York: Simon & Schuster. 1998.
6. Tyler DO, Horner SD. Family-centered collaborative negotiation: a model for facilitating behavior change in primary care. J Am Acad Nurse Pract. 2008;20:194-203.
7. Brazelton TB, O’Brien M, Brandt KA. Combining relationships and development: applying touchpoints to individual and community practices. Infants and Young Children,1997;10:74–84.
8. Prochaska JO, DiClemente CC, Norcross JC. In search of how people change. Applications to addictive behaviors. Am Psychol. 1992;47:1102-1114.
9. Mühlig Y, Wabitsch M, Moss A, Hebebrand J. Weight loss in children and adolescents. Dtsch Arztebl Int. 2014;111:818-24.
10. Sim LA, Lebow J, Wang Z, Koball A, Murad MH. Brief primary care obesity interventions: a meta-analysis. Pediatrics. 2016;138:e2016014.

**Table 1 (abstract A86).** Motivational Interview (MI) strategies according to the Touchpoints [6]

MI strategies	Touchpoint approach
Set the theme to be addressed now: the agenda	The agenda is set with parents and child, making sure they identify which health behaviors to address.
Make decisions and define goals	Parents and child define goals to achieve between visits.
Evaluate motivation and confidence in being able to carry out the journey	The professional evaluates child and parent motivation and confidence in achieving goals on a VAS scale from 0 to 10. Points are used to implement commitment and identify strategies to overcome eventual barriers to change.
Exchange information in a non-judging manner	Support parent’s positive qualities and parent-child and parent-child-pediatrician relationships.

**Table 2 (abstract A86).** Characteristics of the Collaborative Negotiation Simulation

Tools	Motivational Interview [2]	Touchpoint [7]	
Actors	PHP^a^	Parents	Child
Objectives of the PHP formation	Communicate the diagnosis without judgment or stigma	Promote evolution from the precontemplation phase to the determination phase	Support parents’ competences and good intentions in order to build together their own project
Themes of the PHP formation	How to make diagnosis	How to comunicate diagnosis	Which objectives to set: • Behaviors’ change, • BMI^b^ zscore’s decrease.

^a^Primary Healthcare Pediatricians (PHP)

^b^BMI Body Mass Index

**Table 3 (abstract A86).** The six phases of change adapted to prevention/treatment of childhood overweight / obesity: thoughts and actions adequate to the family’s stage of motivation. Modified from Prochaska, Di Clemente [8]

Phase	Patient and family position	Patient and family actions	Therapists actions
Pre-contemplation	They don’t think or accept there is a problem.	They are not interested in moving more and eating better in the following 6 months.	Provide information that increases awareness without judging or blaming.
Contemplation	Ambivalence towards change.	They have not decided whether to move more and eat better in the following 6 months.	Ask the pros and cons and reinforce the reasons for change.
Determination	They have decided and are making an action plan.	They are planning to move more and eat better in the following 30 days.	Help them find acceptable, easy and effective strategies.
Action	They are implementing the treatment plan.	They have recently started moving more and eating better (< 6 months).	Help them monitor change, evaluate efficacy of strategies, support themselves, and face their barriers.
Maintenance	They have already realized their plan for some time.	They have become more active and they eat better (> 6 months).	Help them monitor change, evaluate efficacy of strategies, support themselves, and face their barriers.
Relapse	They are no longer interested in the treatment plan.	They abandoned the plan and resumed previous behaviors	Support them in reducing demoralization, learning from mistakes and restarting the process of change.

## A87 EEG/EEG as a valuable tool to study brain development in extremely preterm infants

### Maria L Tataranno^1^, Nathalie HP Claessens^1^, Pim Moeskops^1,2^, Mona C Toet^1^, Karina J Kersbergen^1^, Giuseppe Buonocore^3^, Ivana Išgum^2^, Alexander Leemans^2^, Serena Counsell^4^, Floris Groenendaal^1^, Linda S deVries^1^, Manon JNL Benders^1^

#### ^1^Department of Neonatology, Wilhelmina Children's Hospital, and Brain Center Rudolf Magnus, University Medical Center Utrecht, Utrecht, The Netherlands; ^2^Image Sciences Institute, University Medical Center Utrecht, Utrecht University, Utrecht, The Netherlands; ^3^Department of Molecular and Developmental Medicine, University of Siena, Siena, Italy; ^4^Centre for the Developing Brain, King's College London, London, UK

##### **Correspondence:** Maria L Tataranno (M.L.Tataranno-2@umcutrecht.nl)

**Background:** Prematurity is the leading cause of death in the neonatal period and a high percentage of survivors will experience long term neurodevelopmental disabilities. Extremely preterm infants (born ≤28 weeks of gestation) have 25-50% of risk for altered brain development, and subsequent abnormal neurodevelopmental outcome. Magnetic resonance imaging (MRI) is currently used for the assessment of neonatal brain injury and development. However, MRI is expensive and does not allow bedside sequential monitoring. Therefore, other early biomarkers for prediction of brain injury and maturation are needed. Amplitude-integrated electroencephalography (aEEG) and multi-channel EEG may be useful tools to monitor brain function abnormalities in this high-risk population [1]. I will present a study aiming to investigate the relation between early brain activity and structural (growth of the cortex and cerebellum) and white matter microstructural brain development.

**Materials and methods:** Thirty-three preterm neonates (gestational age 26±1 weeks) without major brain abnormalities were continuously monitored with electroencephalography during the first 48 h after birth. The rate of spontaneous activity transients per minute (SAT rate) and the inter-SAT interval (ISI) in seconds per minute were calculated using an in-house developed program (SignalBase^®^). Infants underwent brain magnetic resonance imaging around 30 (mean 30.5; min: 29.3-max: 32.0) and 40 (41.1; 40.0-41.8) weeks of postmenstrual age. Increase in cerebellar volume, cortical gray matter volume, gyrification index, fractional anisotropy (FA) of posterior limb of the internal capsule, and corpus callosum (CC) were measured.

**Results:** SAT rate was positively associated with cerebellar growth (*p value*=0.01), volumetric growth of the cortex (*p value* =0.027), increase in gyrification (*p value* =0.043), and increase in FA of the CC (*p value* =0.037). ISI was negatively associated with cerebellar growth (*p value* =0.002) [2].

**Conclusions:** aEEG/EEG can play an important role in monitoring brain development. Increased early brain activity is associated with cerebellar and cortical growth structures with rapid development during preterm life. Higher brain activity is related to FA microstructural changes in the CC, a region responsible for interhemispheric connections. The present study underlines the importance of brain activity for microstructural brain development. In the future, the opportunity to combine aEEG/EEG with other physiological data and additional tools will provide clinicians a wider overview of brain health status in the neonatal population.

**References**

1. Pavlidis E, Lloyd RO, Boylan GB. EEG-A valuable biomarker of brain injury in preterm infants. Dev Neurosci. 2017; 39:23-35.

2. Tataranno ML, Claessens NHP, Moeskops P, Toet MC, Kersbergen KJ, Buonocore G, Išgum I, Leemans A, Counsell S, Groenendaal F, de Vries LS, Benders MJNL. Changes in brain morphology and microstructure in relation to early brain activity in extremely preterm infants. Pediatr Res. 2018;83:834-842.

## A88 The limping child

### Renato M Toniolo, Susanna Rivelli

#### Department of Traumatology, Bambino Gesù Pediatric Hospital, I.R.C.C.S., Rome, 00165, Italy

##### **Correspondence:** Renato M Toniolo (renatomaria.toniolo@opbg.net)

Limp is defined as any deviation from normal age-appropriate gait. Hence, it is mandatory to know the normal gait related to the age of patient. Limping is a frequent symptom in children and adolescents and is a common reason for them to present to emergency department (ED) or outpatient clinic. The diagnosis can be often challenging for pediatric or orthopedic physicians. Indeed, a great number of diseases could be the cause of that sign: from trivial to life threatening. Only a systematic and careful history collection and clinical evaluation can rule out some etiologic factors and guide towards the most appropriate diagnostic path. The goal is to reach quickly the diagnosis without exposing the child to invasive, (e.g., multiple X rays, CT or biopsy), unnecessary and sometimes expensive - time consuming (e.g., MRI) diagnostic exams. Etiology could be acute (from contusion to fracture) or chronic traumas (overuse injuries), infections (osteomyelitis and septic arthritis), inflammation (from transient synovitis to rheumatic diseases), neoplasms (benign and malignant), and many other congenital and developmental conditions (e.g. neglected developmental hip dysplasia in the walking age, Legg-Calvè-Perthes disease, slipped capital femoral epiphysis, etc.). The localization varies from hip to foot, but also the spine can be involved (e.g., diskitis or spondylolysis) as well as bony, articular, soft tissue, intra-abdominal conditions. Age is important to direct hypothesis because some pathologies are more frequent during childhood or adolescence. Onset modality (acute, chronic or gradually worsening) and timing (diurnal, nocturnal or activity-related) are key elements for the diagnosis of specific conditions. The child could present antalgic or non-antalgic gait (sometime evident and peculiar e.g. toe walking, Trendelenburg or steppage gait) and some patient could completely refuse weight bearing. The first blood exams are to be the easiest (CBC, ESR, CPR) and only if history or clinical exam is strongly suggestive second level exams are to be performed (synovial fluid analysis, coagulation profile, blood culture, etc.). Imaging is important and starts with X-rays and ultrasounds. CT, MRI and bony-scan are indicated only in selected cases. Non-musculoskeletal conditions can cause limping. A systematic approach is mandatory to rule out or to identify the severest causes and reach quickly the exact diagnosis to avoid long-term morbidity.

**References**

1. Alexander JE, FitzRandolph RL, McConnell JR. The limping child. Curr Probl Diagn Radiol. 1987;16:229-70.
2. Fischer SU, Beattie TF. The limping child: epidemiology, assessment and outcome. J Bone Joint Surg Br 1999;81:1029e34.
3. Barkin RM, Barkin SZ, Barkin AZ. The limping child. J Emerg Med. 2000;18:331-339.
4. Leung AKC, Lemay JF, The limping child. J Pediatr Health Care. 2004;18, 219-223.
5. Kocher MS, Bishop JA, Weed B, Hresko T, Millis MB, Kim YJ, Kasser JR. Delay in diagnosis of slipped capital femoral epiphysis. JR Pediatrics. 2004;113;e322.
6. Reed L, Baskett A, Watkins N. Managing children with acute non-traumatic limp: the utility of clinical findings, laboratory inflammatory markers and xrays. Emerg Med Australas. 2009;21:136-142
7. Lehman PJ, Carl RL. Growing pains: when to be concerned. *Sports Health*. 2017:*9*:132-138.
8. Lampasi M, Antonioli D, Donzelli O, The limping child clinical pediatrics 2012. 51; 907–916.
9. McCanny PJ, McCoy S, Grant T, Walsh S Implementation of an evidence based guideline reduces blood tests and length of stay for the limpingchild in a paediatric emergency department. Emerg Med J 2013;30:19–23.
10. Naranje S. Sameer Dm, Sawyer Jr A systematic approach to the evaluation of a limping child. Amer Fam Phy. 2015; 92.
11. Waseem M, Kumari D, Toledano T. Fever and hip pain not always due to a septic hip. Pediatr Emerg Care. 2017.
12. Raam R, Jhun P, Bright A,; Herber M. Limping child? Think LIMPSS Ann Emerg Med. 2016;67:297-300.

## A89 Phytotherapy and pediatrics: a focus on clinical applications possibilities

### Gianfranco Trapani (gf.trapani@tin.it)

#### Pediatrician, ASL 1 Sanremo (IM), Sanremo, Italy

Phytotherapy consists in the usage of the plant as a whole (phytocomplex) with the purpose of curing diseases that affect children and adults. The active substances contained within the phytocomplex act in synergy, rather than in isolation, with the known specific functions [1] (i.e.Acetylsalicylic acid).

As expert phytotherapy pediatricians, we strive to avoid that our patients retrieve their knowledge from sources of information such as family, community and social media [2]. The usage of phytotherapy in pediatrics should not only aim to safeguard this information and traditional experience, but also the continuous study and research of the specific topic.

Guidelines have been developed by the Italian Federation of Pediatricians to ensure a safe phytotherapic prescription [3].

Focusing on a group of oncologic pediatric patients in the Netherlands, it has been observed that 42,4% of families make use of Complementary and Alternative Medicine (CAM). The most prominent of these being homeopathy 18,8%, followed by food supplements 11,5%, phytotherapy and herbal medicine 6,6%. Unfortunately only one third of the parents had discussed CAM use with their pediatric oncologist [4]. Our aim is to avoid these behavior amongst doctors and patients.

Phytotherapic medicine, such as Pelargonium sidoides (EPs 7630), has been proved effective for upper respiratory tract diseases. In seven studies with 1067 children <6 years EPs 7630 phytotherapy was significantly superior to placebo in reducing symptom intensity and time until complete recovery in patients with acute bronchitis, tonsillopharyngitis and rhinosinusitis. These clinical studies showed that EPS 7630 is effective, safe and well-tolerated in children under 6 years of age and with acute respiratory tract infections [5].

In breastfed colicky infants a standardized extract of Matricariae recutita, Foeniculum vulgare and Melissa officinalis decreased crying time in 85.4% of the subjects treated and in 48.9% of the cases under placebo. No side effects were reported. [6]

There are more controversial points regarding the galactagogue effect of phytotherapy on breast milk production and prolactin secretion. Nevertheless, recent studies support the hypothesis of phytotherapy beneficial effects in mothers of preterm babies who are treated in neonatal intensive care units. The consumption of galactagogue stinging nettle has been proven to increase lactation and prevent lack of human milk without any adverse effect [7].

Hawthorn pulp and seed extracts act on anxiety level and nociceptive perception. This plant has also been traditionally used to treat stress, nervousness, sleep disorders, and pain control [8].

Hypericum perforatum (St. John’s Wort), Passiflora incarnata (Passionflower), and Valeriana officinalis (Valerian) are administered individually or in combination for children depression, school/examination anxieties, further anxieties and sleeping problems [9-10].

The modulation of the immune system and antiviral interventions such as Echinacea (Angustifolia e Purpurea) might reduce the risk of recurrences of respiratory tract infections. Possibly the development of complications relates not only to the presence of viruses but also to immune function [11].

**References**

1. Falzon C, Balabanova A. Phytotherapy: an introduction to herbal medicine. Primary Care. 2017;44:217-227.
2. Freire C, Barbosa L, Costa J, Santos R, Santos A. Phytotherapy in pediatrics: the production of knowledge and practices in Primary Care. Rev Bras Enferm. 2018;71:637-645.
3. Federazione Italiana Medici Pediatri. Linee Guida Fitoterapia FIMP. [https://www.fimp.pro/index.php/cam-complementary-and-alternative-medicine/118-linee-guida-fitoterapia-fimp]. Accessed on 16 July 2018.
4. Singendonk M, Kaspers G, Naafs-Wilstra M, Meeteren A, Loeffen J, Vlieger A. High prevalence of Singendonk M, Kaspers G, Naafs-Wilstra M, Meeteren A, Loeffen J, Vlieger A. High prevalence of complementary and alternative medicine use in the Dutch pediatric oncology population: a multicenter survey. Eur J Pediatr. 2013;172:31-37.
5. Kamin W, Funk P, Seifert G, Zimmermann A, Lehmacher W. EPs 7630 is effective and safe in children under 6 years with acute respiratory tract infections: clinical studies revisited. Curr Med Res Opin. 2018;34:475-485.
6. Savino F, Cresi F, Castagno E, Silvestro L, Oggero R. A randomized double-blind placebo-controlled trial of a standardized extract of Matricariae recutita, Foeniculum vulgare and Melissa officinalis in the treatment of breastfed colicky infants. Phytotherapy Research: PTR. 2005;2005:335-340.
7. Özalkaya E, Aslandoğdu Z, Özkoral A, Topcuoğlu S, Karatekin G. Effect of a galactagogue herbal tea on breast milk production and prolactin secretion by mothers of preterm babies. Niger J Clin Pract. 2018;21:38-42.
8. Can O, Ozkay U, Oztürk N, Oztürk Y. Effects of hawthorn seed and pulp extracts on the central nervous system. Pharma Biol. 2010;48:924-931.
9. Trompetter I, Krick B, Weiss G. Herbal triplet in treatment of nervous agitation in children. Wien Med Wochenschr. 2013;163:52-57.
10. Ulbricht C, Basch E, Woods J, Karpa KD, Gianutsos G, Nummy K, Seamon E, Smith M, Sollars D, Tanguay-Colucci S, Varghese M, Weissner W, Woods J. An evidence-based systematic review of passion flower (Passiflora incarnata L.) by the Natural Standard Research Collaboration. J Diet Suppl. 2008;5:310-340.
11. Schapowal A, Klein P, Johnston S. Echinacea reduces the risk of recurrent respiratory tract infections and complications: a meta-analysis of randomized controlled trials. Adv Ther. 2015;32:187-200.

## A90 Gluten-free diet: a nutritional trend?

### Riccardo Troncone (troncone@unina.it)

#### Department of Medical Translational Sciences & European Laboratory for the Investigation of Food-Induced Disease, University Federico II, Naples, Italy

Gluten is a family of storage proteins (gliadins and glutenins) that can be found in wheat and in other related cereals, such as rye and barley. Spectrum of gluten-induced pathologies includes celiac disease, IgE-mediated wheat allergy and non-celiac gluten sensitivity [1]. The first two conditions are well characterised as pathogenetic mechanisms, clinical spectrum, diagnostic biomarkers and follow-up are condidered. The third condition, in the absence of effective specific biomarkers, is still uncertain as for prevalence and nosologic autonomy; this is true mostly for gastrointestinal clinical presentations, whose limits with gastrointestinal functional disorders remains undefined. There are further conditions for which a gluten-free diet has been invoked, e.g. neurological diseases, autistic spectrum disorders, psoriasis, fibromyalgia and schizophrenia, but in many of these cases the evidences for gluten-free diet efficacy are, at best, weak [2].

Gluten-free products present a different composition in terms of macro and micronutrients; they contain less iron, zinc, magnesium, calcium, selenium, folates, vitamin B12, vitamin D and fibers. They also present an increased glycemic index and a higher content of carbohydrates and lipids rather than their gluten-containing equivalents [3]. Most important informations on nutritional values of gluten-free diets come from studies conducted on celiac subjects among which a higher amount of saturated fats and simple sugars with a lower intake of fibers and folates has been observed [4].

Despite these issues and a higher cost of this group of dietotherapeutic products, gluten-free diet has been popularly recognized as “healthy”. Recent datas suggest that 30% of Americans would reduce or completely cut gluten from their diets. A special category of individuals is represented by athletes who, on the basis of some examples, have chosen gluten-free diet in order to improve their performances [5]. Gluten-free market is expected to raise more than 15 billions of dollars in 2018 [6]. Despite this growing demand, the evidence of beneficial effects totally lacks. For instance, no effect on weight loss ability has been demonstrated yet [6]. The induction of a glycemic prophile improvement has been claimed but, in fact, gluten-free products, losing their component of whole grains in their processing, are responsible of a postprandial glycemic higher increase [7] and ultimately of a minor protection against cardiovascular diseases [8]. Gluten-free diet is also responsible of a modification of intestinal microbioma with a reduction of prebiotic effect. For all these reasons, other than the risk to overshadow celiac disease diagnosis, to reduce or eliminate gluten from one’s diet without a precise clinical indication should be avoided.

**References**

1. Sapone A, Bai JC, Ciacci C, Dolinsek J, Green PH, Hadjivassiliou M, Kaukinen K, Rostami K, Sanders DS, Schumann M, Ullrich R, Villalta D, Volta U, Catassi C, Fasano A. Spectrum of gluten-related disorders: consensus on new nomenclature and classification. BMC Med. 2012;10:13.

2. El Chammas K, Danner E. Gluten-free diet in nonceliac disease. Nutr Clin Pract. 2011;26:294-299.

3. Vici G, Belli L, Biondi M, Polzonetti V. Gluten free diet and nutrient deficiencies: a review. Clin Nutr. 2016;35:1236-1241.

4. See JA, Kaukinen K, Makharia GK, Gibson PR, Murray JA. Practical insights into gluten-free diets. Nat Rev Gastroenterol Hepatol. 2015;12:580-591.

5. Lis DM, Fell JW, Ahuja KD, Kitic CM, Stellingwerff T. Commercial hype versus reality: our current scientific understanding of gluten and athletic performance. Curr Sports Med Rep. 2016;15:262-8.

6. Gaesser GA, Angadi SS. Navigating the gluten-free boom. JAAPA. 2015;28:1-7.

7. Johnston CS, Snyder D, Smith C. Commercially available gluten-free pastas elevate postprandial glycemia in comparison to conventional wheat pasta in healthy adults: a double-blind randomized crossover trial. Food Funct. 2017;8:3139-3144.

8. Lebwohl B, Cao Y, Zong G, Hu FB, Green PHR, Neugut AI, Rimm EB, Sampson L, Dougherty LW, Giovannucci E, Willett WC, Sun Q, Chan AT. Long term gluten consumption in adults without celiac disease and risk of coronary heart disease: prospective cohort study. BMJ. 2017;2:357:j1892.

## A91 Spirometry: indications and contraindications

### Attilio Turchetta (attilio.turchetta@opbg.net)

#### Sport medicine and lung function lab, Bambino Gesù Children Hospital, IRCCS, Rome, Italy

Spirometry is the most common lung function test done. This test measures how much air is moved in and out of the lung, measuring volumes, and the speed of this movement, measuring the flow. Specific instructions have to be followed to perform an acceptable and repeatable test. Most children can do spirometry since age of 6 years old and, in particular setting, also pre-school children are able to perform this test. Indication to spirometry are related to 1)diagnostic problems: dyspnea, wheezing, cough, cyanosis, chest deformity, unexplained crackles ecc. 2)Monitoring therapeutic interventions or bronchodilator therapy. 3) To describe the course of disease affecting lung function. 4) To monitor persons in occupations with exposure to injurious agents. 5) To monitor for adverse reactions to drugs with known pulmonary toxicity. The following conditions are considered relative contraindications: presence of respiratory tract infection (e.g. influenza), haemoptysis of unknown origin, pneumothorax, aneurysm, uncontrolled hypertension, recent thoracic, abdominal or eye surgery, nausea, vomiting or pain, and confusion or dementia.

## A92 High flow nasal cannula in children

### Nicola Ullmann, Serena Caggiano, Maria G Paglietti, Alessandro Onofri, Renato Cutrera

#### Academic Department of Pediatrics, Bambino Gesù Children's Hospital, IRCCS, Rome

##### **Correspondence:** Nicola Ullmann (nicola.ullmann@opbg.net)

Acute lower respiratory tract infections (LRTI), such as pneumonia and bronchiolitis, are the major causes of morbidity and mortality in young children, and are the most frequent cause of hospitalization. The differences in respiratory physiology between children and adults explain the higher susceptibility of infants to more severe manifestations of respiratory diseases, leading in some cases to blood oxygen desaturation. In children with non hypercapnic hypoxaemia, supplemental oxygen therapy is essential to maintain good level of blood oxygenation. In cases of more severely ill patients with significant respiratory distress and heavy work of breathing, high-flow nasal cannula (HFNC) treatment can be offered to patients. The mild heated and humidified air pressure on a continuous basis allows to keep the airways open, prevents alveolar collapse with the final effect of recruiting more alveoli. Moreover, the heated humidification of the respiratory gas facilitates airway clearance. Thanks to all these mechanisms, HFNC improves patient’s oxygenation, gas exchange and reduce the work of breathing which is extremely important in infants affected by LRTI. HFCN consists in the administration of heated and humidified mixture of air and oxygen at a flow rate higher than the patient’s inspiratory flow [1]. The definition of high flow still need to be defined even though, in infants, high flow rates are considered >2 L/min, while in children, >6 L/min [1]. In clinical practice, some authors suggest to adjust the flow rates on body weight and recommend using 2 L/kg/min, which provides a degree of distending pressure and reduces the work of breathing [2-4]. HFNC should be initiated just if the following conditions are satisfied: 1) paediatric setting with a close monitor of the patient’s clinical course 2) a sufficient number of staff that is well trained to recognize the early signs of failure. Accurate and frequent monitoring during HFNC treatment is very important to ensure its effectiveness and safety. The level and type of monitoring should be proportional to the patient's clinical condition. For patient being treated acutely in-hospital, continuous monitoring is indicated with a pulse-oxymeter or a multichannel cardio-respiratory monitor. A strict clinical observation is also mandatory and it must always assess respiratory rate and fatigue, level of dyspnea, signs of possible respiratory asynchrony, or short-term possible complication of HFNC (i.e. gastric distension, nostril irritation etc...). Arterial blood gas analysis should be assessed after 1–4 h after HFNC is establishment.

**References**

1. Lee JH, Rehder KJ, Williford L, Cheifetz IM, Turner DA. Use of high flow nasal cannula in critically ill infants, children, and adults: a critical review of the literature. Intensive Care Med. 2013,39:247–257.
2. Milési C, Baleine J, Matecki S, Durand S, Combes C, Novais AR, Cambonie G. Is treatment with a high flow nasal cannula effective in acute viral bronchiolitis? A physiologic study. Intensive Care Med. 2013,39:1088–1094.
3. Mayfield S, Bogossian F, O’Malley L, Schibler A. High-flow nasal cannula oxygen therapy for infants with bronchiolitis: pilot study. J Paediatr Child Health. 2014,50:373–378.
4. Pham TM, O’Malley L, Mayfield S, Martin S, Schibler A. The effect of high flow nasal cannula therapy on the work of breathing in infants with bronchiolitis. Pediatr Pulmonol. 2015;50:713-20.

## A93 How to talk about immunizations to the caregivers

### Michele Valente (valentem@tiscali.it)

#### Family Pediatrician and Counselor, ASLRoma1, Roma, Italy

In 2017 in Italy entered in force the Law no. 119 (Legge 31, Luglio, 2017, the ‘Law’) with the aim to implement the National Vaccination and Prevention Plan Act 2017-2019 (‘PNV’), published in the Italian Official Journal, G.U. 18 Febbraio 2017, n.41.

The Law inverted the trend of reduction of the coverage rates of vaccinations of the last years, as resulted from the epidemiological data of National Superior Institute of Health (Istituto Superiore di Sanità, the ‘ISS’). The Law classified 10 vaccines as ‘mandatory’ instead of ‘recommended’, as provided within the previous classification. Moreover, the Law has introduced some new immunizations and has recommended some others, as suggested by international literature and scientific evidences. A recent survey from the ISS shows that the coverage rates of vaccinations have been improved over the whole Italian territory, after the first year from the entry in force of the Law. Nevertheless all the doctors shall take in consideration the feelings, the fears and consciousness of caregivers to reduce hesitancy and conflicts concerning the immunizations, thus improving the implementation of the Law and of PNV.

Creating a good relationship between caregivers and doctors is the best way to achieve the goal of children’s health. In order to change people’s opinion about the risk of vaccination is important to underline the benefits of the latter. In order to make easier for caregivers to understand the relevance for their babies to get immunized, preventing the risks of some preventable illness, the systemic counseling skills seem to be necessary. These skills include “active listening”, the decrease of “communication barrier mode” (as described by Gordon [1]), “the three steps movement”, how to investigate the fears of the caregivers and how to assurance caregivers about the vaccination’s benefits. This approach is not spontaneous and it has to be learnt by the healthcare professionals. This presentation wants to underline also some theoretical aspects of the Systematic Counseling Communication Theory (for instance, the Prochaska-Di Clemente Cycle [2]), the verbal and non verbal communication, the level of relationship and contents of the communication.

This presentation wants to be a starting point for medical doctors, pediatricians and health professionals to improve their communications skills with particular regard to vaccinations of children.

**References**

1. Gordon T. Insegnanti efficaci. Il metodo Gordon: pratiche educative per insegnanti genitori e studenti. Teramo: Giunti Lisciani. 1991.

2. Centro Regionale di Documentazione per la Pomozione della Salute. Gli stadi del cambiamento: storia ,teoria ed applicazioni modello transteoretico di DiClemente e Prochaska. Regione Piemonte. 2nd Edition. 2014. [https://www.dors.it/alleg/0200/ragazzoni_quaderno.pdf] Accessed on 16 July 2018.

## A94 Bordetella pertussis

### Piero Valentini (piero.valentini@unicatt.it)

#### Department of Woman and Child Health, Catholic University of the Sacred Heart, Rome, Italy

Pertussis, or whooping cough, is an acute infection interesting respiratory tract, caused by Bordetella pertussis, but also B. parapertussis and B. bronchiseptica. Its incubation period is 7-10 days and course usually is divided in catharral stage (1-2 weeks), including malaise, sneezing, rhinorrea, lacrimation, mild cough, paroxysmal stage (several weeks) characterized by coughing spells occurring in succession, for difficulties in expelling thick mucus from airways, and resulting in emesis, cyanosis, bulging eyes, lacrimation, distension of neck veins; characteristic is the high-pitched inspiratory whoop following prolonged cough attacks. Infants can show bradycardia, apnea and feeding difficulties. Adolescents can present weight loss (33%), urinary incontinence (28%), syncope (4%) and rib fractures (4%). Convalescent stage begins when paroxysmal attack wanes and lasts 2-3 weeks, but any other respiratory infection can exacerbate pertussis symptoms. Infants hospitalized for pertussis show apnea (50%), pneumonia (20%), seizure (1%) and death (1%) as main complications [1]. Laboratory test used for diagnosis is time- sensitive: in fact, culture and PCR sensitivity decreases from 70% and 80% a 10% and 21%, respectively, from the first two weeks of coughing to the period following the second week from the cough onset. Serology has a low sensitivity during the early stage of the infection; this method is useful mostly for epidemiological purposes [2]. A Cochrane review established that antibiotics neither reduce mortality, nor significantly modify the course of the disease, or prevent complications, but reduce transmission of the disease to other persons. eradicating the bacteria from the nasopharynx, as long as it is administered within six weeks of the onset of cough in younger patients (<12 months), and within three weeks in all other patients. Macrolides are first line antibiotics, cotrimoxazole and clyndamicin are further options. Corticosteroids, bronchodilators, antitussives, and antihistamines are not generally recommended [1]. The current prevention is entrusted to an acellular vaccine containing several major antigenic proteins (pertussis toxin (PT), filamentous hemagglutinin, pertactin (PRN), and 2 fimbrial agglutinogens) , but perplexities about its immunogenicity are increasing owing to a progressive rising of pertussis incidence in many countries, maybe for reduced ability of this vaccine to induce mucosal immunity, fundamental in reducing the duration of nasopharyngeal carriage of B. pertussis and limit person-to-person transmission, although recent data shows that pertussis resurgence is not universal and the geographical variation in trends does not support a single explanation, such as the transition from whole-cell to acellular pertussis vaccines [3-5].

**References**

1. Kline JM, Lewis WD, Smith EA, Tracy LR, Moerschel SK. Pertussis: a reemerging infection. Am Fam Physician. 2013;88:507-514.
2. Lee AD, Cassiday PK, Pawloski LC, Tatti KM, Martin MD, Briere EC, Tondella ML, Martin SW; Clinical Validation Study Group. Clinical evaluation and validation of laboratory methods for the diagnosis of Bordetella pertussis infection: Culture, polymerase chain reaction (PCR) and anti-pertussis toxin IgG serology (IgG-PT). PLoS ONE. 2018;13:e0195979.
3. Plotkin SA. The pertussis problem. Clin Infect Dis. 2014;58:830–3.
4. Gill C, Rhoani P, Thea DMl. The relationship between mucosal immunity, nasopharyngeal carriage, asymptomatic transmission and the resurgence of Bordetella pertussis. F1000Research 2017,6:1568.
5. Domenech de Cellès M, Magpantay FM, King AA, Rohani P. The pertussis enigma: reconciling epidemiology, immunology and evolution. Proc Biol Sci. 2016;283:pii: 20152309.

## A95 Neurodevelopmental screening and monitoring protocol

### Elena Vanadia (elena.vanadia@libero.it)

#### Istituto di Ortofonologia, Centro di diagnosi e terapia per l’età evolutiva accreditato SSN, Roma, Italy

The first 1000 days, from conception to completion of the second year, are fundamental for the future health, both physical and mental. The newborn should find an environment responsive to his needs and attentive to his vulnerabilities in order to best express his potential. In the first two years the bases of future personal and social skills are developed: from protocommunication to language, from intersubjectivity to relationship, from sensoriality to perception, from early mimetic-imitative mechanisms to praxic organization, from the first experiences of autonomy to the neuropsychological and learning functions, from regulation to behavior.

Neurodevelopmental Disorders (NDDs) and in particular the pervasive ones, which include Autism Spectrum Disorders (ASDs), can be identified in terms of specific vulnerabilities and through early indicators already in the first two years of life. The intervention, as a diagnostic-therapeutic process, but above all as assistance (services-school-family) will be individualized. The same signs or symptoms can be ascribed to different matrices, only the correct classification, which in the first years must take into account the differential diagnoses and the development of the correlated framework and / or age disorder, can determine an appropriate therapeutic project.

Hence the importance of a screening and monitoring program that involves pediatricians, early childhood operators and child neuropsychiatrists, aimed at identifying early indicators of vulnerability: by compelting the form it will be possible to identify three degrees of general vulnerability, but also specific areas of delay or atypia. With reference to the concept of screening, once the fragile areas or deviations of development are identified, we will intervene with suggestions aimed at parents and / or with specific in-depth protocols and care.

The ‘neurodevelopmental screening and monitoring protocol’ is composed of 5 blocks of questions divided by age groups (0-3, 4-6, 7-12, 13-18, 19-24 months); each block can be used independently of the others and the quantitative analysis of the corresponding scores will fall within a range (shown at the bottom of the page) according to which the suggested operating choice will be indicated. Within each protocol there will be 'critical questions', that is red flags that even in the presence of a total score under the cut-off will have to alert the attention of the pediatrician or the operator. However, in line with the principle of developmental, characteristic of the first years of life, it will be the progressive compilation of protocols to provide, for each child, his development trajectory.

## A96 Neurodevelopmental disorders and neurodiversity

### Davide Vecchio (davidevecchio@ymail.com)

#### Regional Referral Centre for Rare Genetic and Chromosomal Diseases, Villa Sofia-Cervello Hospital, Palermo, Italy

Investigation into neurodevelopmental disorders (NDD) and neurodiversity spans basic research on cellular and molecular mechanisms, using in vivo and human stem cell-based models, neuroimaging and clinical research on epidemiology, outcome and treatment [1]. During the past decade, advances in genetic research have allowed genome-wide discovery of chromosomal copy-number changes and single-nucleotide changes in patients who developed NDD in childhood. These technological advances - which include array comparative genomic hybridization, single-nucleotide-polymorphism (SNP) genotyping arrays and, next generation sequencing - have transformed the approach to the identification of causative genes and genomic rearrangements and, they are currently applied in the clinical diagnostic arena [2]. However, only the most recent acquisitions – i.e. the whole exome/whole genome massively sequencing technologies - have transformed our understanding on genomic variations and their relevance to health and disease, enabling the further rapid discovery of several single-gene causes in neuroscience as well as across disciplines [3]. Expanding the neurosciences’ knowledge represents one the future major goals in research since these conditions have been highlighted among the most frequent reasons for medical referral and/or diagnostic workup. Indeed, in Western countries NDD - which include according to the DSM-5: attention deficit hyperactivity, autism spectrum, communication, intellectual developmental, motor and specific learning disorders - have an incidence of 1 in 66 to 1 in 100 children and, taken together, they represent one of the main population-wide health burdens [4]. Early diagnosis and appropriate access to interventions have been the only so far demonstrated to gain better long-term outcomes and, to reduce lifetime costs for individuals, families and society [5]. However, despite substantial progress on understanding the role played by deleterious genomic variants and their effector proteins on pathological mechanisms of cognitive disorders, few therapeutic interventions have been proposed so far [6]. Hence, connecting NDD’s clinical and functional data represents a fundamental step in research since only through their physiopathological characterization, we will access on the dynamics that constantly shape and reshape our central nervous system. This approach will bridge the existing gap into their etiologic factors’ incomplete knowledge, and will allow researchers to achieve consistent blueprints to widen the scope of new reliable diagnostic and therapeutic tools.

**References**

1. Kapp SK, Gillespie-Lynch K, Sherman LE, Hutman T. Deficit, difference, or both? Autism and neurodiversity. Dev Psychol. 2013;49:59-71.
2. Mefford HC, Batshaw ML, Hoffman EP. Genomics, intellectual disability, and autism. N Engl J Med. 2012;366:733-743.
3. Wright CF, Fitzgerald TW, Jones WD, Clayton S, McRae JF, van Kogelenberg M, King DA, Ambridge K, Barrett DM, Bayzetinova T, Bevan AP, Bragin E, Chatzimichali EA, Gribble S, Jones P, Krishnappa N, Mason LE, Miller R, Morley KI, Parthiban V, Prigmore E, Rajan D, Sifrim A, Swaminathan GJ, Tivey AR, Middleton A, Parker M, Carter NP, Barrett JC, Hurles ME, FitzPatrick DR, Firth HV; DDD study. Genetic diagnosis of developmental disorders in the DDD study: a scalable analysis of genome-wide research data. Lancet. 2015;385:1305-1314.
4. Baio J, Wiggins L, Christensen DL, Maenner MJ, Daniels J, Warren Z, Kurzius-Spencer M, Zahorodny W, Robinson Rosenberg C, White T, Durkin MS, Imm P, Nikolaou L, Yeargin-Allsopp M, Lee LC, Harrington R, Lopez M, Fitzgerald RT, Hewitt A, Pettygrove S, Constantino JN, Vehorn A, Shenouda J, Hall-Lande J, Van Naarden Braun K, Dowling NF. Prevalence of autism spectrum disorder among children aged 8 years—autism and developmental disabilities monitoring network, 11 sites, united states, 2014. MMWR Surveill Summ. 2018;67:1-23.
5. Lappè M, Lau L, Dudovitz RN, Nelson BB, Karp EA, Kuo AA. The diagnostic odyssey of autism spectrum disorder. Pediatrics. 2018;141:S272-S279.
6. Vissers LELM, Gilissen C, Veltman JA. Genetic studies in intellectual disability and related disorders. Nat Rev Genet. 2016;17:9-18.

## A97 Vitamin D supplementation: do well, do better

### Francesco Vierucci (vieruf@hotmail.it)

#### Pediatric Unit, San Luca Hospital, Lucca, Italy

Vitamin D is a key hormone in the regulation of calcium and phosphorus metabolism. Moreover, the recently suggested role of vitamin D in the development of several non-skeletal diseases reinforced the interest in the promotion of an adequate vitamin D status during pediatric age.

Skeletal actions of vitamin D: nutritional rickets represents the iceberg of vitamin D deficiency. The clinical presentation of rickets is variable but is more likely to be seen during periods of rapid childhood growth, such as infancy and adolescence. However, vitamin D deficiency may present with a spectrum of clinical pictures, representing a continuum ranging from asymptomatic/subtle conditions to overt rickets/osteomalacia [1]. Vitamin D status also represents an important lifestyle factor that influences bone mass acquisition, up to the achievement of peak bone mass [2]. Indeed, long-lasting unrecognized vitamin D deficiency during pediatric age may affect bone health, possibly predisposing to osteoporosis later in life.

Extraskeletal actions of vitamin D: besides its historical skeletal functions, in the last years it has been confirmed that vitamin D plays so-called extraskeletal actions, directly or indirectly regulating up to 1,250 genes. Particularly, vitamin D status has been linked to the pathogenesis of several pathological conditions, including infectious, allergic and autoimmune diseases [3]. Particularly, two recent meta-analyses showed that vitamin D supplementation protected against acute respiratory tract infection [4], and significantly reduced the rate of asthma exacerbations requiring treatment with systemic corticosteroids [5]. At present, more well-conducted trials are needed to confirm the promising role of vitamin D in the promotion of the global health of children.

Vitamin D supplementation: considering the high prevalence of vitamin D deficiency in Italian children, associated also with newly diagnosed cases of nutritional rickets, the Italian Pediatric Society and the Italian Society of Preventive and Social Pediatrics recently published the consensus “Vitamin D in pediatric age” to provide a practical approach to vitamin D supplementation for Italian infants, children and adolescents [6]. Supplementation should be recommended in all infants in the first year of life, independently of the type of feeding. Subsequently, supplementation should be individualized on the basis of the presence of risk factors for vitamin D deficiency. Particularly, supplementation is suggested from the end of fall to the beginning of spring in children and adolescents with reduced sun exposure during summer, while continuous supplementation should be proposed in cases of permanent risk factors for deficiency.

**References**

1. Vierucci F, Del Pistoia M, Randazzo E, Massart F, Federico G. The spectrum of vitamin D deficiency: description of a family. Exp Clin Endocrinol Diabetes. 2017; 125:478-484.
2. Weaver CM, Gordon CM, Janz KF, Kalkwarf HJ, Lappe JM, Lewis R, O'Karma M, Wallace TC, Zemel BS. The National Osteoporosis Foundation’s position statement on peak bone mass development and lifestyle factors: a systematic review and implementation recommendations. Osteoporos Int. 2016; 27: 1281-1386.
3. Hossein-Nezhad A, Holick MF. Vitamin D for health: a global perspective. Mayo Clin Proc. 2013; 88: 720-755.
4. Martineau AR, Jolliffe DA, Hooper RL, Greenberg L, Aloia JF, Bergman P, Dubnov-Raz G, Esposito S, Ganmaa D, Ginde AA, Goodall EC, Grant CC, Griffiths CJ, Janssens W, Laaksi I, Manaseki-Holland S, Mauger D, Murdoch DR, Neale R, Rees JR, Simpson S Jr, Stelmach I, Kumar GT, Urashima M, Camargo CA Jr. Vitamin D supplementation to prevent acute respiratory tract infections: systematic review and meta-analysis of individual participant data. BMJ. 2017; 356: i6583.
5. Jolliffe DA, Greenberg L, Hooper RL, Griffiths CJ, Camargo CA Jr, Kerley CP, Jensen ME, Mauger D, Stelmach I, Urashima M, Martineau AR. Vitamin D supplementation to prevent asthma exacerbations: a systematic review and meta-analysis of individual participant data. Lancet Respir Med. 2017; 5: 881-890.
6. Saggese G, Vierucci F, Prodam F, Cardinale F, Cetin I, Chiappini E, De' Angelis GL, Massari M, Miraglia Del Giudice E, Miraglia Del Giudice M, Peroni D, Terracciano L, Agostiniani R, Careddu D, Ghiglioni DG, Bona G, Di Mauro G, Corsello G. Vitamin D in pediatric age: consensus of the Italian Pediatric Society and the Italian Society of Preventive and Social Pediatrics, jointly with the Italian Federation of Pediatricians. Ital J Pediatr. 2018; 44: 51.

## A98 Holistic multidisciplinary protocol for age assessment of unaccompanied foreigner children

### Raffaele Virdis^1,2,3^, Patrizia Carletti^4,5^

#### ^1^Board “Immigrants and Health Services” of the Health Commission of The Italian Regions Conference, Parma, Italy; ^2^Consultant, GLNBM, Parma, Italy; ^3^Medical school, Parma University, Parma, Italy; ^4^Coordinator of the national Board Immigrants and Health Services” of the Health Commission of The Italian Regions Conference, Roma, Italy; ^5^Observatory on Health Inequalities, Health Department, Marche Region, Ancona, Italy

##### **Correspondence:** Raffaele Virdis (raffaele.virdis@unipr.it)

Recently, the Interregional Board “Immigrants and Health Services” of The Italian Regions Conference, together with the contribution of various Ministries, Scientific Societies, and stakeholders (UNHCR, Save the Children, Caritas), set up a “Protocol for the identification and the holistic multidisciplinary age assessment of foreigner, unaccompanied children”. The produced protocol respects the European directives on human rights and gives clear indications for age assessment (AA), unifying the previous, diverse methods performed in Italy, which mostly used invasive procedures (unethical according to International law courts’ opinions) as ionizing X-rays for bone or dental age.

The protocol underlines the role of the pediatrician of the “National Health Service” in this AA procedure, but also the important and indispensable contribution of professions such as social workers, cultural mediators and child neuropsychiatrist and / or developmental psychologists. This multidisciplinary method avoids that the determination is only based on the degree of physical and pubertal maturation of the alleged minor but also takes into account his/her psychological, social and cultural maturity and also considers the administrative investigations, if the safety of the child and/or his family at home is not endangered. In addition, invasive procedures (X-rays) are always discouraged and allowed only in a few specific cases, well motivated by the examiners.

The AA performed only through medical methods could not give an exact response and, if in the 95% of the cases the possible error is + 2 years, in the remaining 5% could be also + 3-4 years. This range of variability is not acceptable, especially in ages near the legal limit of 18 years.

Moreover, this method guarantees to the alleged minor the respect for his person and his legal protection, and it seems to be the best system, even if the result could be not completely correct because any form of AA is not an “exact science”. Also with this method it is important to indicate a range of the possible age (average plus/minus 2 standard deviations) and it is sufficient that only the lower age (-2sd) falls below 18.

The protocol in comparison with other international ones and with what was done before, confirms its scientific nature and the respect for human rights, providing recourse to invasive tests only in extreme situations and always with the informed consent of the subject. The “Italian law 47/2017” refers to this “multidisciplinary method” as the best way to perform AA.

**References**

1. The European Parliament and the Council of the European Union. Directive 2013/32/eu of the European Parliament and of the Council of 26 june 2013 on common procedures for granting and withdrawing international protection (recast). [https://eur-lex.europa.eu/legal-content/EN/TXT/?uri=celex:32013L0032]. Accessed on 13 July 2018.

2. Aynsley-Green A, Cole TJ, Crawley H, Lessof N, Boag LR, Wallace RM. Medical, statistical, ethical and human rights considerations in the assessment of age in children and young people subject to immigration control. Br Med Bulletin. 2012;102:17-42.

3. Benso L, Milani S. Alcune considerazioni sull’uso forense dell’età biologica. [http://www.asgi.it/wp-content/uploads/public/1_2013_accertamento_eta_materiali.pdf]. Accessed on 13 July 2018.

## A99 A digital secure base: the guided entrance of the child into technology

### Barbara Volpi (barbara.volpi@uniroma1.it)

#### Department of Dinamic and Clinical Psychology, Sapienza, Rome, Italy

The digital revolution imposes a new way of being parents that takes into account the capillary insertion of new technologies in the development of children. This contribution aims to illustrate the theoretical and scientific paradigms underlying digital parenting and to trace a developmental trajectory preventively oriented towards a responsible use of the network. [1]

**References**

1. Volpi B. Genitori digitali. Crescere i propri figli nell'era di internet. Bologna: il Mulino. 2017.

## A100 Altered thyroid function

### Silvana Caiulo, Maria Cristina Vigone, Giovanna Weber

#### Vita-Salute San Raffaele University, Department of Paediatrics, San Raffaele Hospital, Milan, Italy

##### **Correspondence:** Giovanna Weber (weber.giovanna@hsr.it)

Subclinical hypothyroidism (SH) is defined by the presence of TSH levels above the upper limit of the reference range, in the presence of normal FT4 values. The incidence of SH in the pediatric population is about 2,9% [1]. The first diagnostic step in evaluating the thyroid function tests (TFTs) results is to use the correct reference range for age. In fact, the TSH and FT4 values are different according to the age of the patient. In the neonatal period, the TSH and FT4 values are much higher than subsequently. Second, the methods to measure TSH can be different and there are also intra-individual variations. For these reasons, it is important to obtain at least two different TFTs in order to make diagnosis of SH. Third, the blood tests should not be done during illnesses.

With the exclusion of the neonatal period, a TSH value higher than 5 mcU/ml with normal FT4 values can be considered diagnostic of SH. SH can be defined as mild (TSH 5-10 mcU/ml) and severe (TSH values >10 mcU/ml). The next important diagnostic step is to research the etiology. We need to take familiar anamnesis and patient’s medical history, to test anti-thyroid antibodies (anti-tireoperoxidase and anti- thyroglobulin antibodies) and to do a thyroid ultrasound.

We have to differentiate between autoimmune and non-autoiummune forms. Autoimmune SH presents a higher risk of evolution toward overt hypothyroidism compared to non-autoimmune SH (39,1% versus 13,6% after a 3-year follow-up) [2].

Non autoimmune SH can be due to persistent neonatal hyperthyrotropinemia, genetic defects (TSHR, DUOX2, Thyroglobulin variants), iodine deficiency, drugs (anti-epileptic drugs, caffeine..), syndromes (Down syndrome, Turner syndrome, Di George syndrome, Pseudohypoparathyroidism), and obesity. SH in obese children is usually reversible and should be managed with lifestyle measures that encourage weight loss.

Finally, when all the known etiologies have been excluded, we talk about idiopathic SH. Idiopathic SH rarely evolves toward overt hypothyroidism. Wasniewska et al. showed that 88% of patients with idiopathic SH normalized or maintained unchanged their TSH [3]. Idiopathic SH in children seems to be a benign and remitting process, with a low risk of progression to overt thyroid dysfunction [4]. Currently, there is no evidence to recommend treatment for all children with mild asymptomatic SH. The management of a child with SH should be individually tailored on several factors, such as the degree of the TSH elevation, the etiology, the presence of symptoms and risk factors.

^**References**^

1. Lazar L, Frumkin RB, Battat E, Lebenthal Y, Phillip M, Meyerovitch J. Natural history of thyroid function tests over 5 years in a large pediatric cohort. J Clin Endocrinol Metab. 2009; 94, 1678–1682.

2. Radetti G, Maselli M, Buzi F, Corrias A, Mussa A, Cambiaso P, Salerno M, Cappa M, Baiocchi M, Gastaldi R, Minerba L, Loche S. The natural history of the normal/ mild elevated TSH serum levels in children and adolescents with Hashimoto’s thyroiditis and isolated hyperthyrotropinemia: a 3‑year follow‑up. Clin Endocrinol. 2012; 76, 394–398.

3. Wasniewska M, Salerno M, Cassio A, Corrias A, Aversa T, Zirilli G, Capalbo D, Bal M, Mussa A, De Luca F.. Prospective evaluation of the natural course of idiopathic subclinical hypothyroidism in childhood and adolescence. Eur J Endocrinol. 2009; 160, 417–421.

4. Monzani A, Prodam F, Rapa A, Moia S, Agarla V, Bellone S, Bona G. Endocrine disorders in childhood and adolescence. Natural history of subclinical hypothyroidism in children and adolescents and potential effects of replacement therapy: a review. Eur J Endocrinol. 2012; 168, R1–R11.

## A101 International adoptions

### Mauro Zaffaroni^1^, Sara Lovaste^1^, Francesco Tagliaferri^1^, Luigi Maiuri^1^ and GLNBM-SIP ^2^

#### ^1^Department of Pediatrics, Novara, Italy; ^2^Members of GLNBM-SIP (Working Group for Migrant Child of the Italian Society of Pediatrics): G. Zavarise, F. Doro (Negrar–VR), E. Chiappini, L. Galli (Firenze), P. Valentini, D. Pata, D. Buonsenso, G. Salerno, A. Turziani Colonna (Roma-Gemelli), R. Marrone, R. Bosi (Roma-INPM), S.Garazzino, L. Baroero, R. Calzedda (Torino), A.F. Podestà (Milano-S.Carlo), R.Arancio (Milano-S.Paolo), M. Sala, F. Speranza, E. Parolo (Tradate-VA), F. Maschio (Treviso), F. Colonna, L. Casali (S. Vito al Tagliamento-PN), A. Lauriola (Rovereto-TN), G. Ballardini, A. Guala (Verbania), G. Ricci, F. Cipriani, A. Giannetti (Bologna), I. Dodi, V. Maffini (Parma), R Cordiali, L. Santoro (Ancona), G. Lombardi, G. De Michele, M.T. Anzellotti (Pescara), P. Vuilleumier, A. Boccieri (Napoli), D. Bove (Nardò-LE), S. La Placa, M. Giuffré (Palermo), M.A. Pulito (Lecce), G. Veneruso (Fano-PU), R. Virdis (Parma)

##### **Correspondence:** Mauro Zaffaroni (maurozaff@libero.it)

Every year, thousands of adopted children arrive in Italy from all over the world: over 2000 in 2014 and 2015, 1872 in 2016, 1439 in 2017. These children mostly come from the Russian Federation, Colombia, Ethiopia, India, Hungary, Poland, Vietnam and China. According to CAI (Commission for International Adoptions) [1], in the first quarter of 2018, 181 out of 273 adopted children (66%) had special needs: congenital malformations, genetic diseases, or infections (hepatitis, CMV, HIV) that are reported before adoption.

When adopted children arrive in Italy, it is necessary to re-evaluate their health conditions and to verify their vaccination status. For this reason GLNBM-SIP has organized a network of 22 centers in Italy specializing in international adoption where children’s health is evaluated according to a special adoption protocol and targeted to the area of origin [2].

In 2016-2017, GLNBM’s hospitals evaluated 2516 children (76% of the 3310 adopted minors who arrived in Italy during the same period). Many of these children were affected by malformations, had outcomes of congenital infections, parasite infestations or TB disease; several cases reach early puberty soon after adoption. The majority of adopted children were inadequately vaccinated.

In addition to the health evaluation of adopted children, 10 Centers perform training activities for adoptive parents that focus, in particular, on health risk evaluation as well as consultancies for interested institutions, and for local social assistant services.

More recently, in order to respond the increased number of requests from adoptive parents and institutions, some co-workers of the GLNBM started to offer long-distance consultations in cases of children with Special Needs and pathologies declared only at the time of meeting with the adoptive parents.

A network of hospitals, territorial services, Courts for minors, CAI and other agencies is required to effectively accommodate the needs of children and adoptive families. At present in Italy only some Regions and Autonomous Provinces have established regulations concerning the health care of adopted children, however, at the national level there has yet to be any legislation on this. The Italian Society of Pediatrics together with the CAI can play a key role in helping launch concrete actions to promote legislations aimed at protecting the health of adopted children as well as offering training and support to couples in their difficult parental roles.

**References**

1. Commissione per le adozioni internazionali. [http://www.commissioneadozioni.it/IT.aspx?DefaultLanguage=IT]. Accessed on 27 May 2018.

2. Gruppo di lavoro nazionale per il bambino migrante della Società Italiana di Pediatria. [http://www.glnbi.org]. Access on 27 May 2018.

